# Supplementary material for: Crystalline silica-exposed human lung epithelial cells presented enhanced anchorage-independent growth with upregulated expression of BRD4 and EZH2 in autocrine and paracrine manners
Source: PLoS One. 2023 May 5;18(5):e0285354. doi: 10.1371/journal.pone.0285354 (PMC10162546; doi:10.1371/journal.pone.0285354)

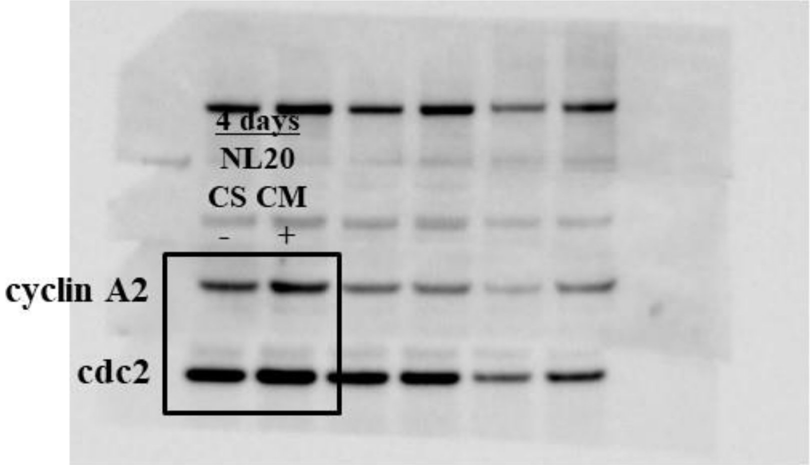

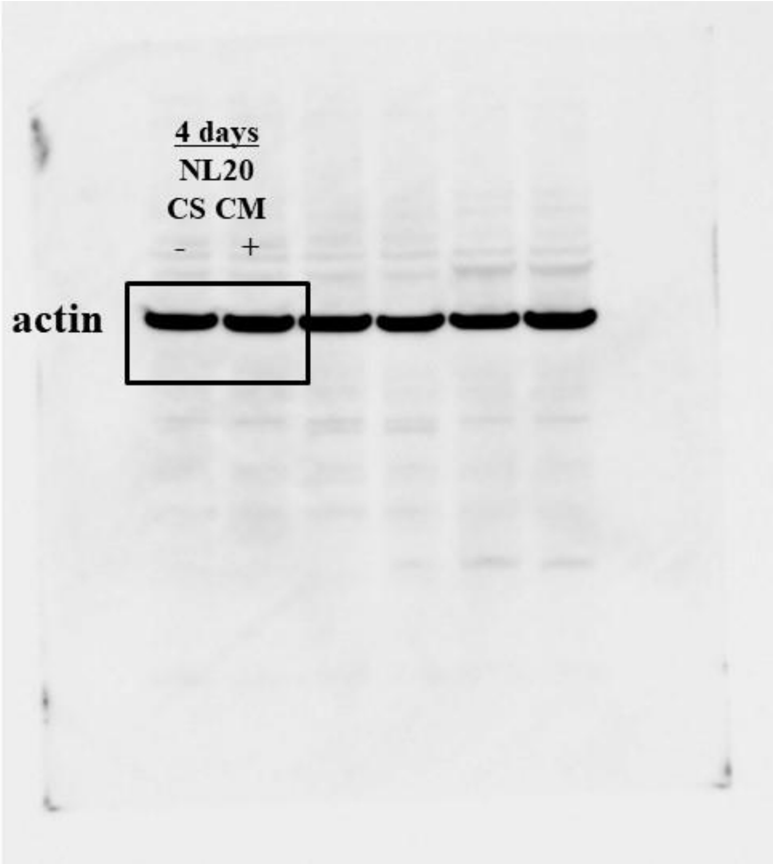

Fig 3 line 1 column 1

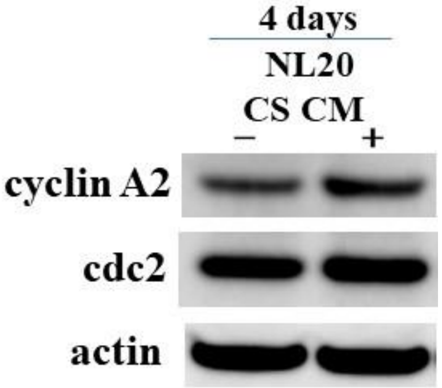

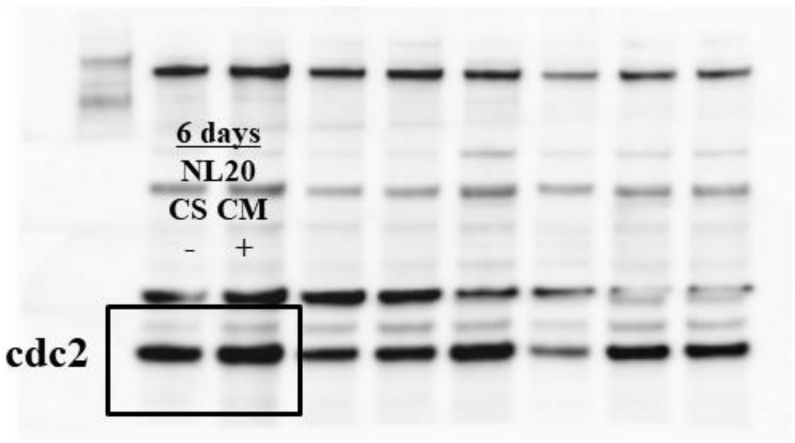

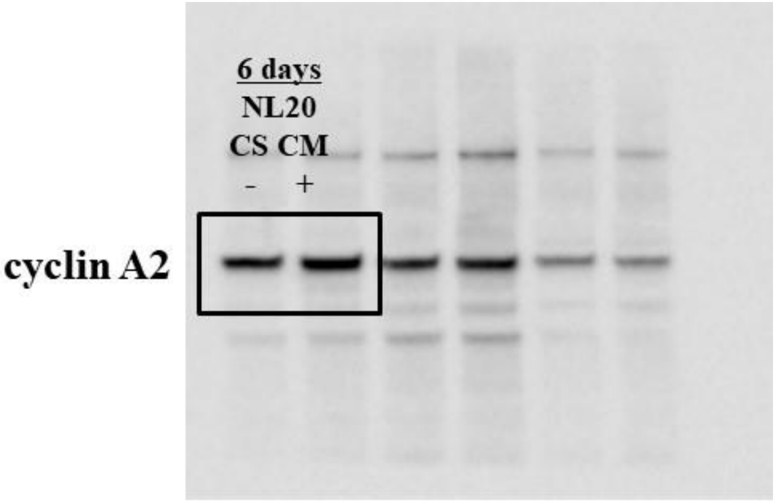

Fig 3 line 1 column 2

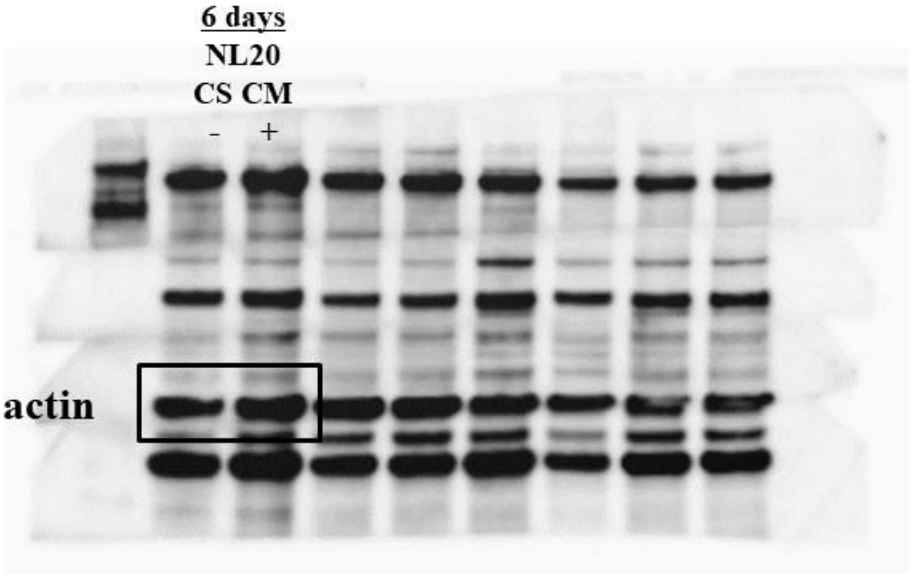

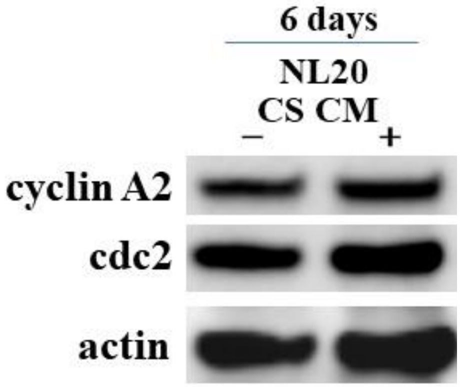

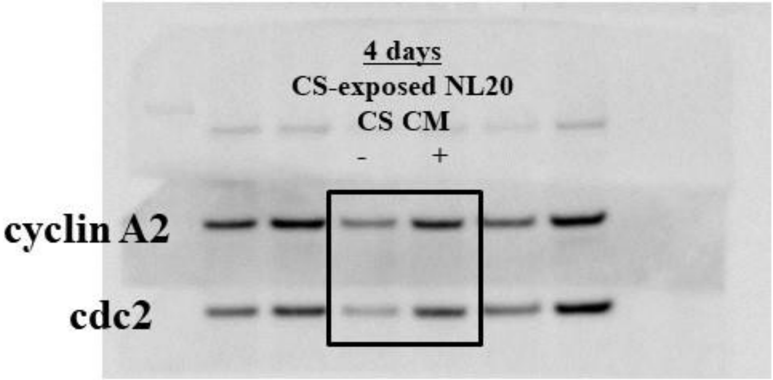

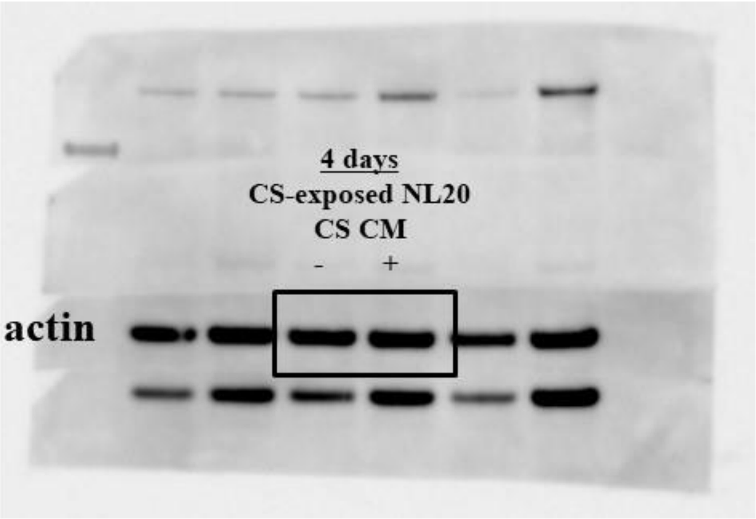

Fig 3 line 1 column 3

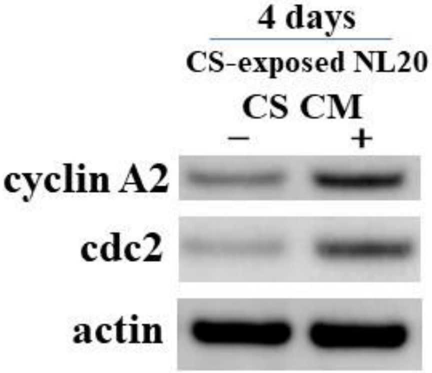

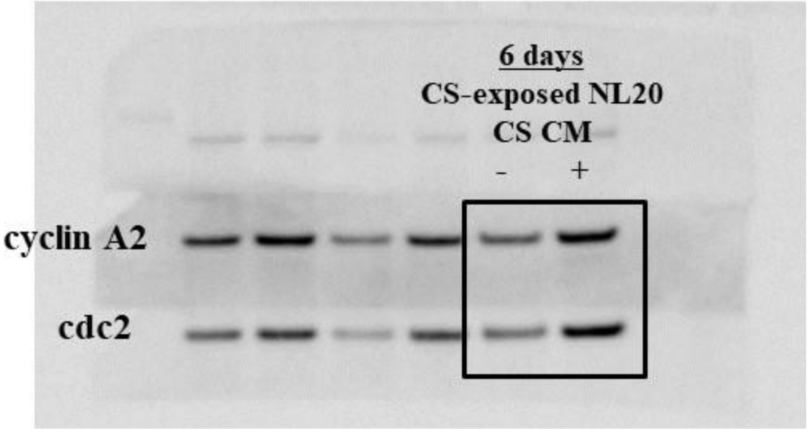

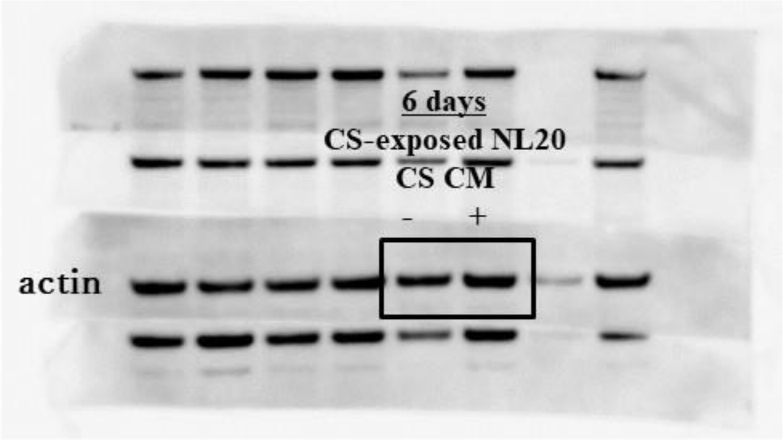

Fig 3 line 1 column 4

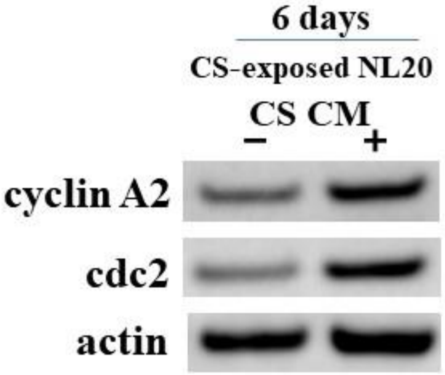

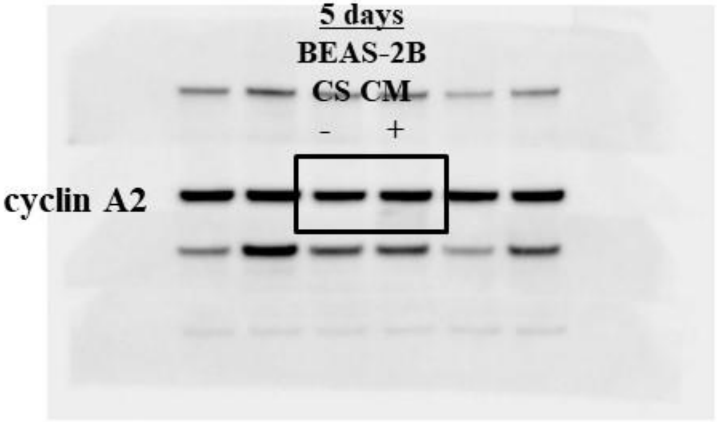

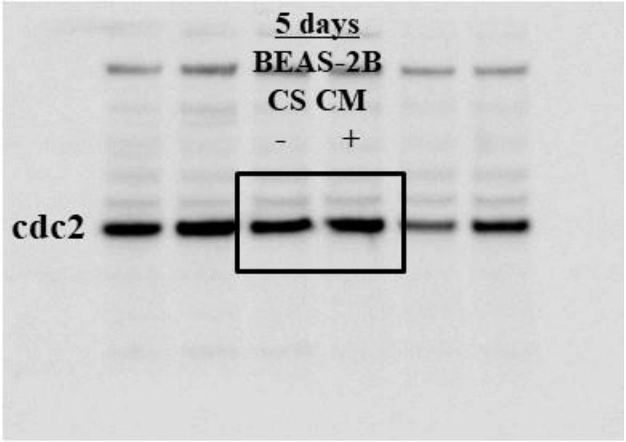

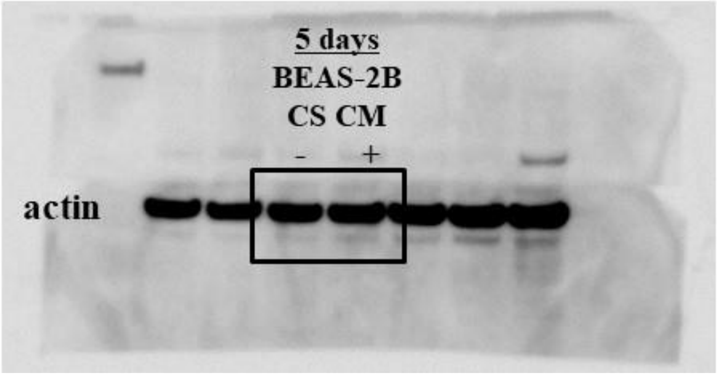

Fig 3 line 2 column 1

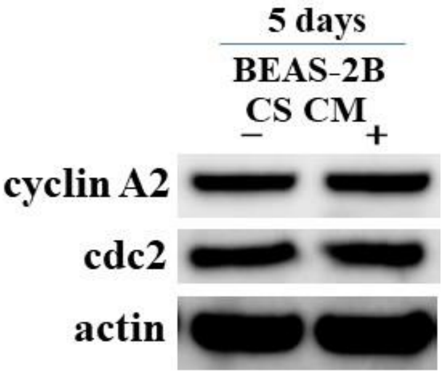

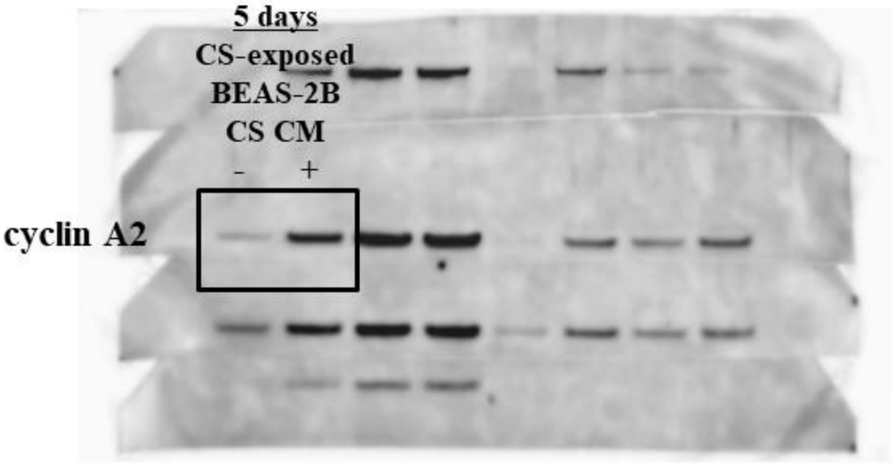

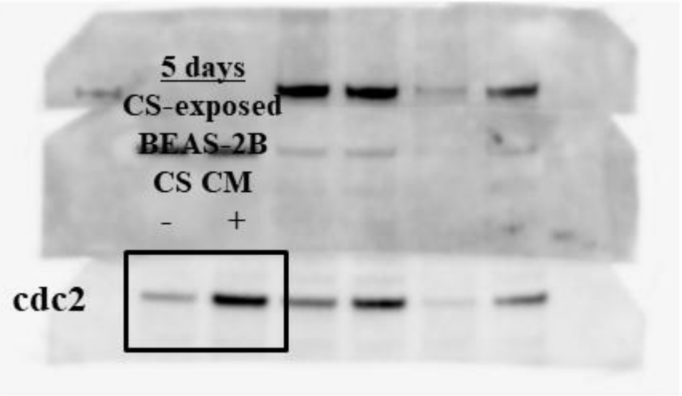

Fig 3 line 2 column 2

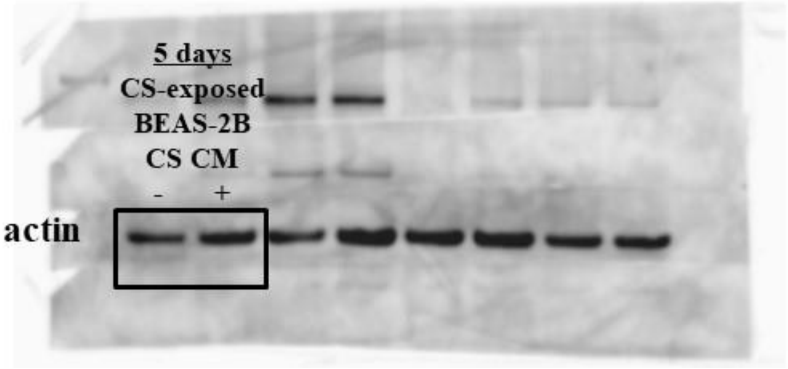

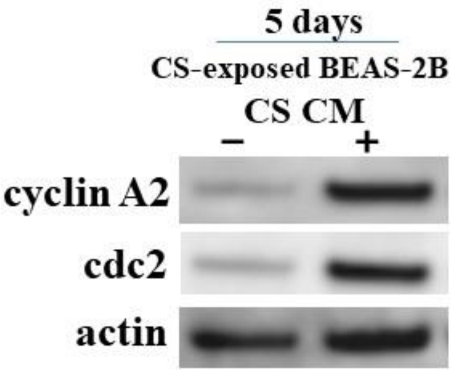

Fig 3 line 2 column 3

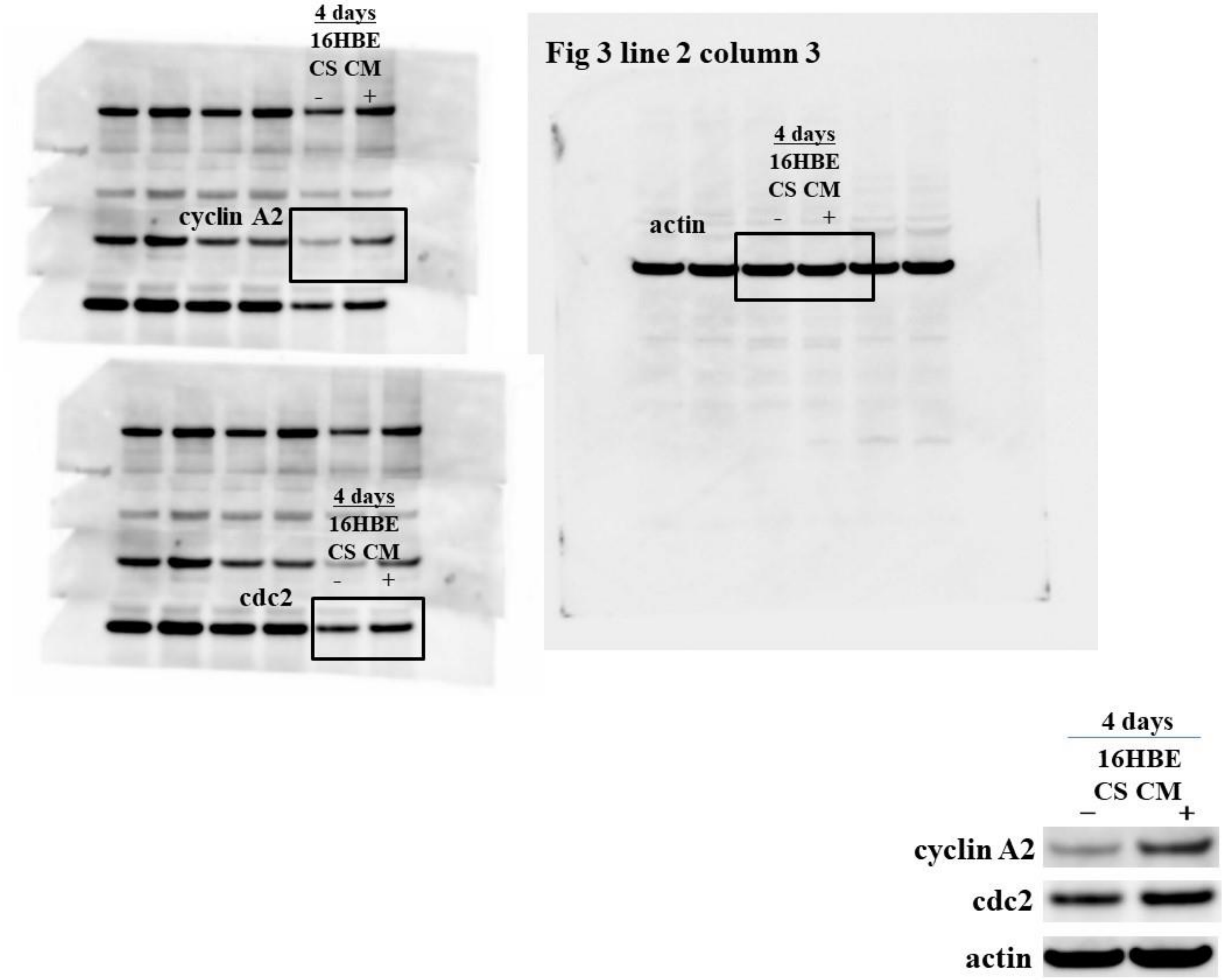

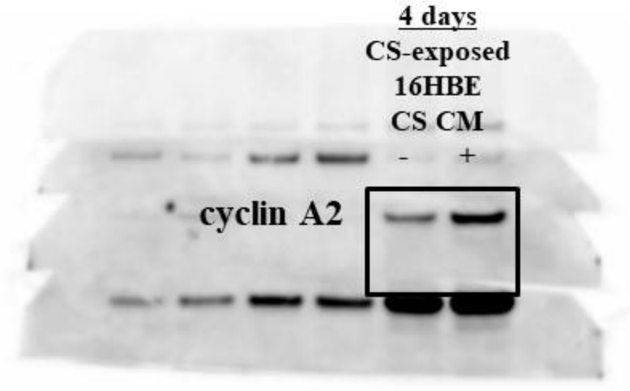

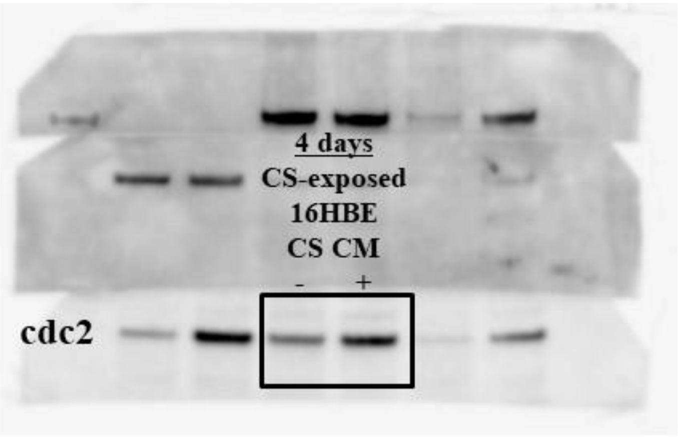

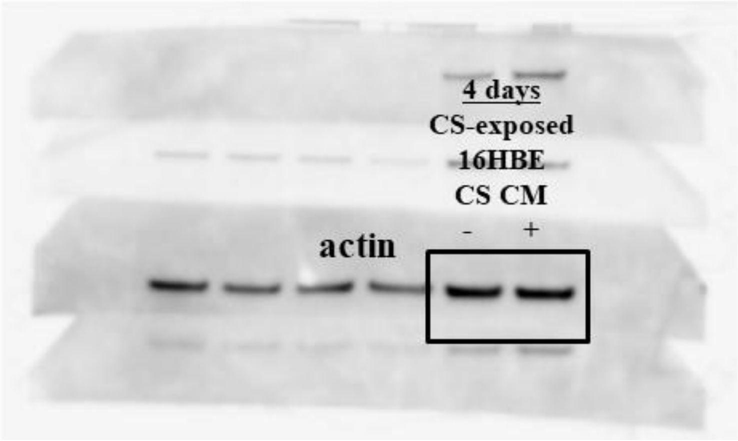

Fig 3 line 2 column 4

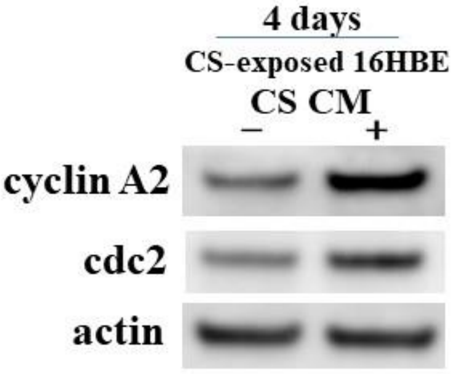

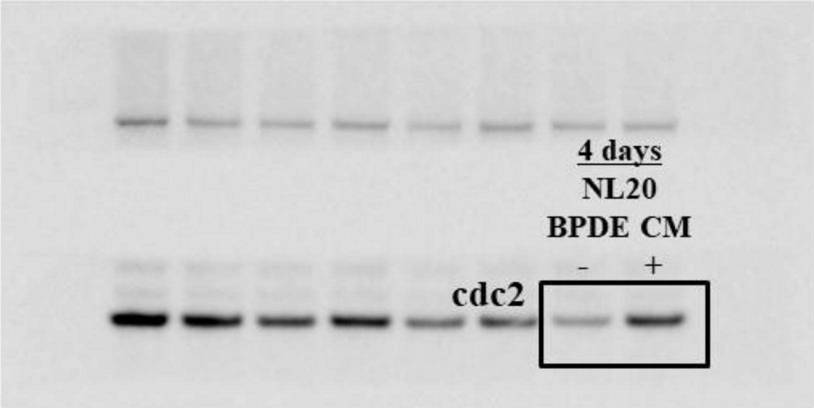

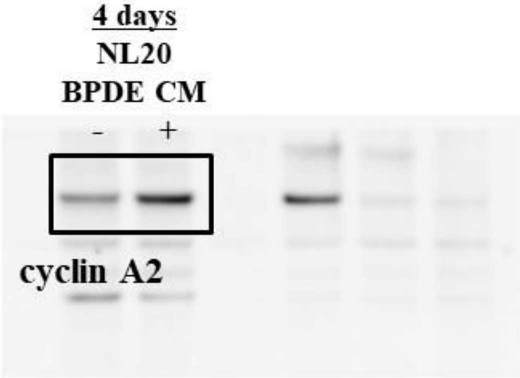

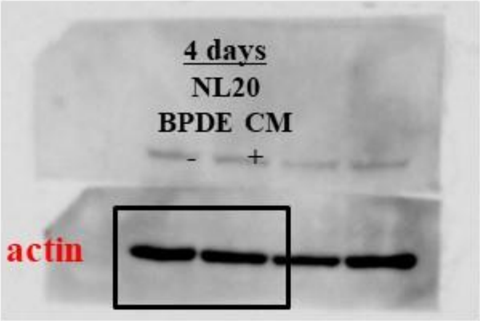

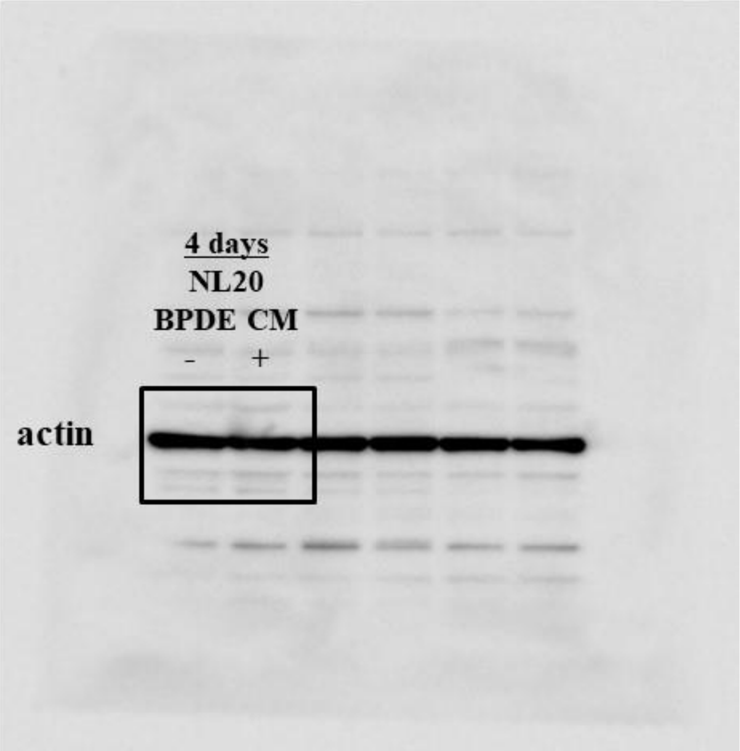

Fig 3 line 3 column 1

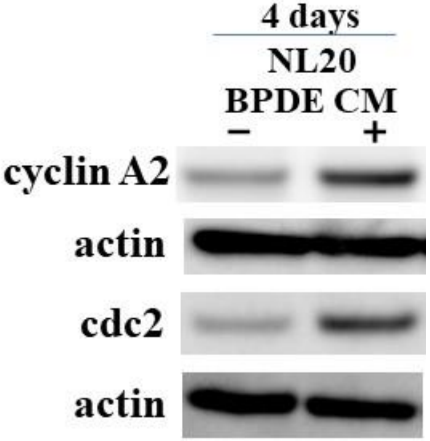

Fig 3 line 3 column 2

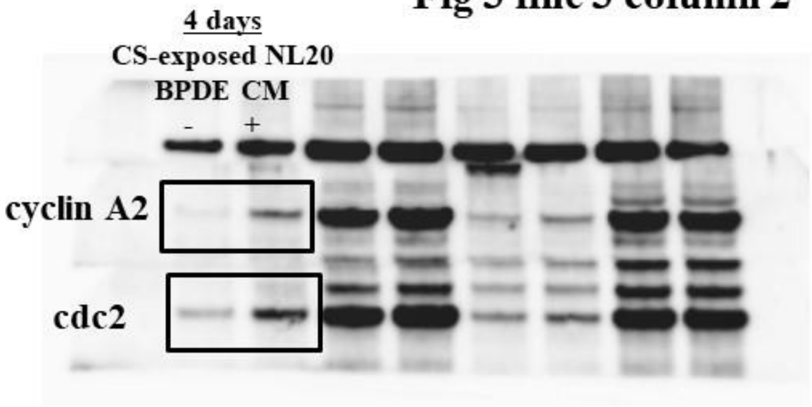

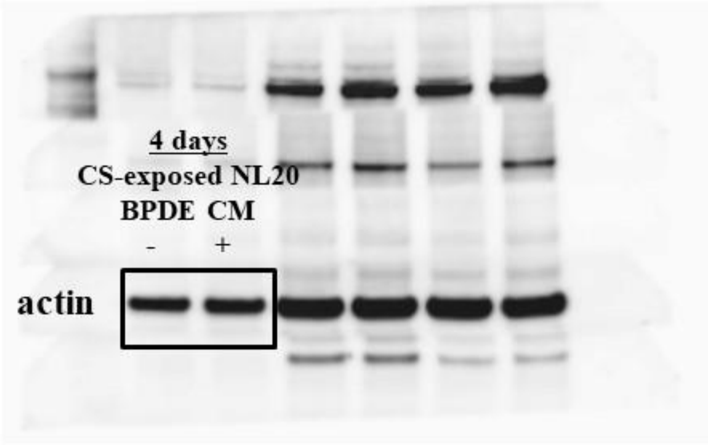

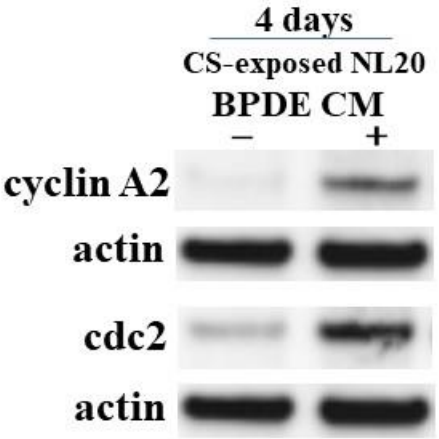

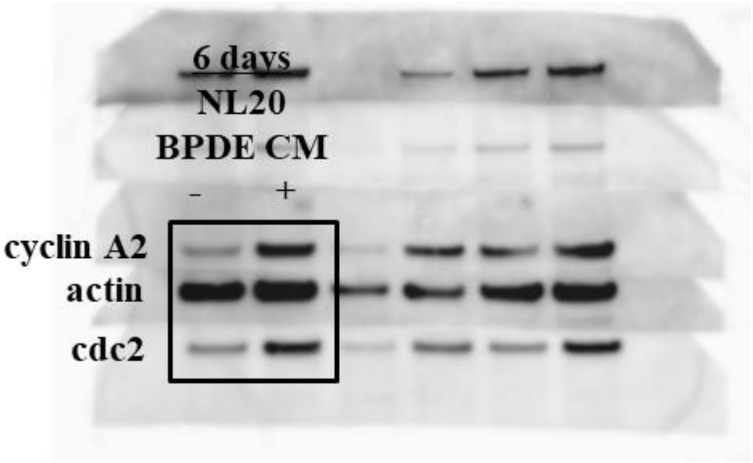

Fig 3 line 3 column 3

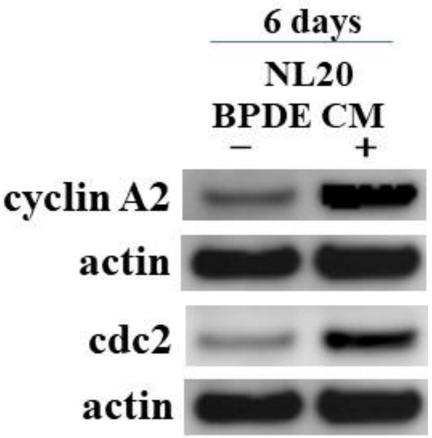

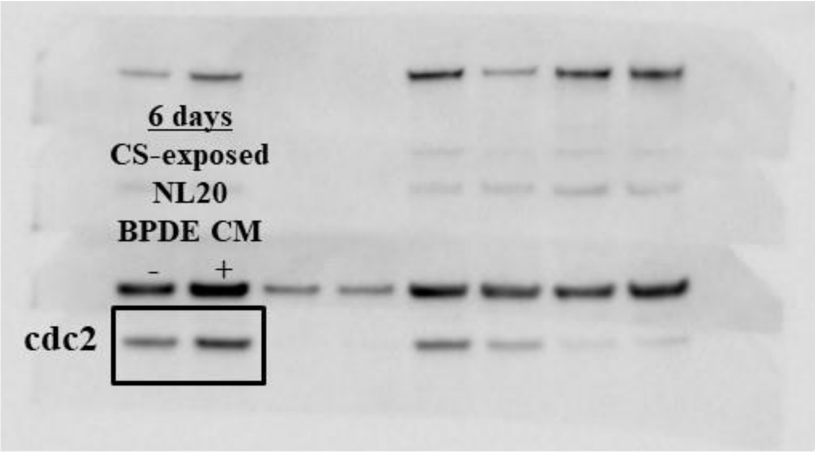

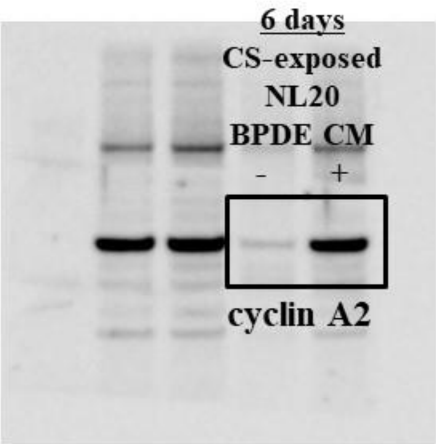

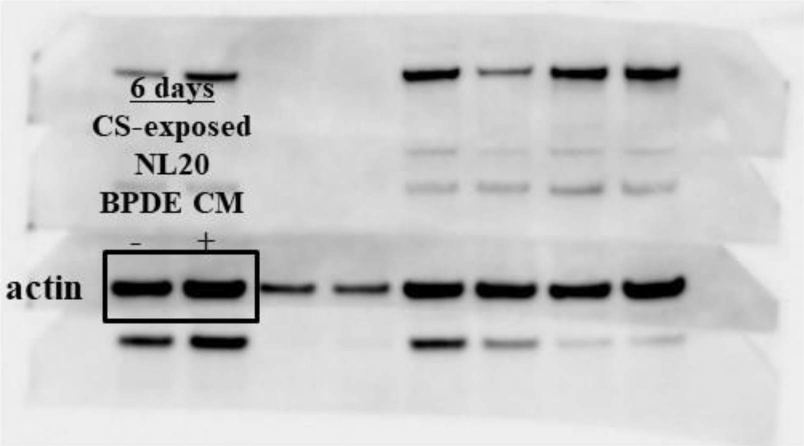

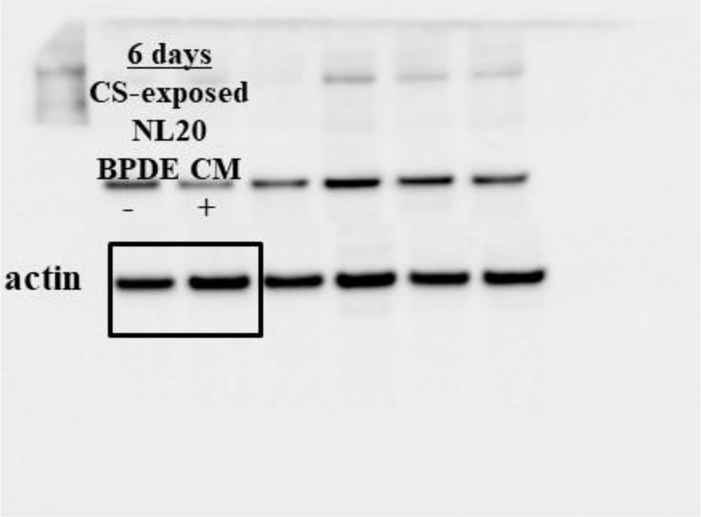

Fig 3 line 3 column 4

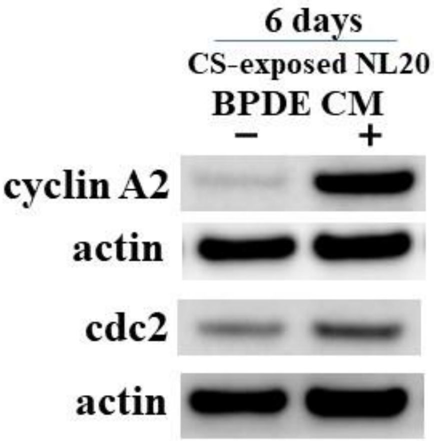

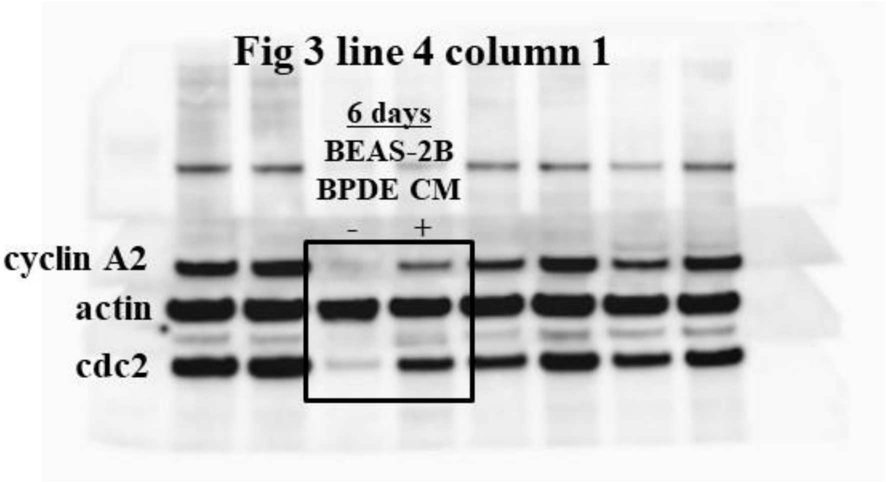
Fig 3 line 4 column 1
6 days
BEAS-2B
BPDE CM
- +
cyclin A2
actin
cdc2

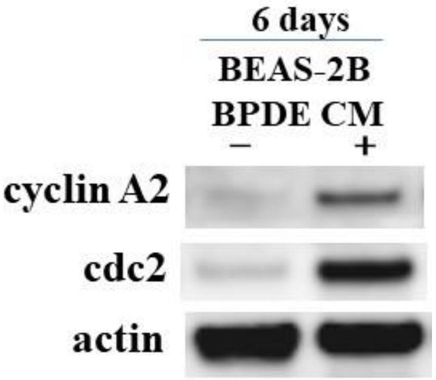
6 days
BEAS-2B
BPDE CM
− +
cyclin A2
cdc2
actin

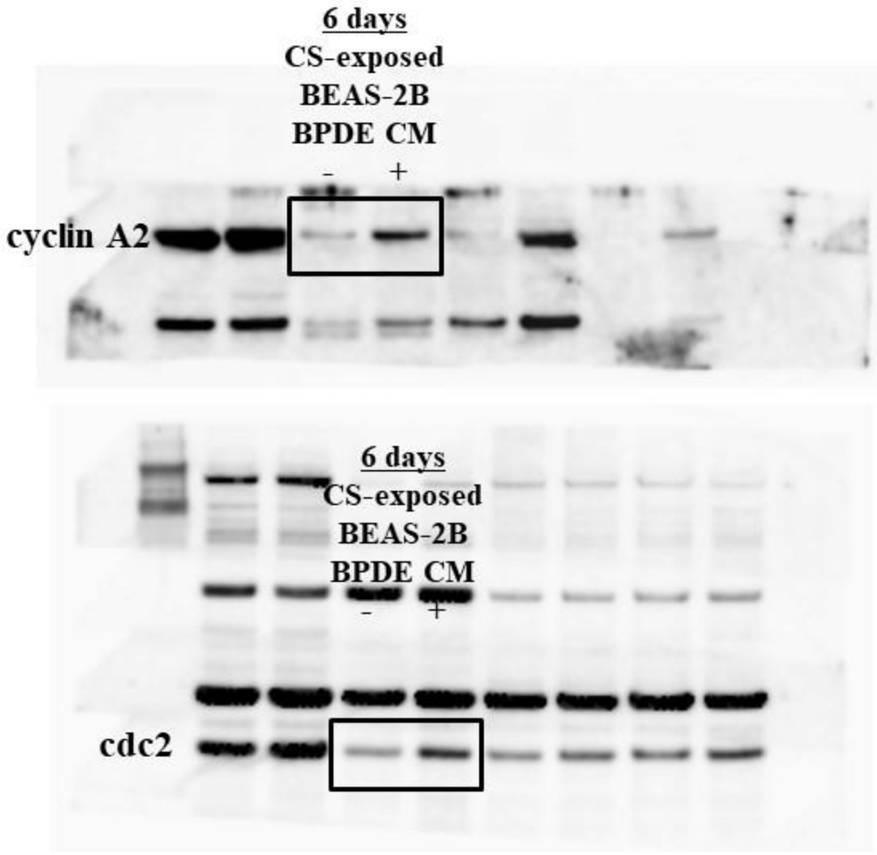

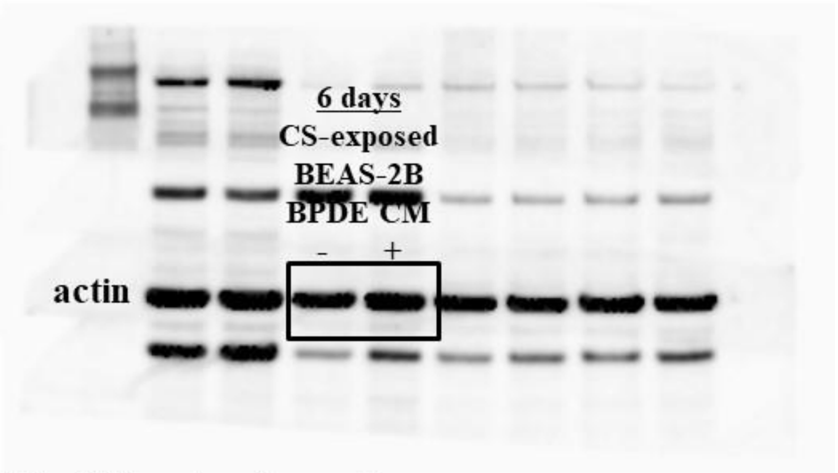

Fig 3 line 4 column 2

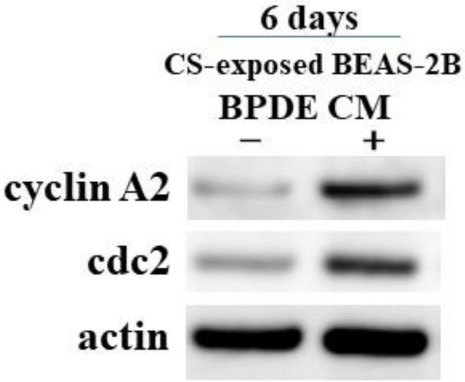

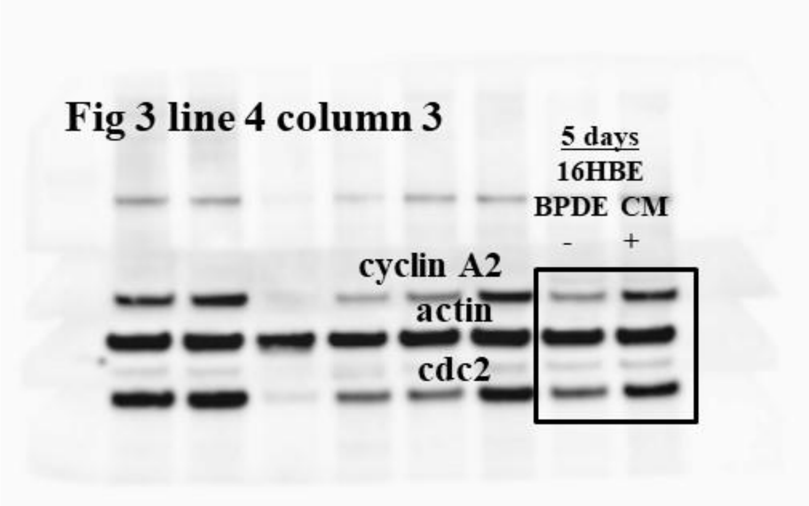
Fig 3 line 4 column 3
5 days
16HBE
BPDE CM
-
+
cyclin A2
actin
cdc2

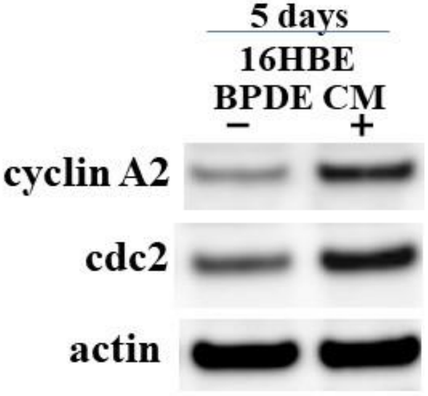
5 days
16HBE
BPDE CM
−
+
cyclin A2
cdc2
actin

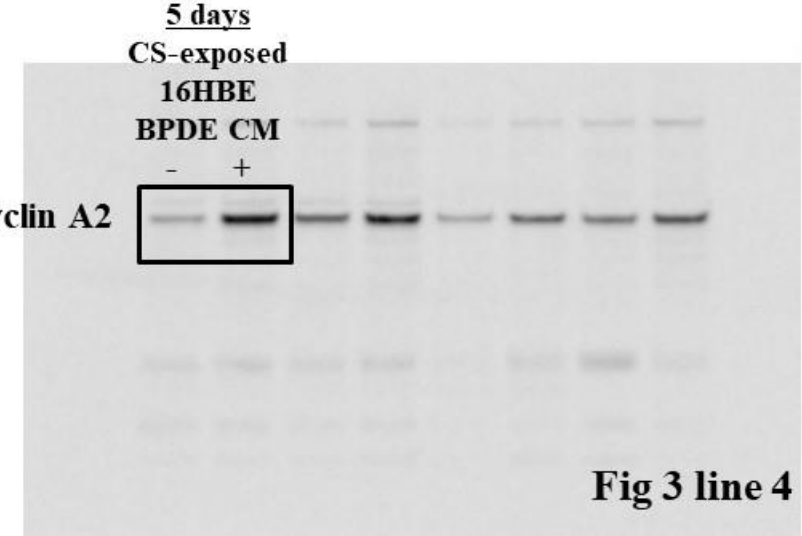

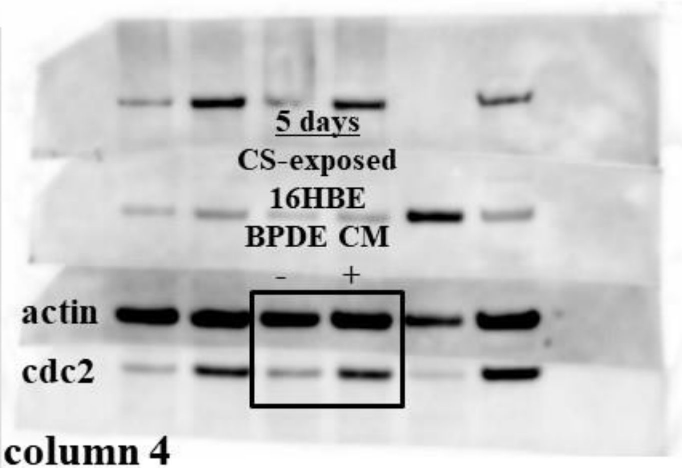

Fig 3 line 4 column 4

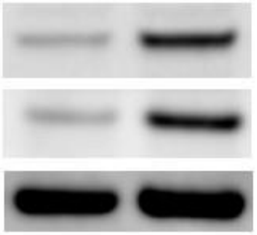

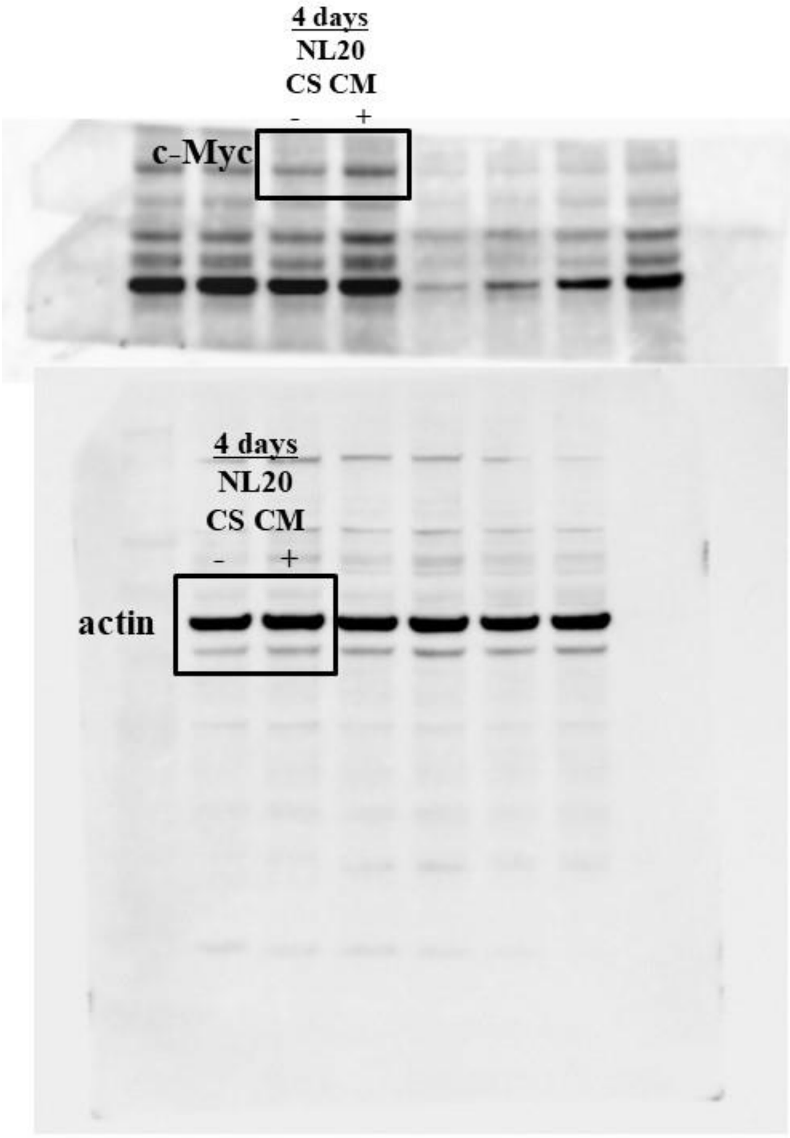

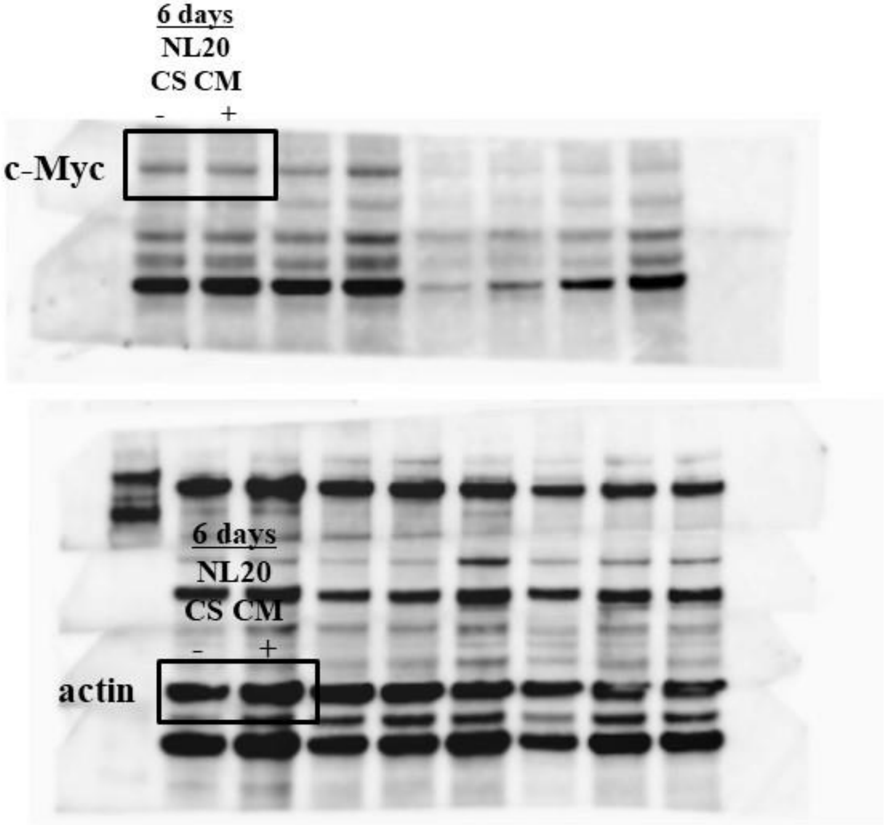

Fig 4A line 1 column 1

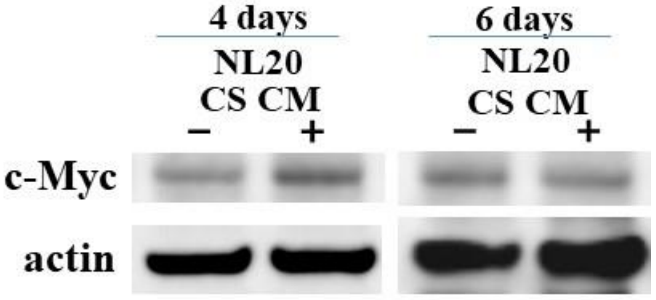

Fig 4A line 1 column 2

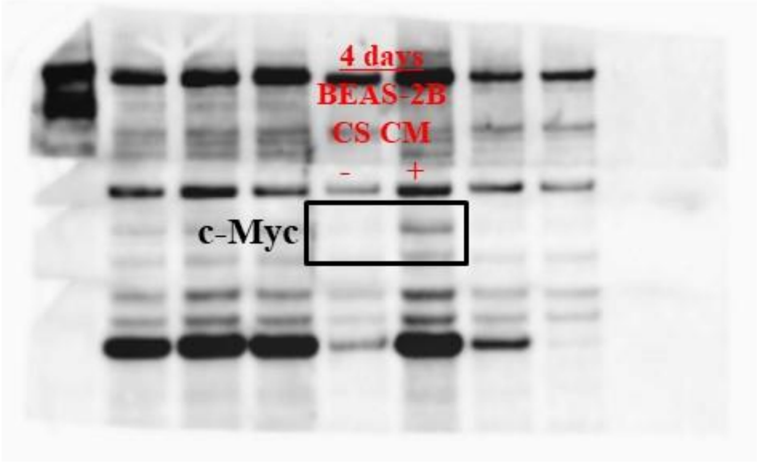

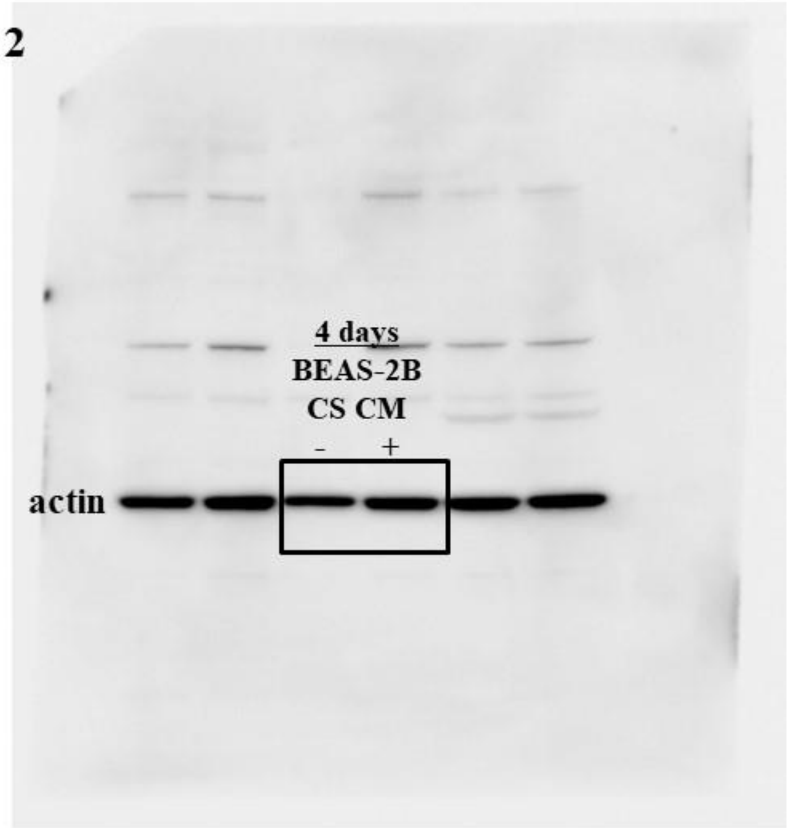

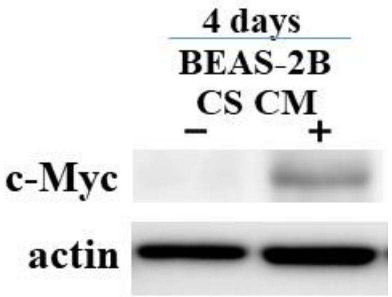

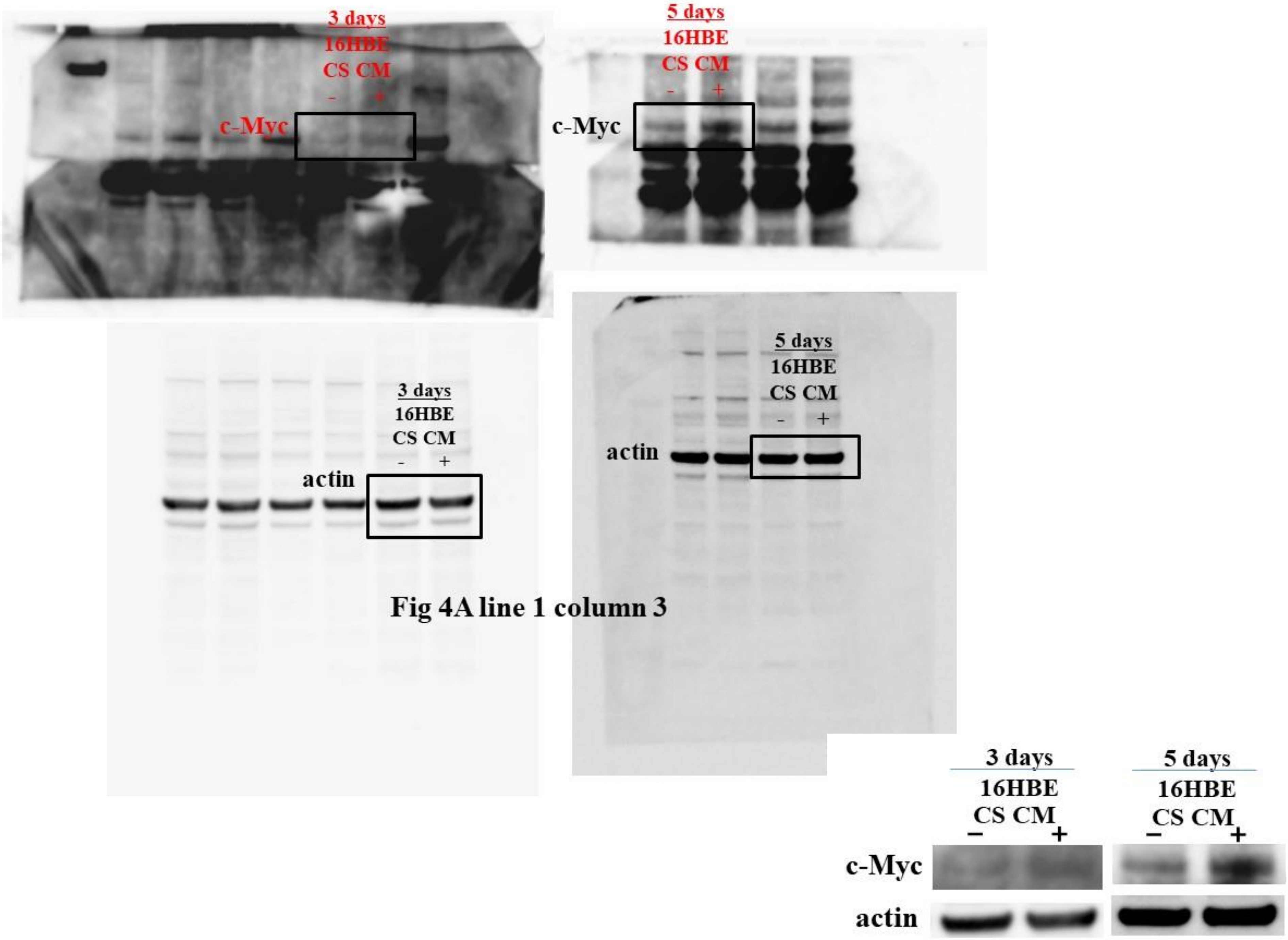

Fig 4A line 1 column 3

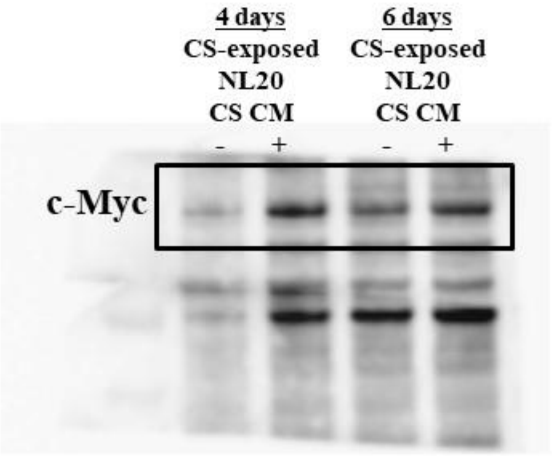

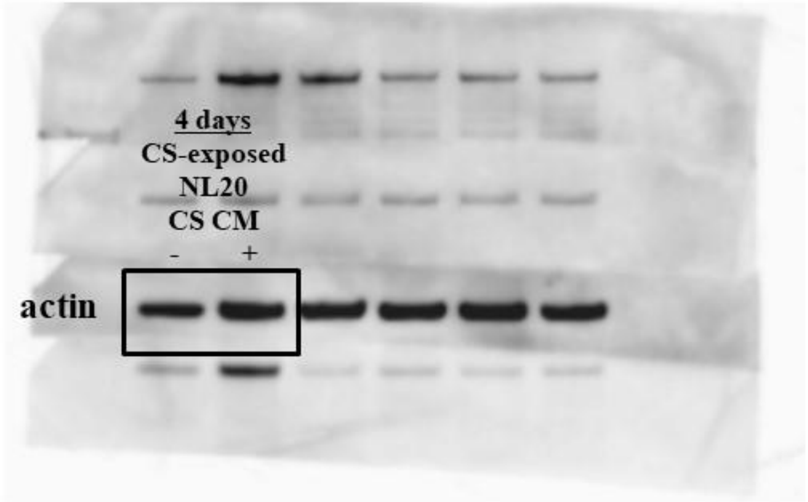

Fig 4A line 2 column 1

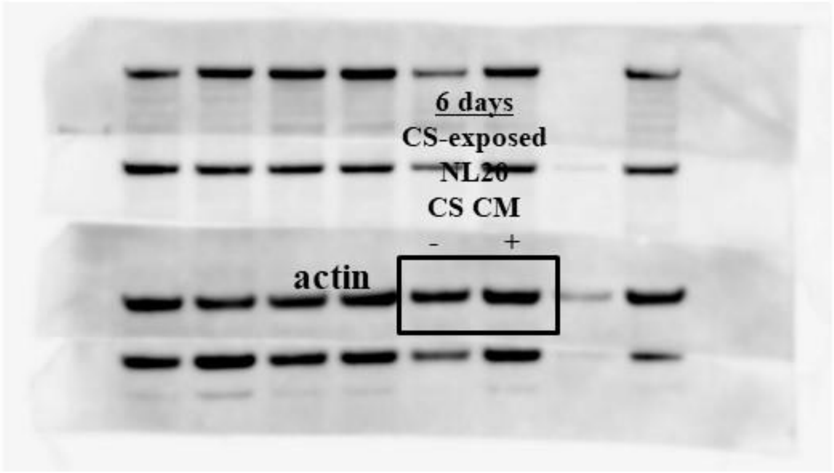

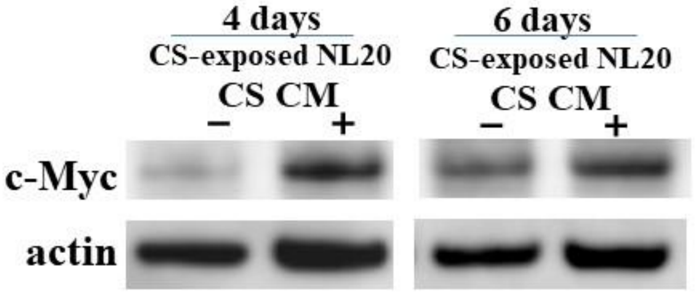

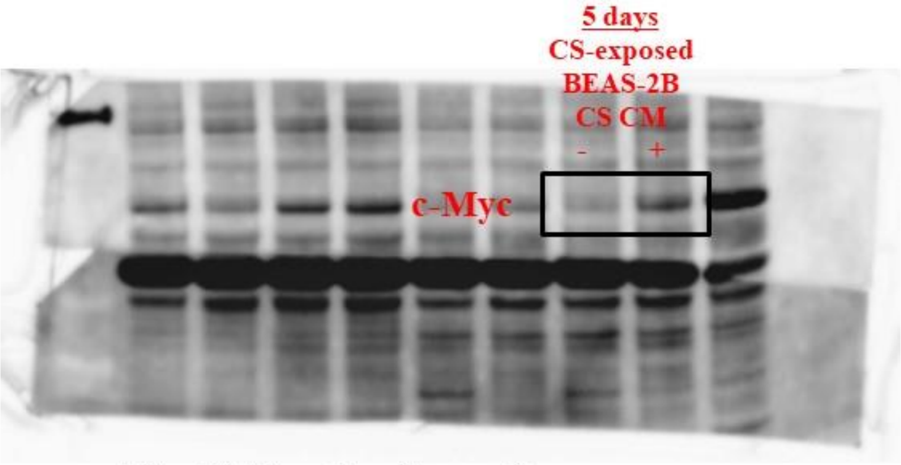

Fig 4A line 2 column 2

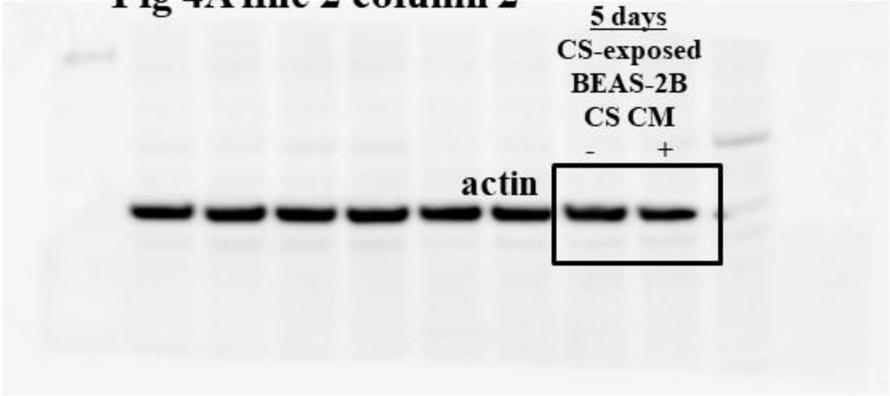

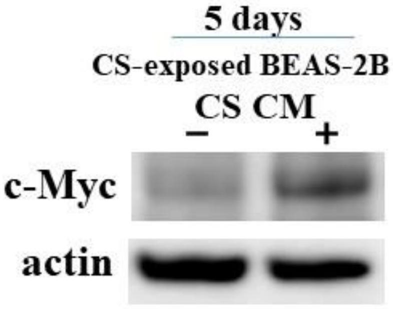

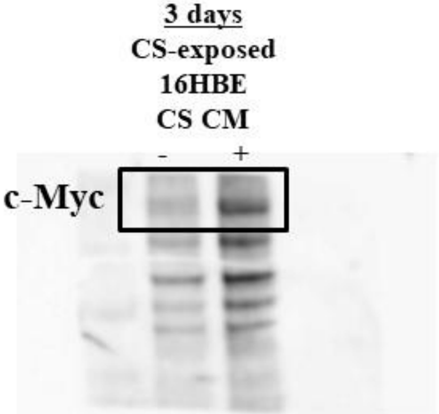

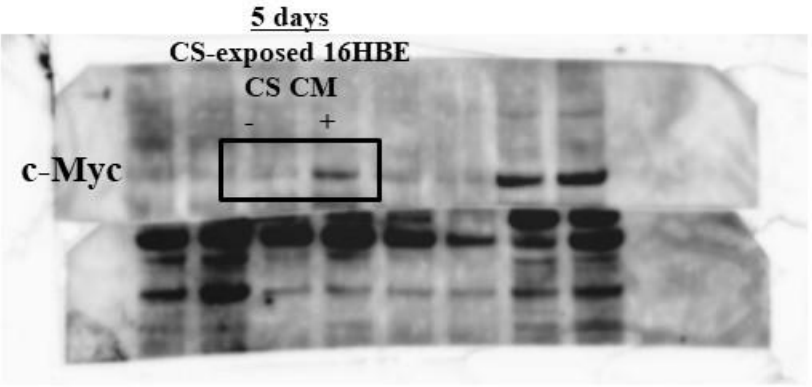

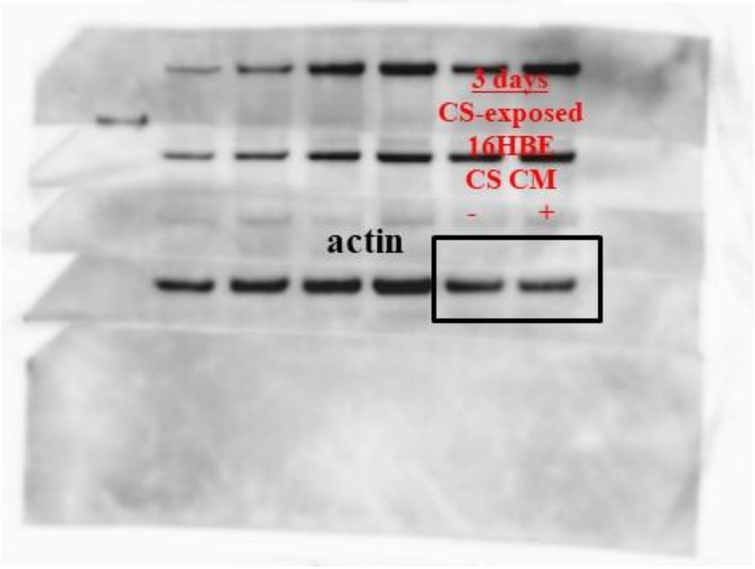

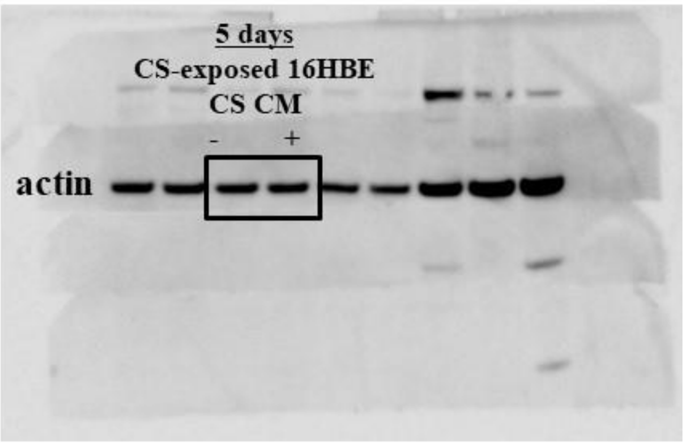

Fig 4A line 2 column 3

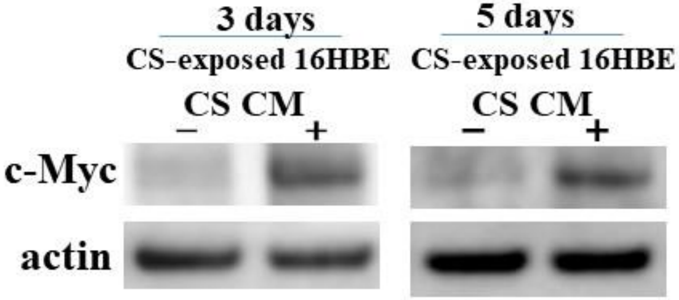

Fig 4A line 3 column 1

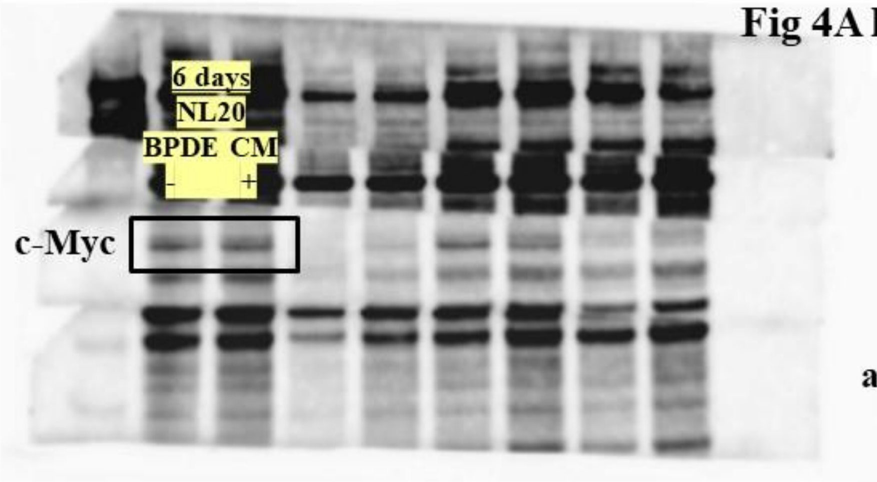

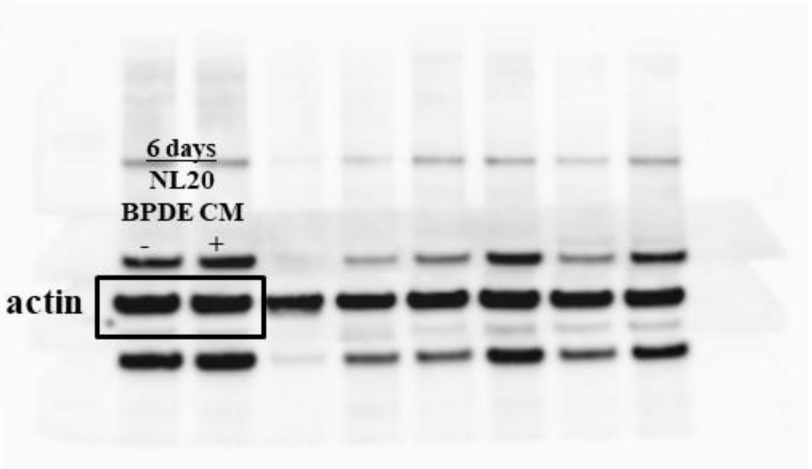

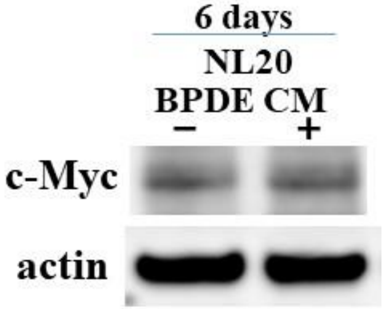

Fig 4A line 3 column 2

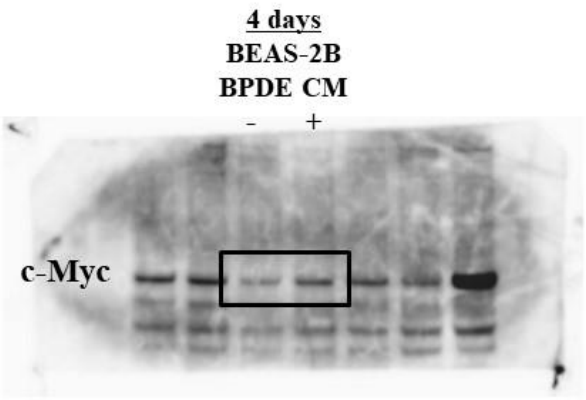

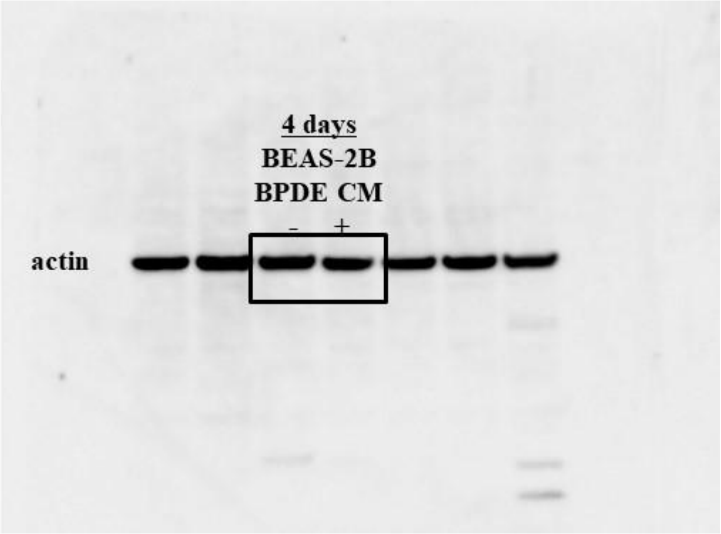

Fig 4A line 3 column 2

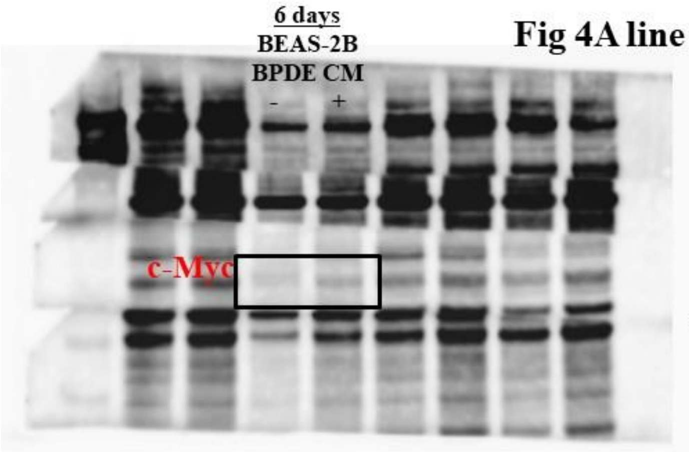

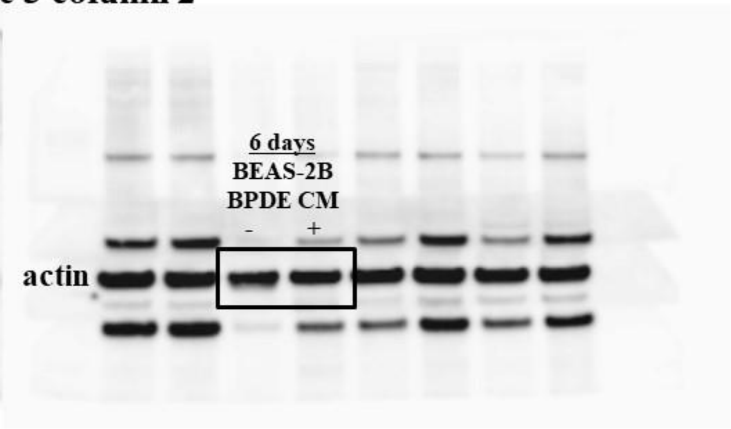

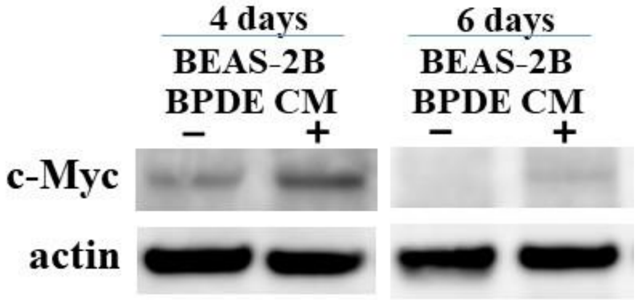

Fig 4A line 3 column 3

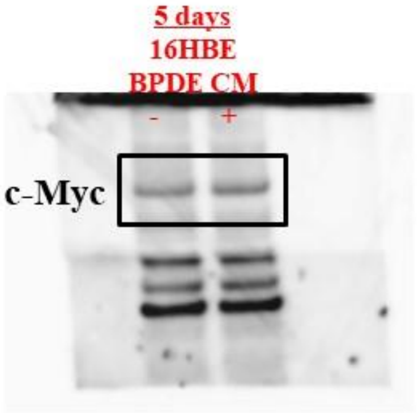

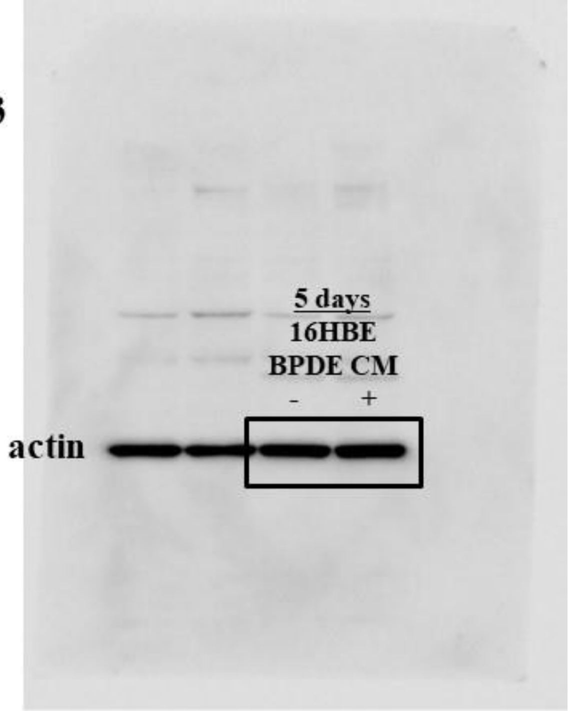

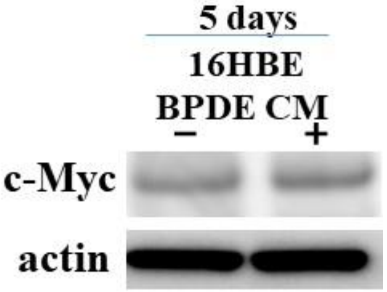

Fig 4A line 4 column 1

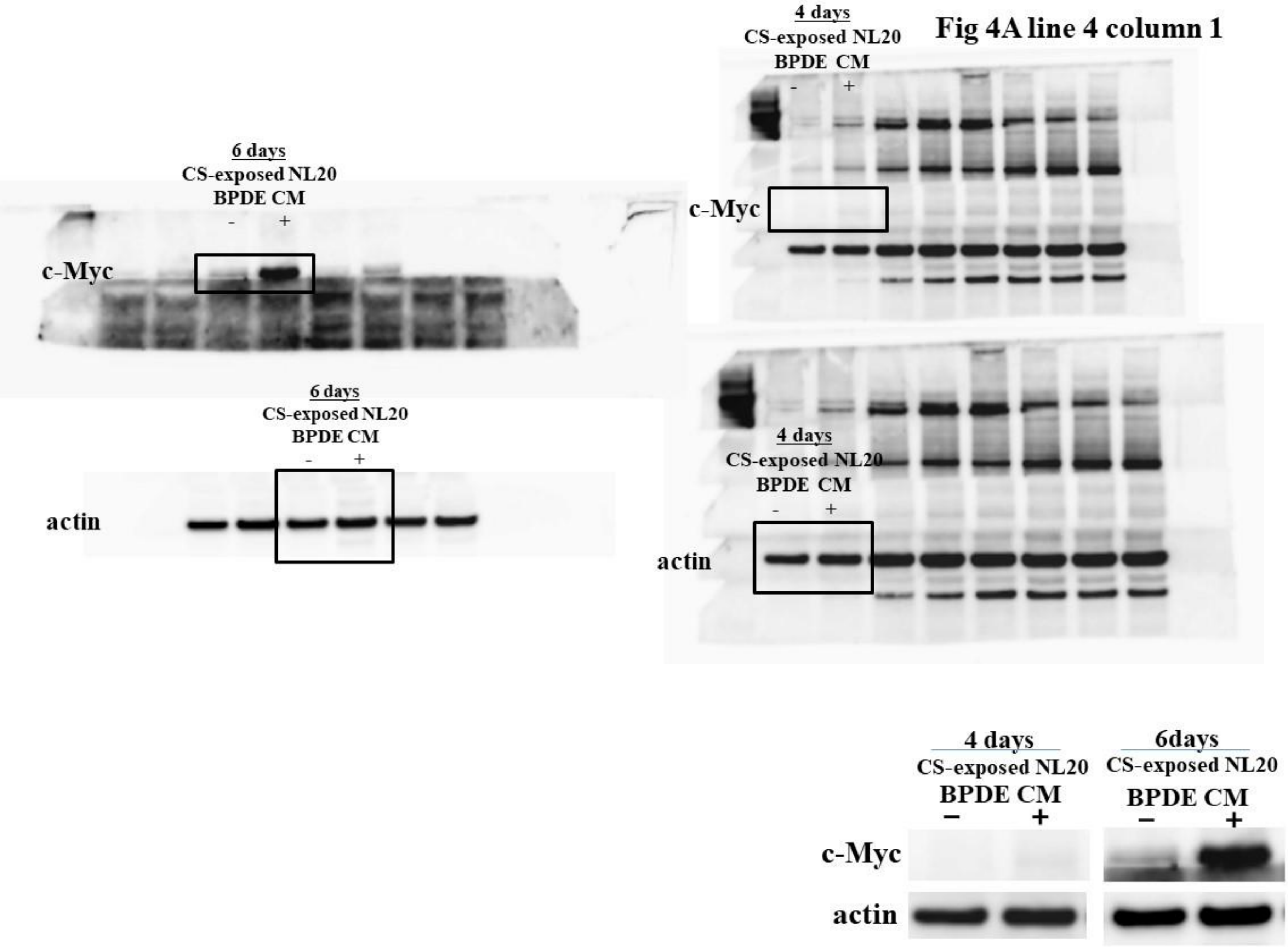

Fig 4A line 4 column 2

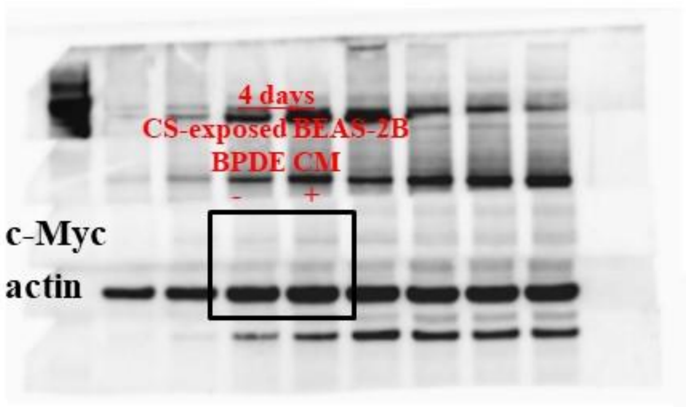

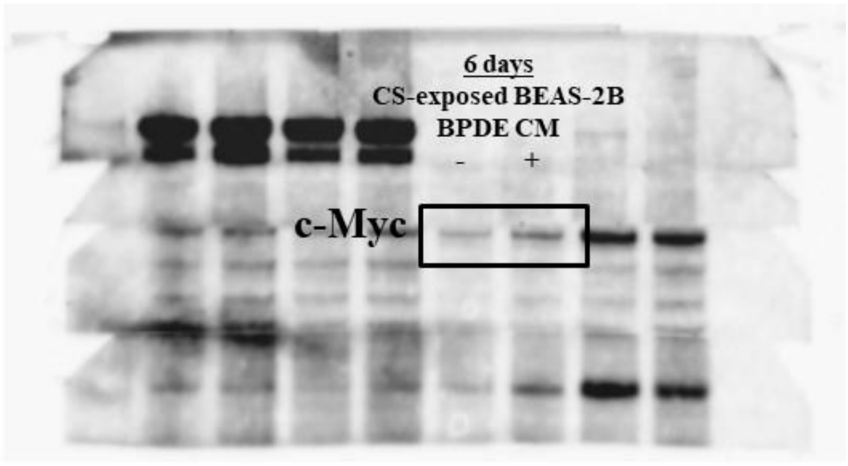

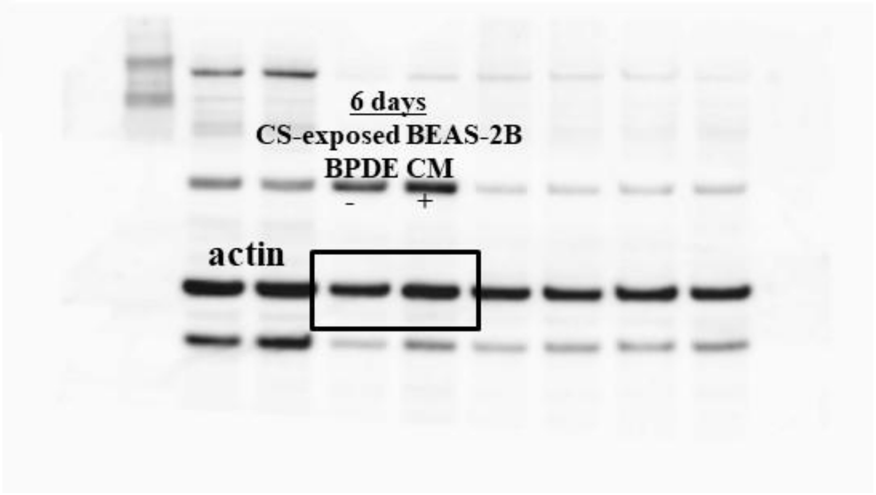

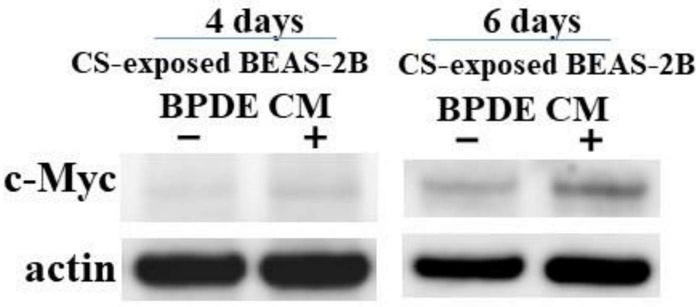

Fig 4A line 4 column 3

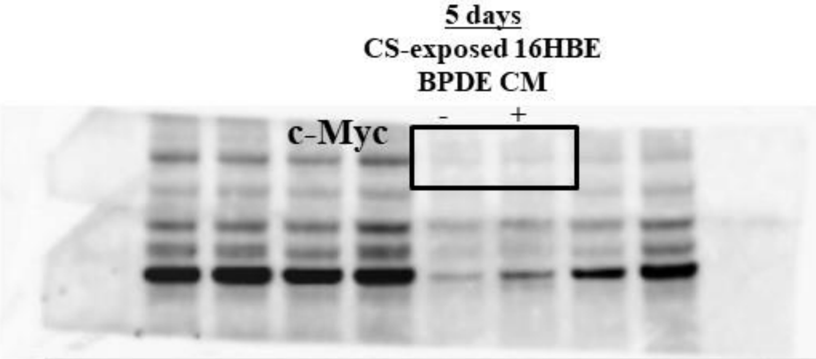

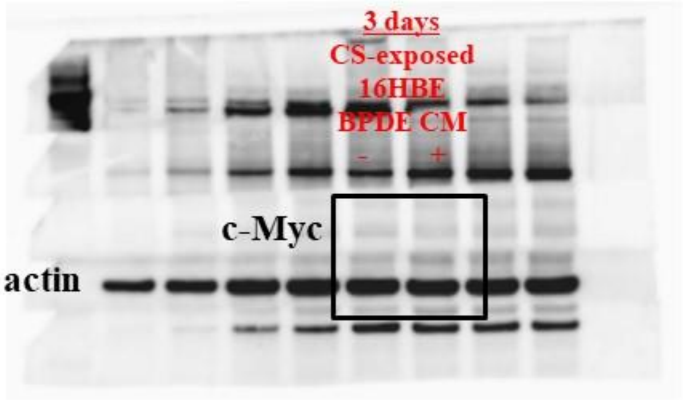

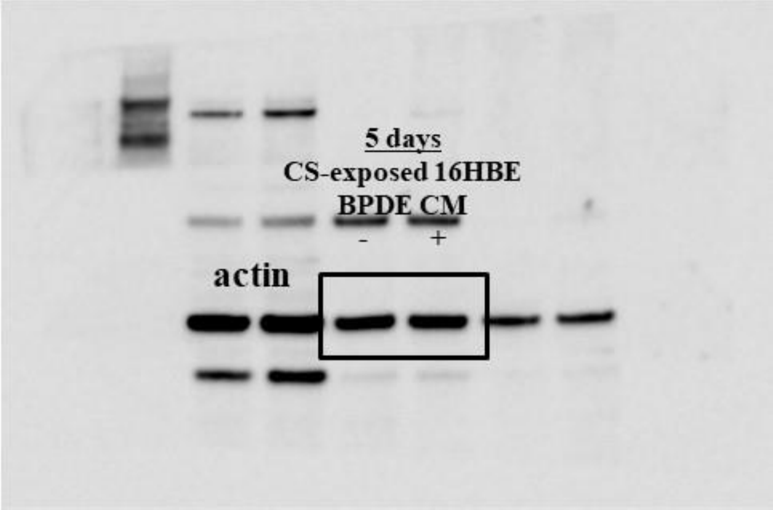

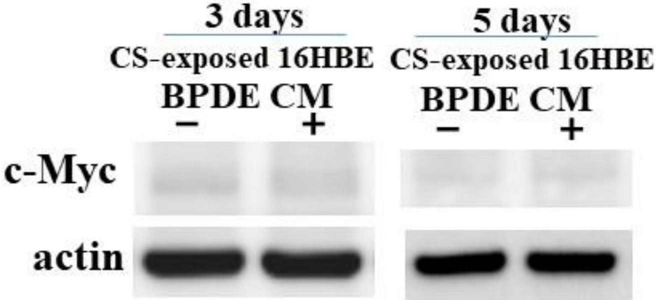

Fig 4B line 1 column 1

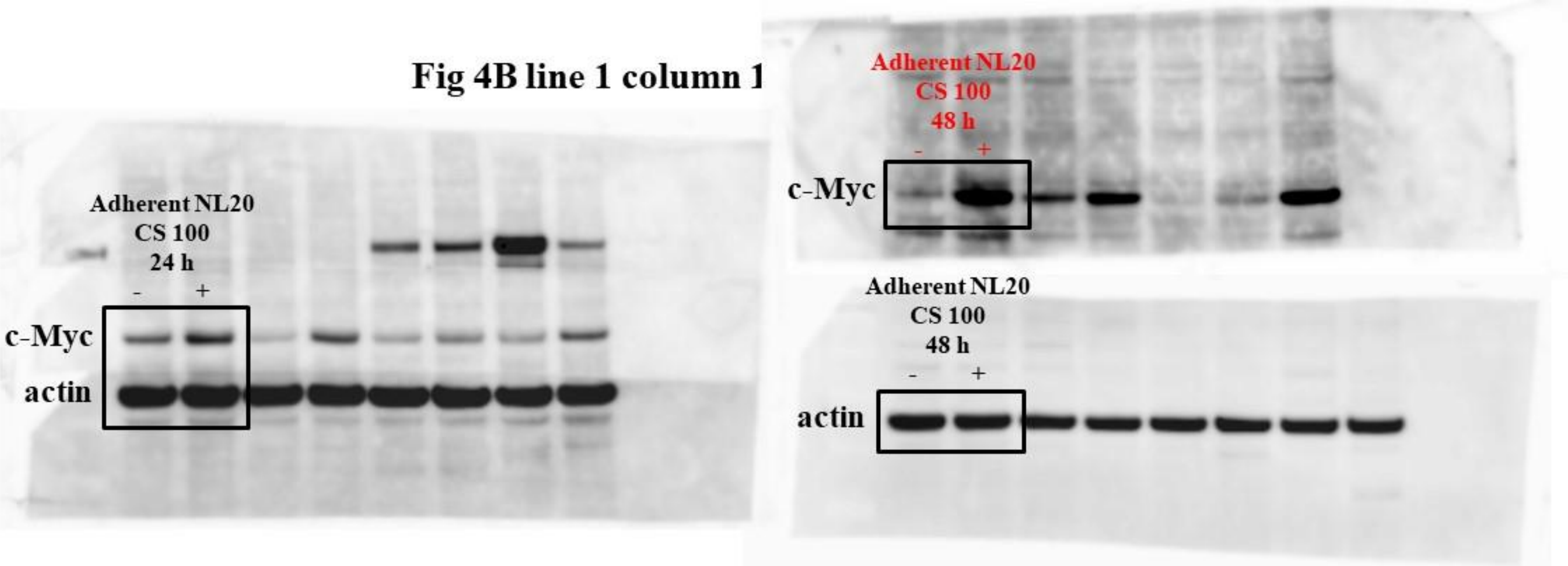

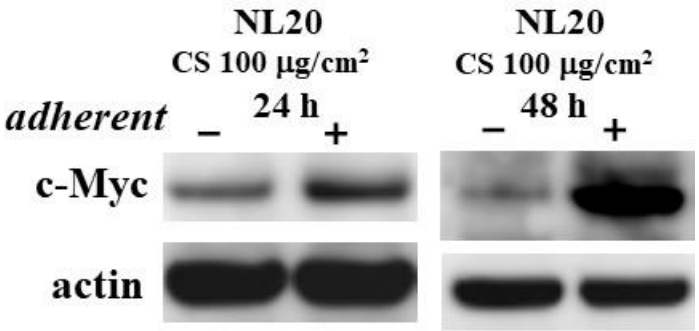

Fig 4B line 1 column 2

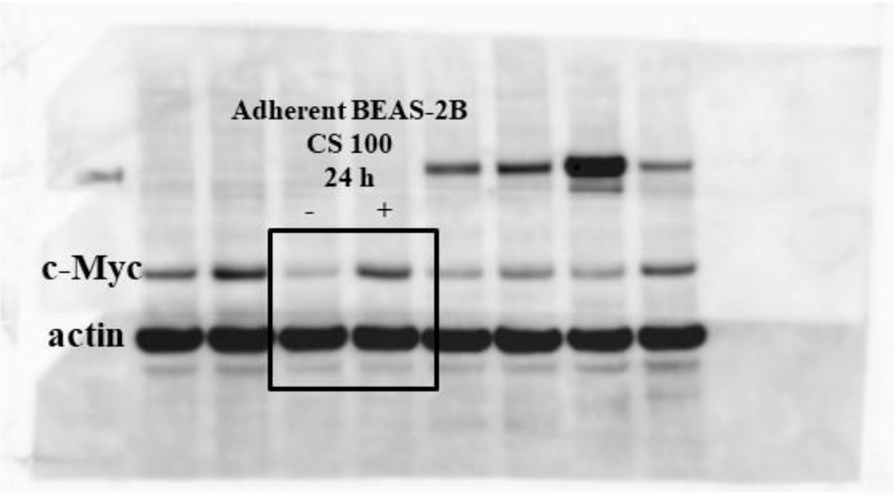

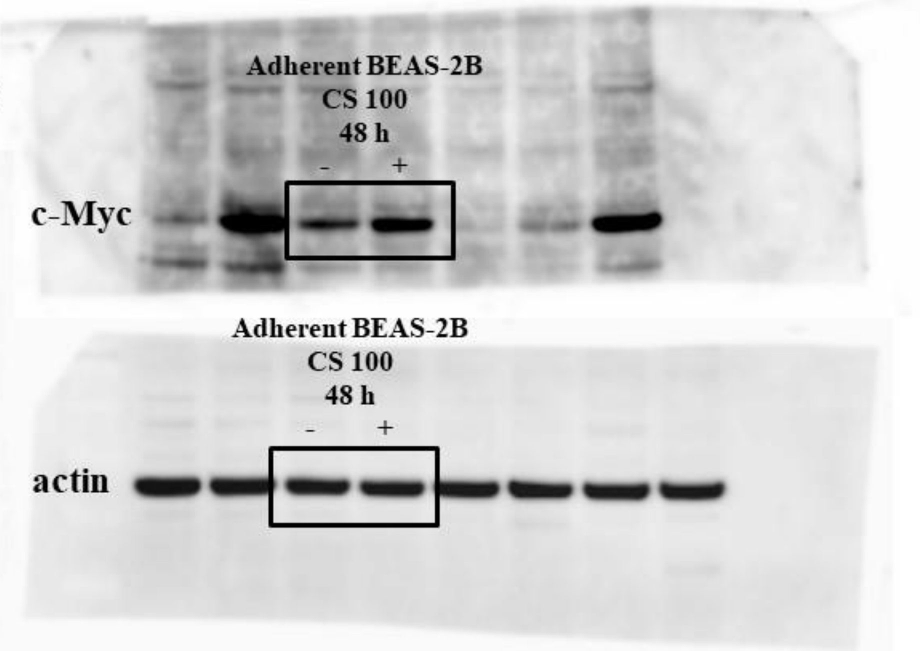

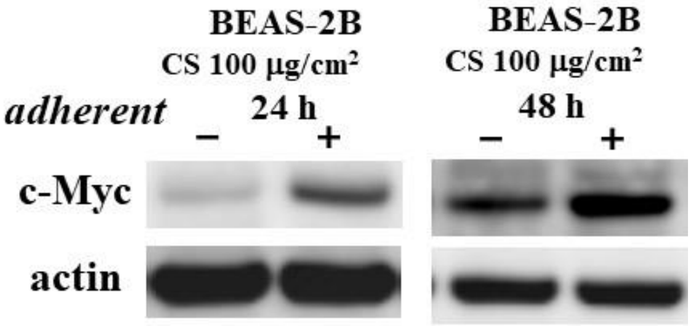

Fig 4B line 1 column 3

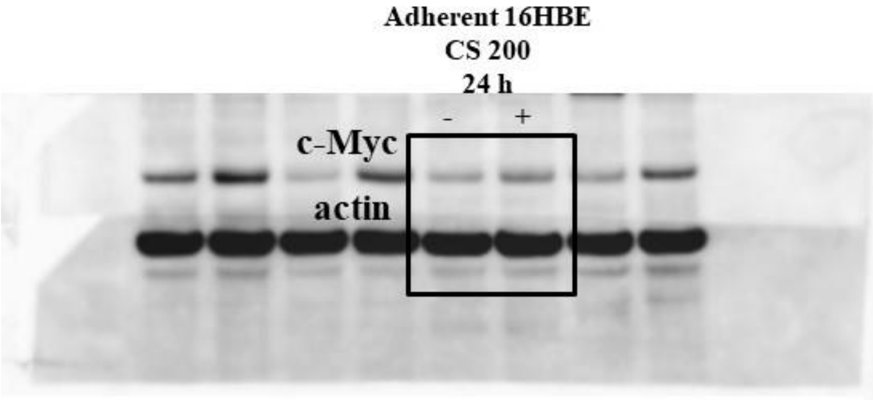

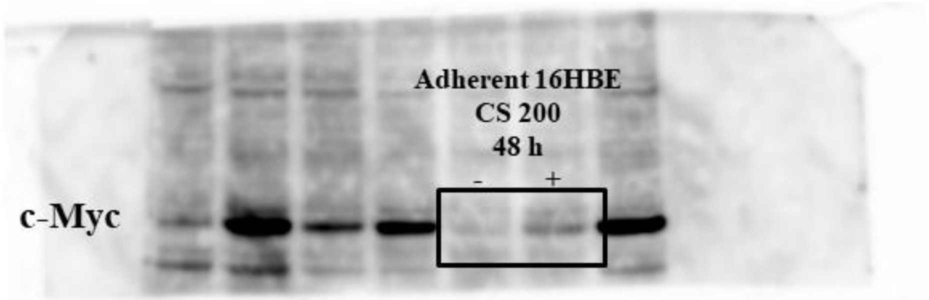

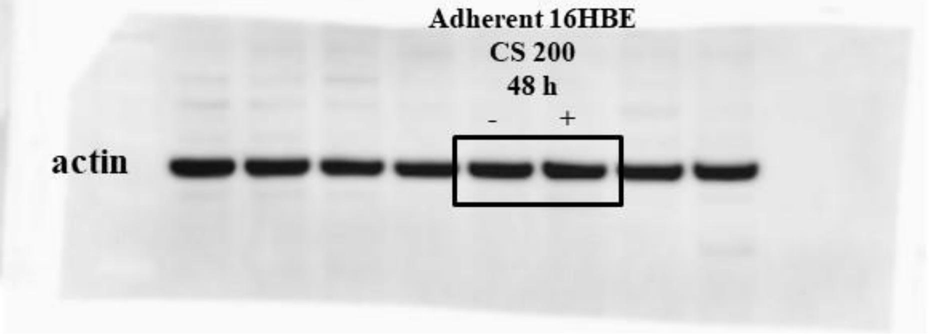

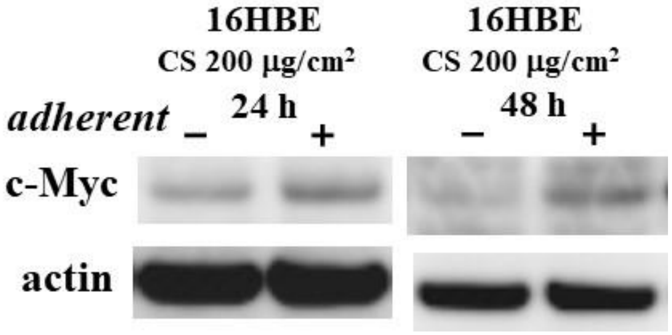

Fig 4B line 2 column 1

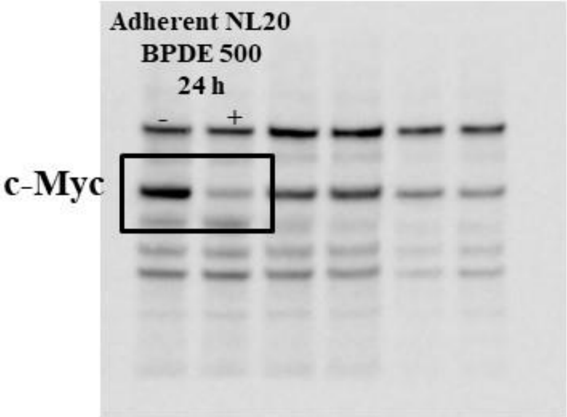

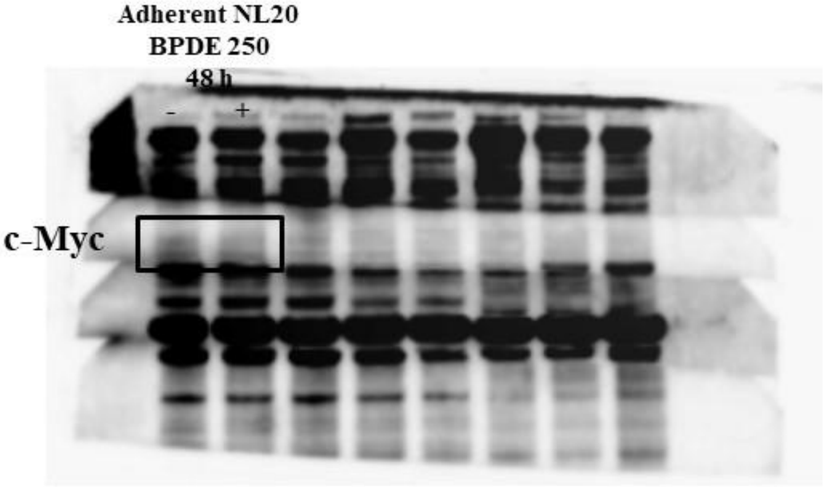

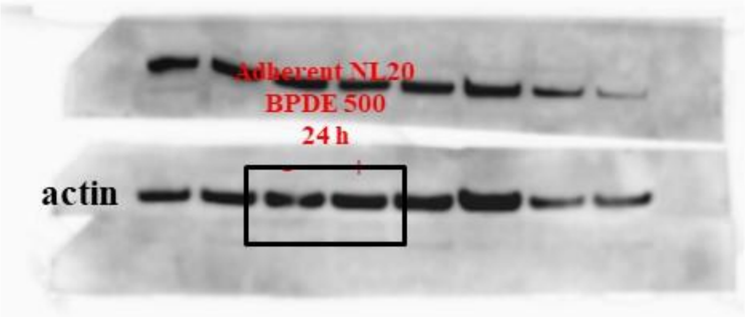

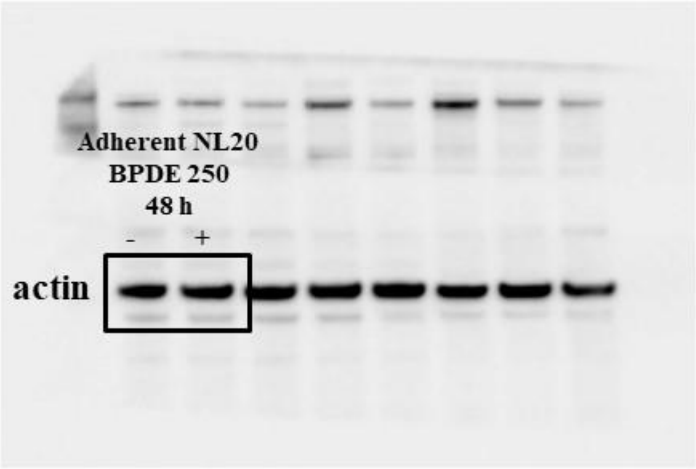

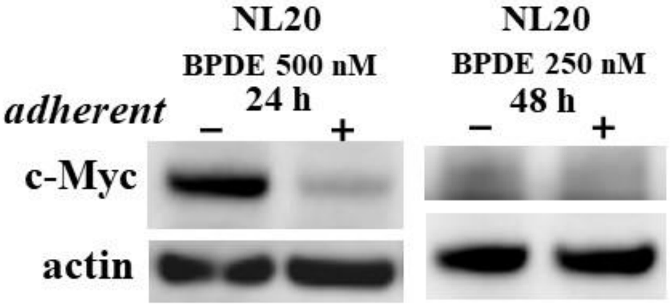

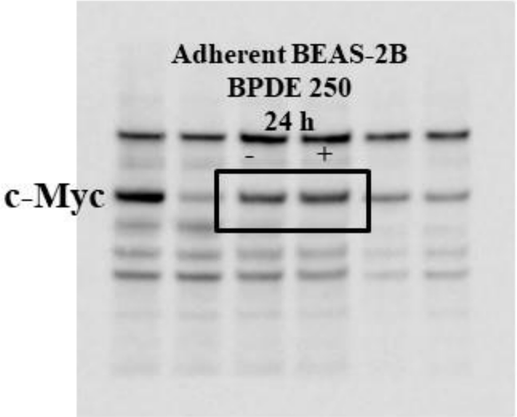

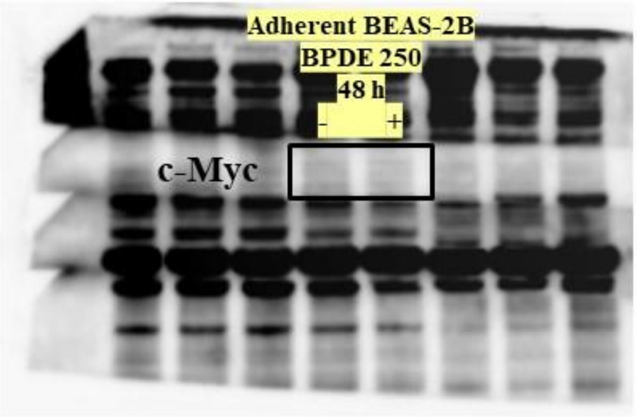

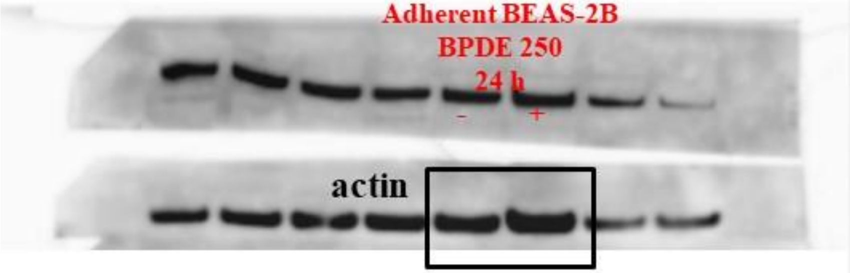

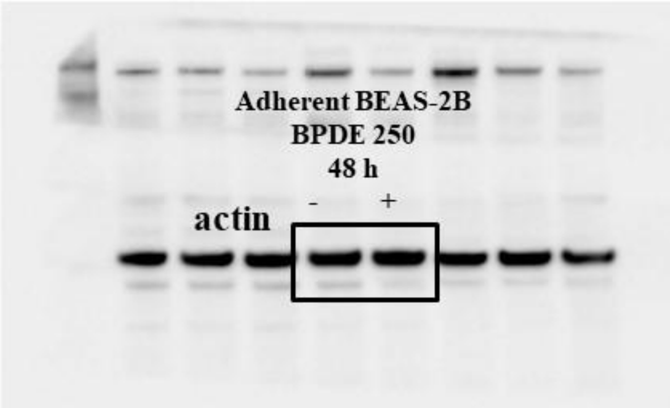

Fig 4B line 2 column 2

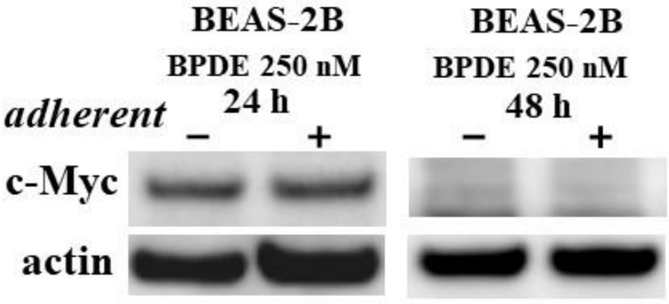

Fig 4B line 2 column 3

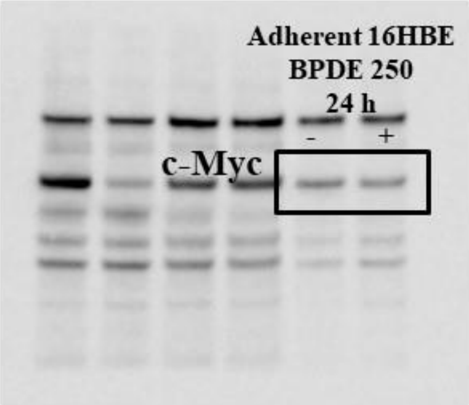

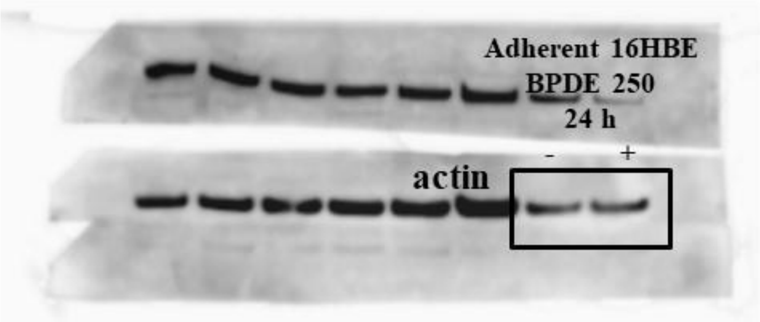

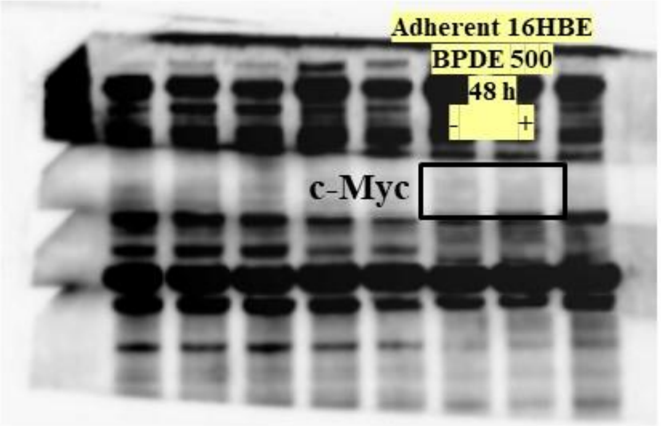

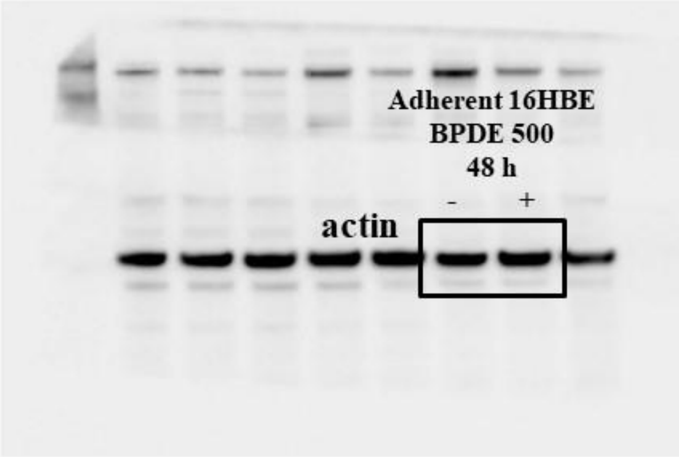

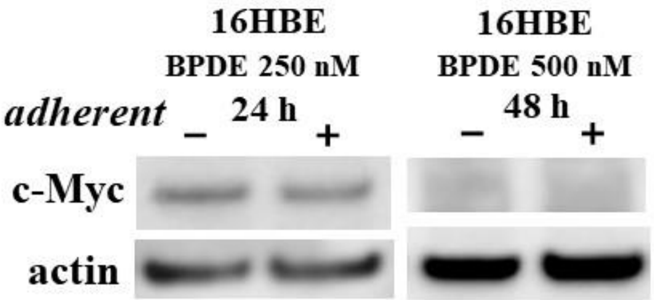

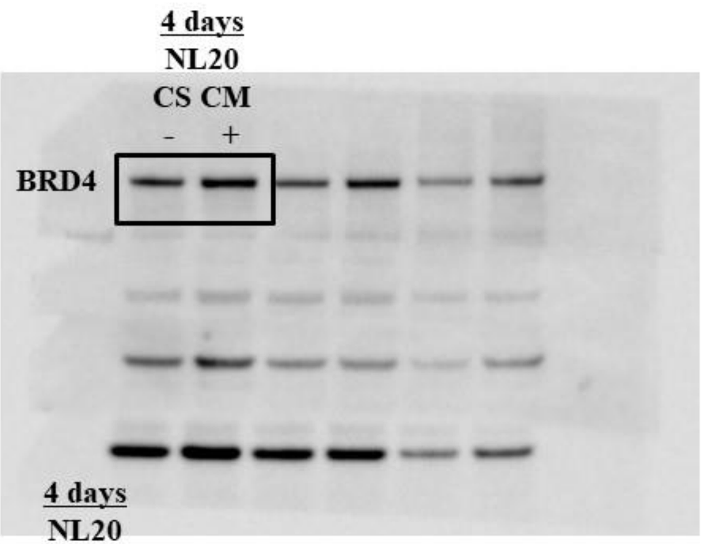

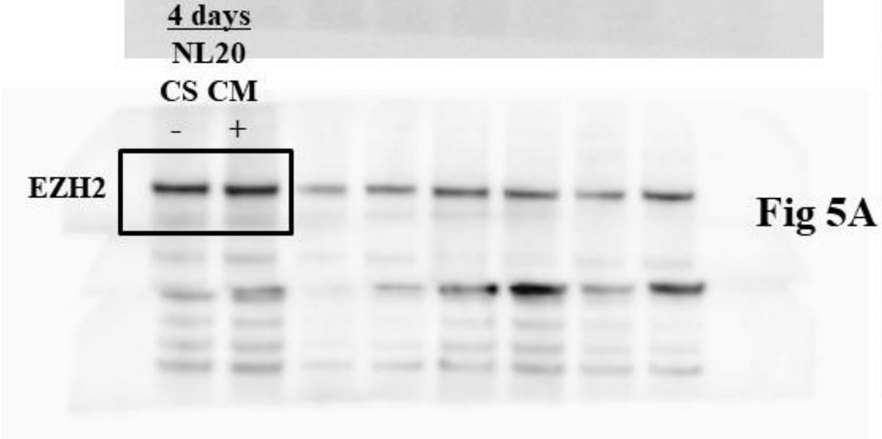

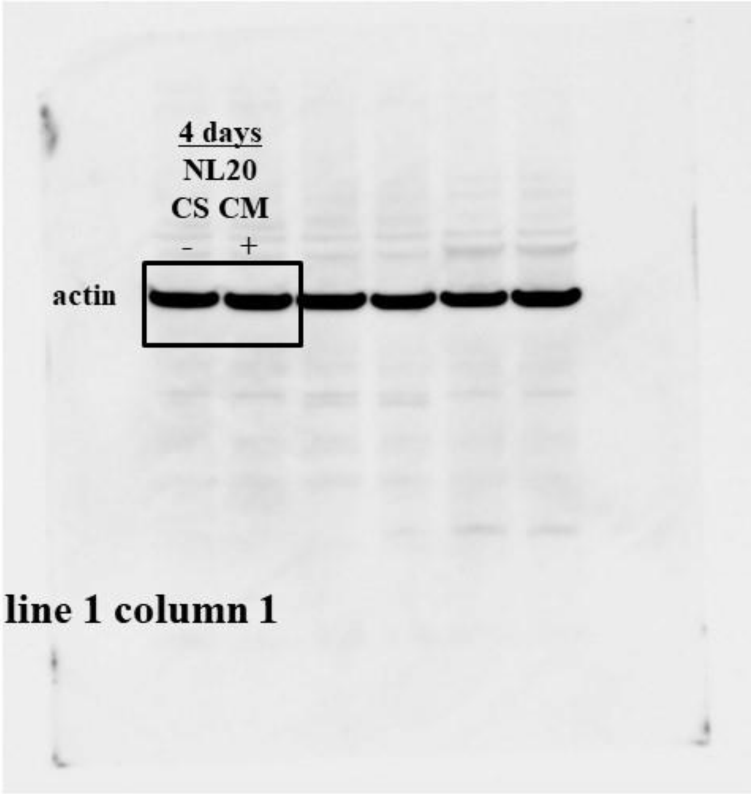

Fig 5A line 1 column 1

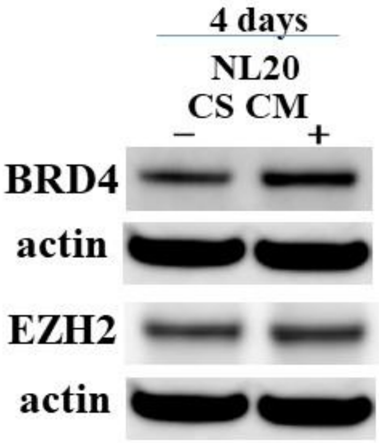

Fig 5A line 1 column 2

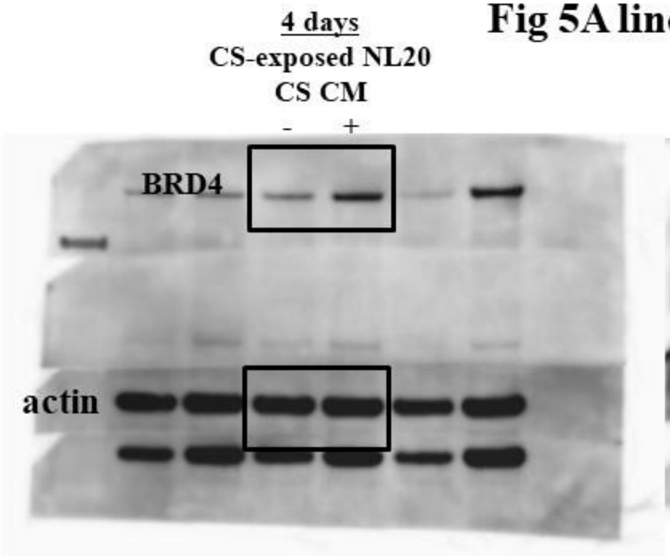

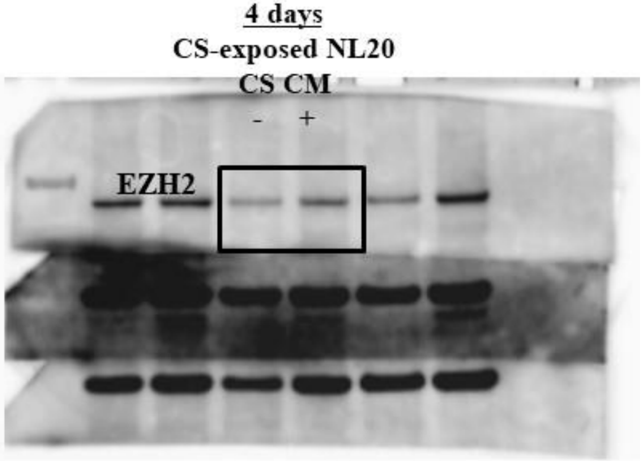

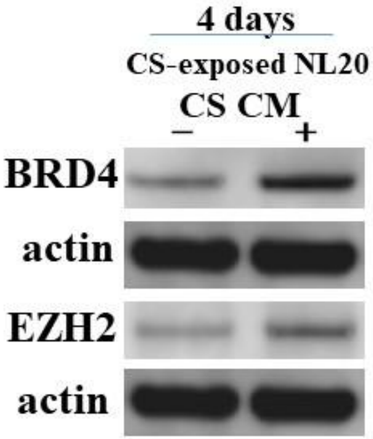

Fig 5A line 1 column 3

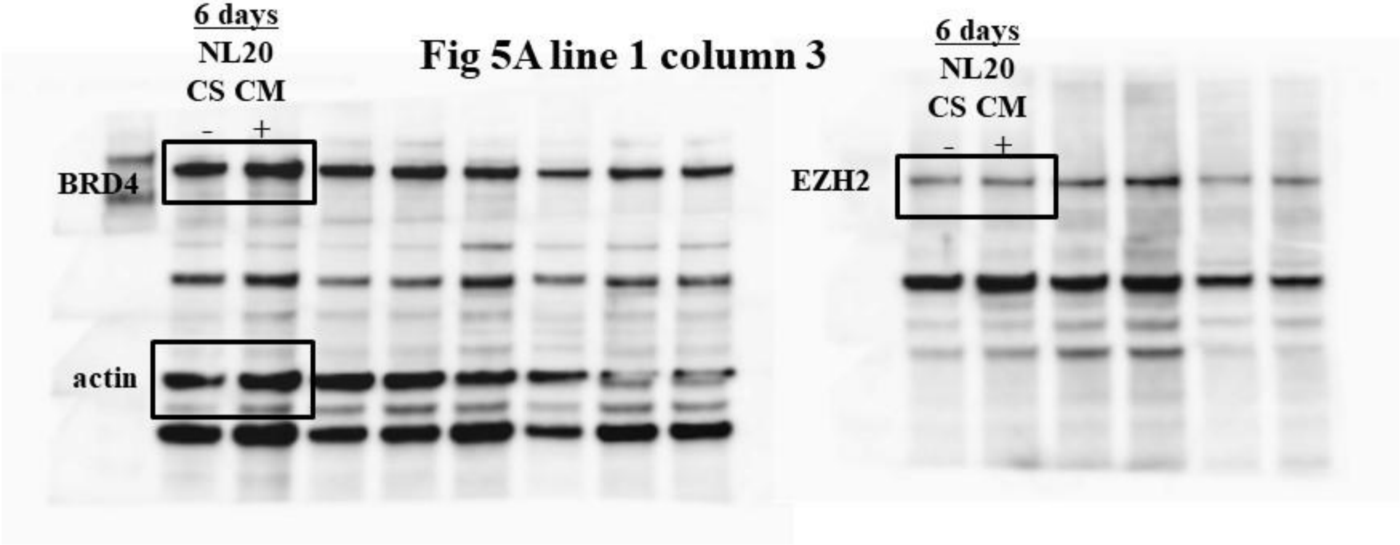

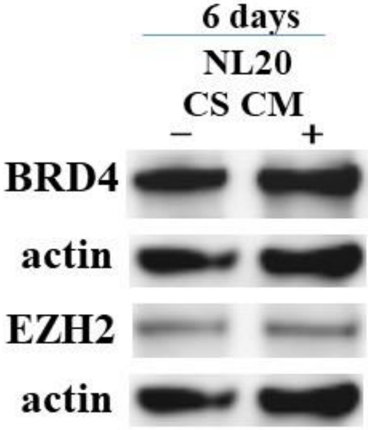

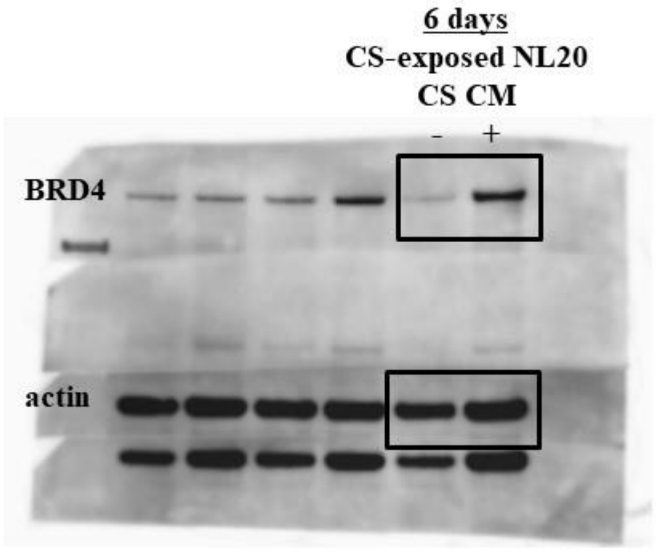

Fig 5A line 1 column 4

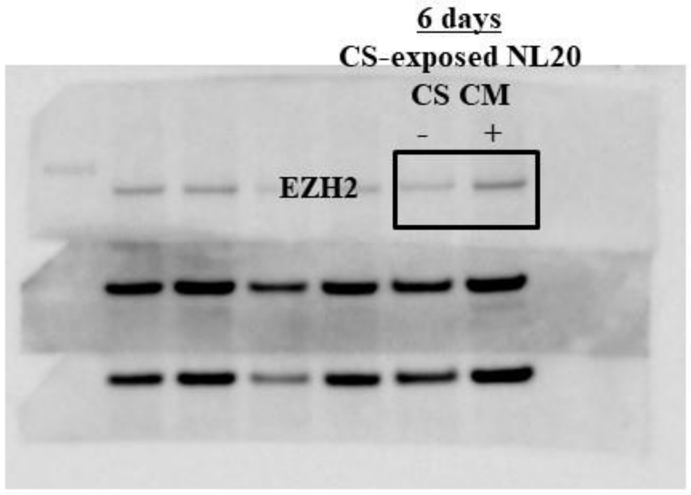

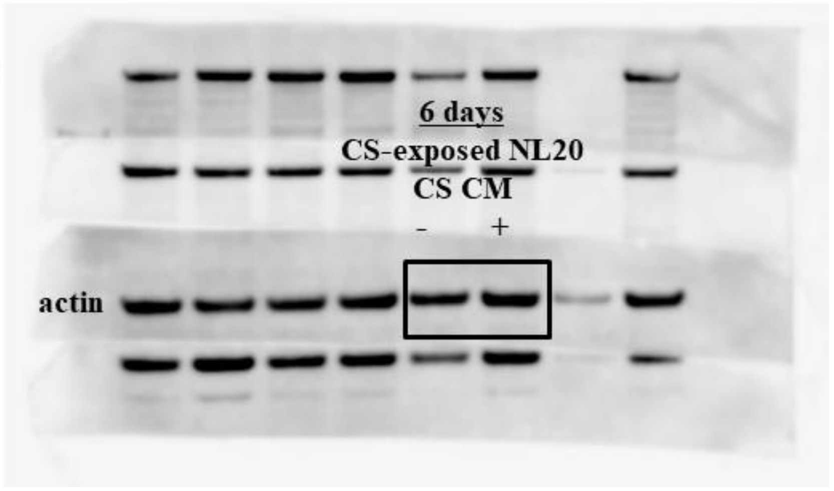

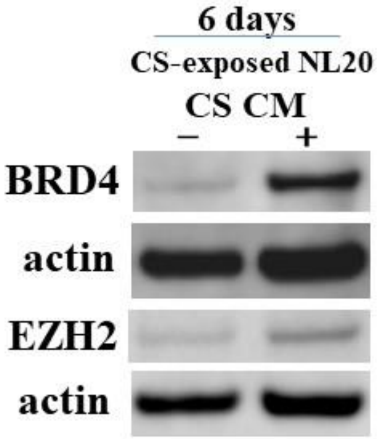

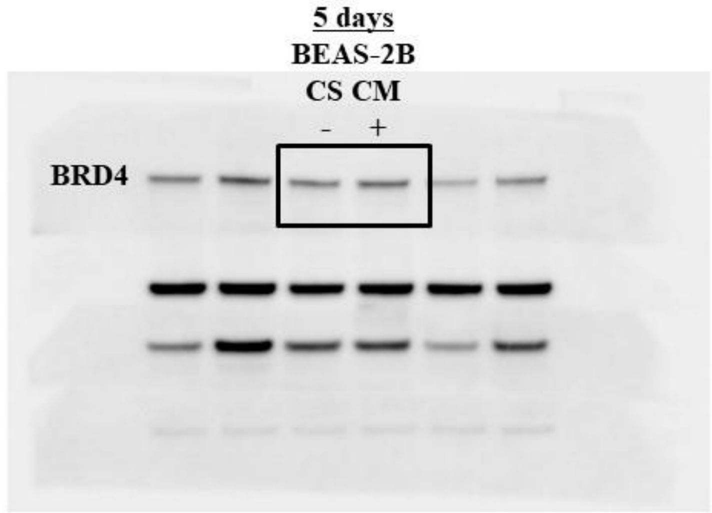

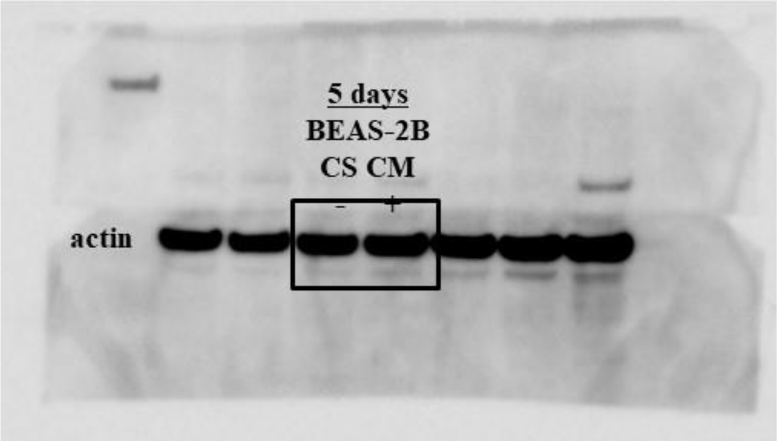

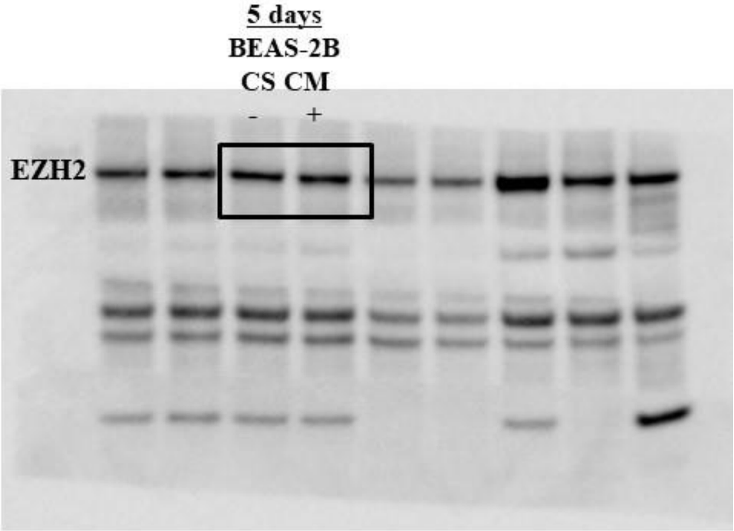

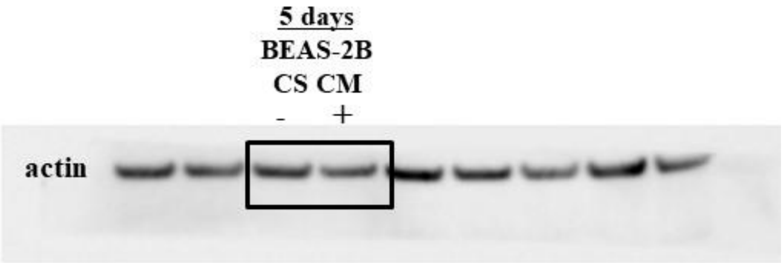

Fig 5A line 2 column 1

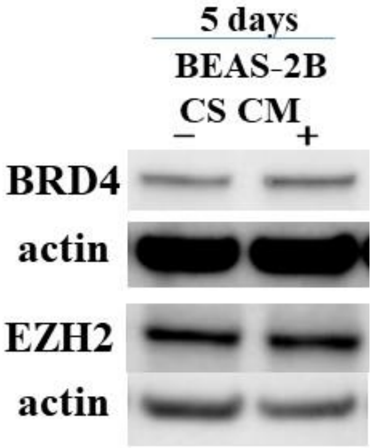

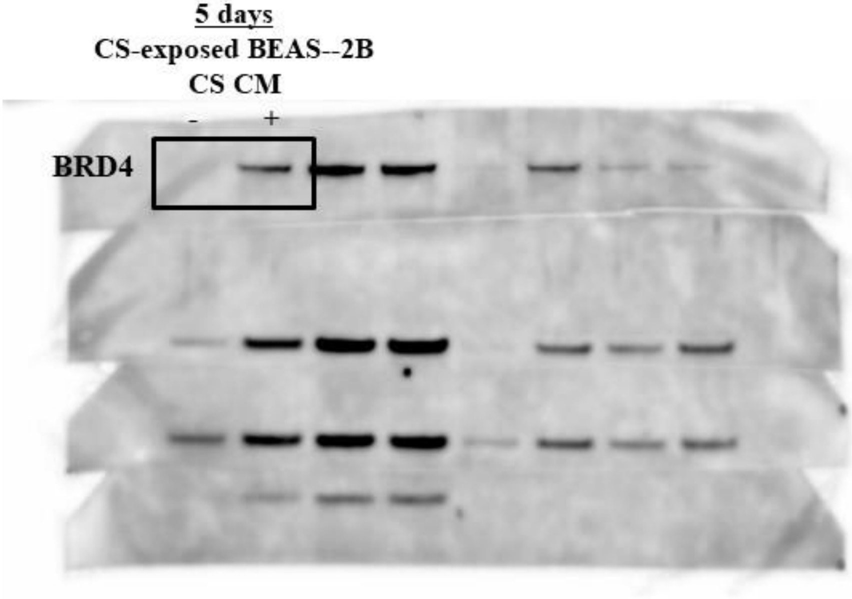

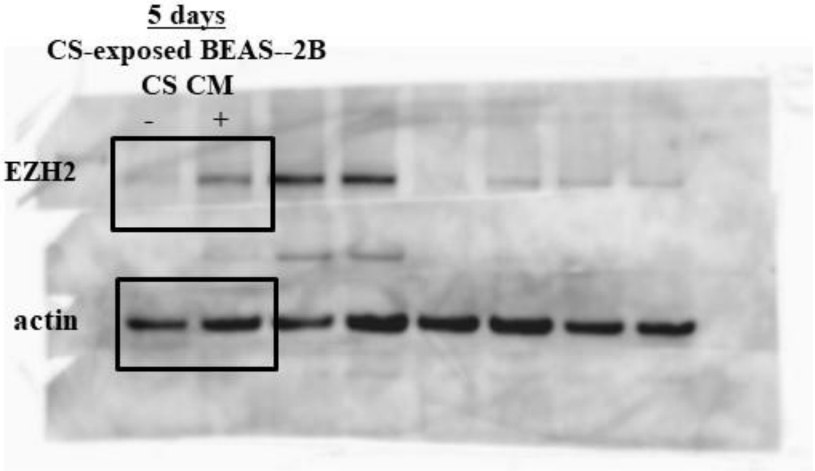

Fig 5A line 2 column 2

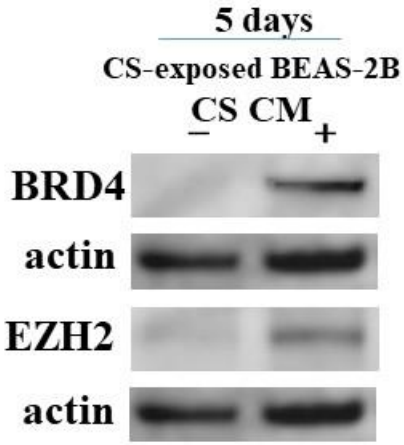

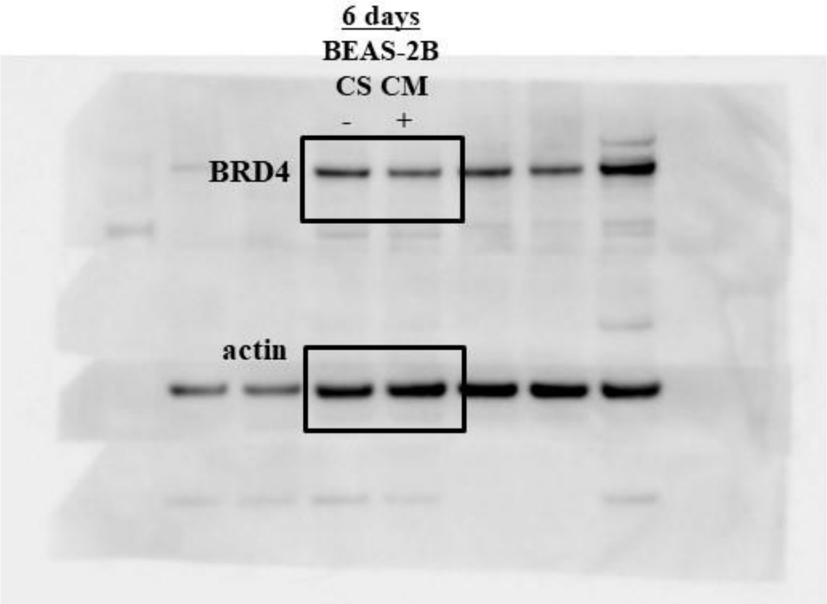

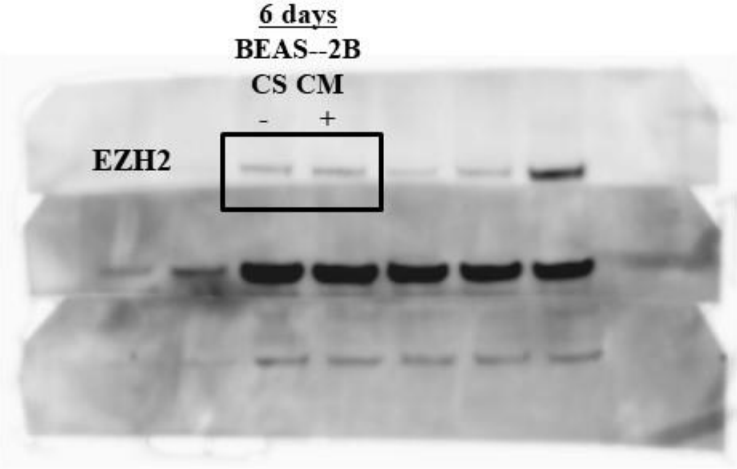

Fig 5A line 2 column 3

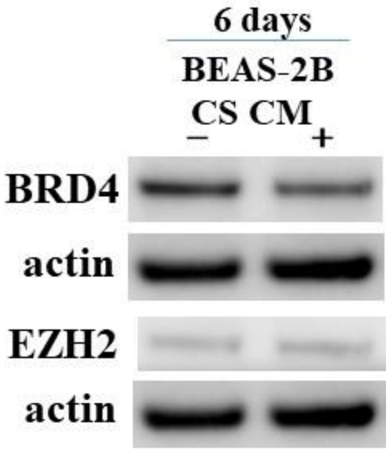

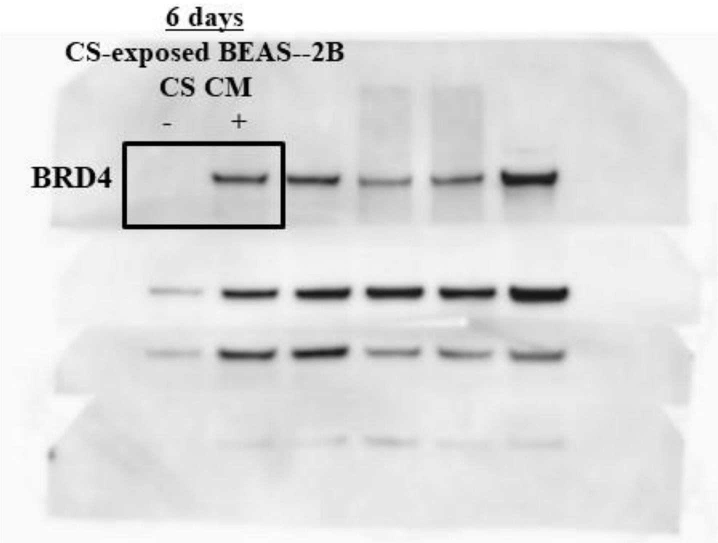

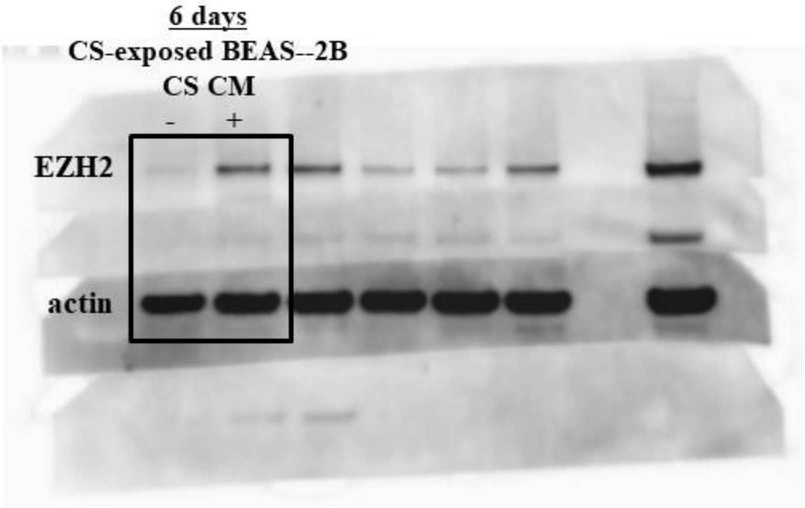

Fig 5A line 2 column 4

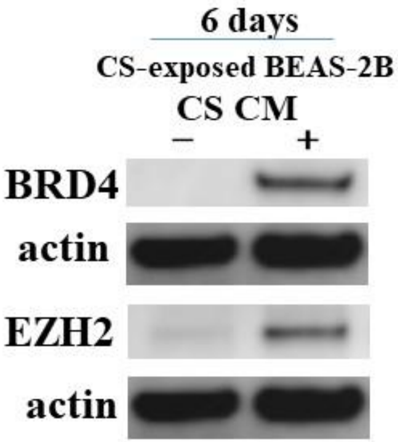

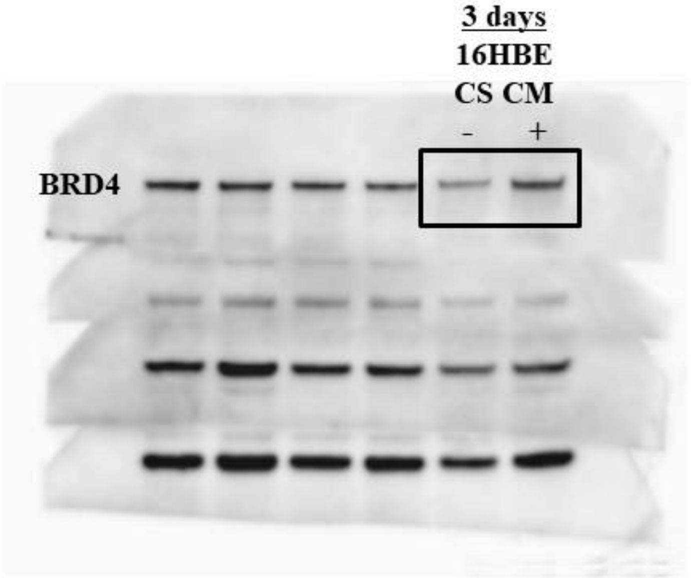

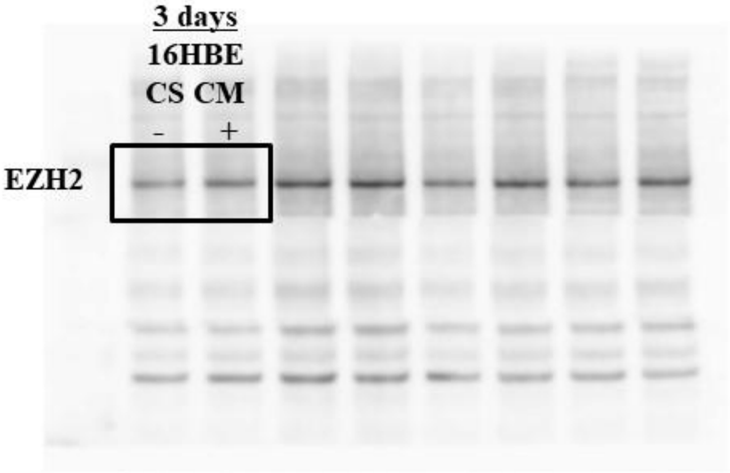

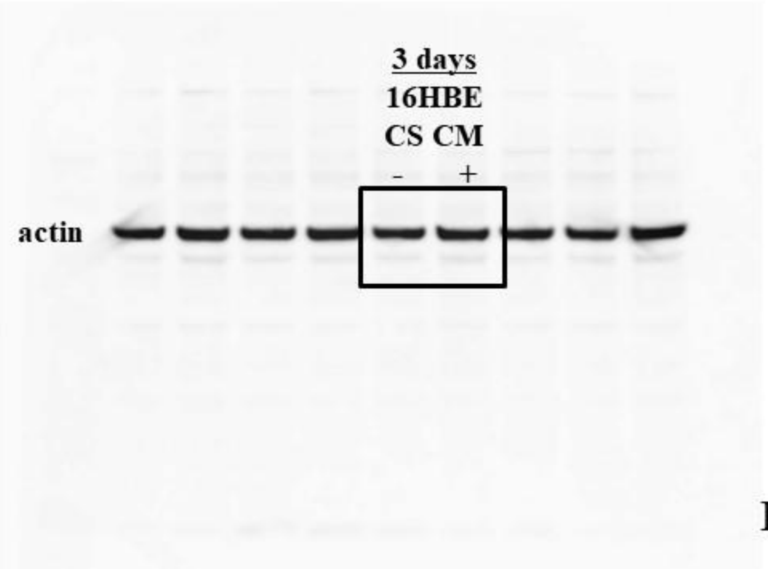

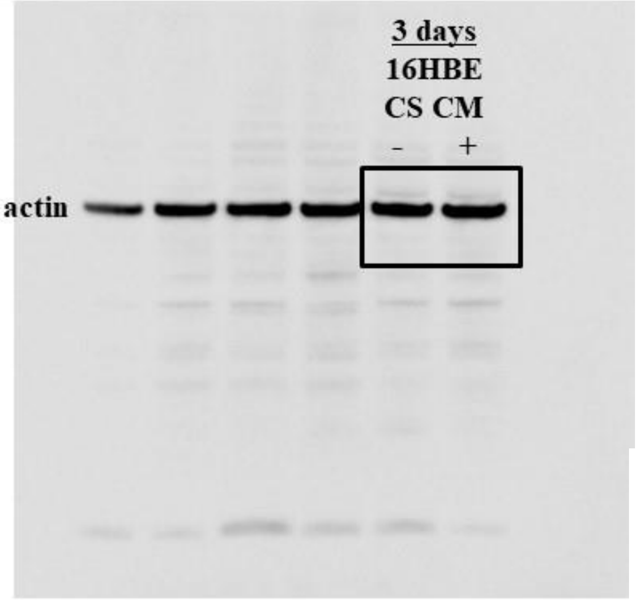

Fig 5A line 3 column 1

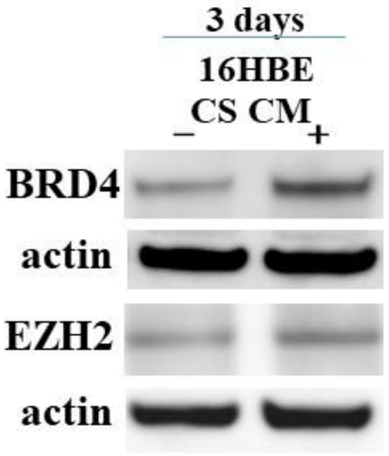

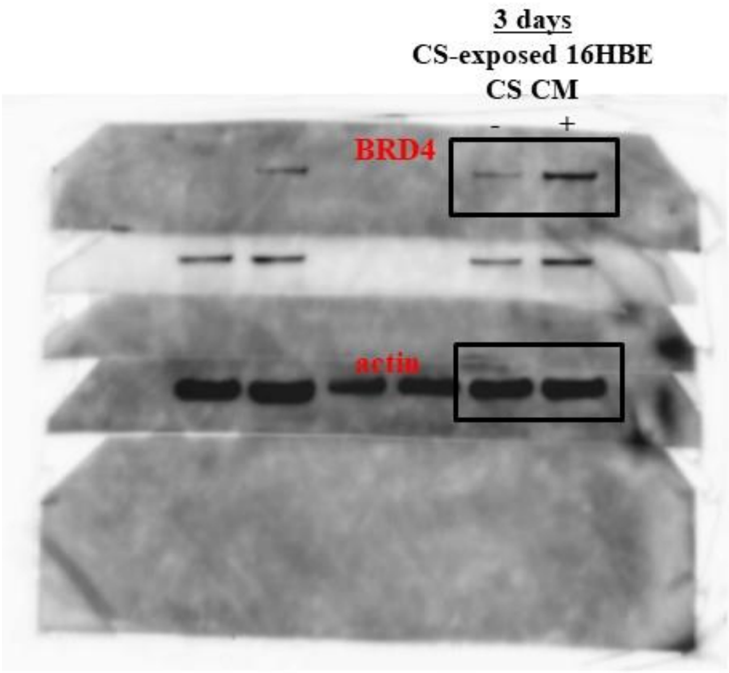

Fig 5A line 3 column 2

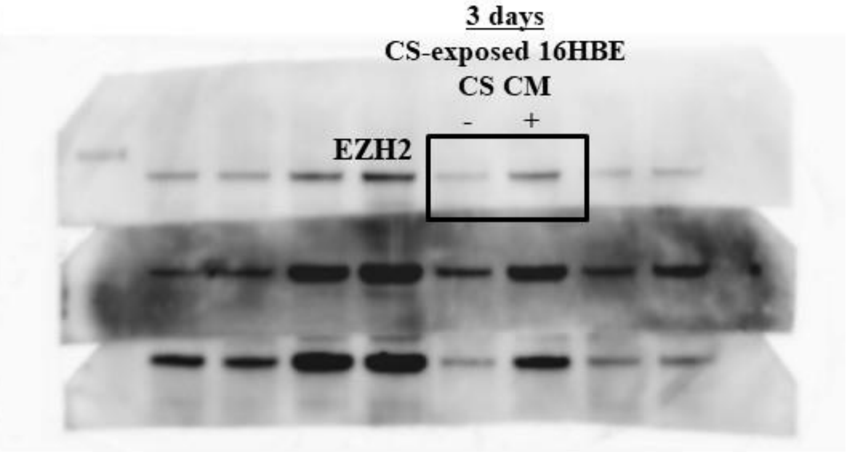

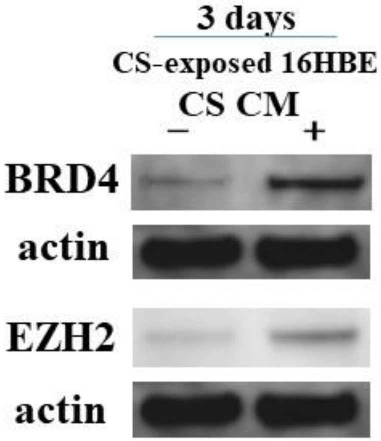

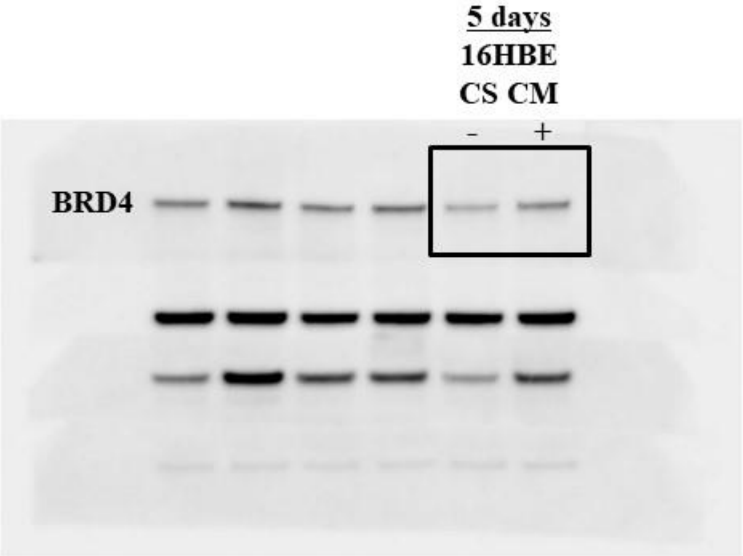

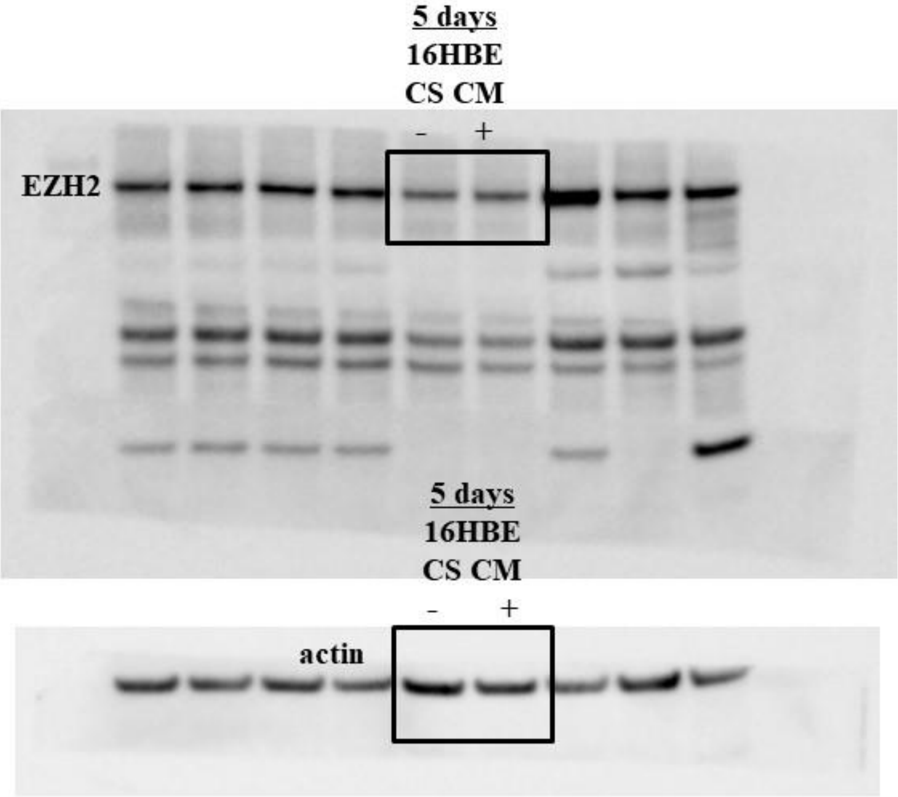

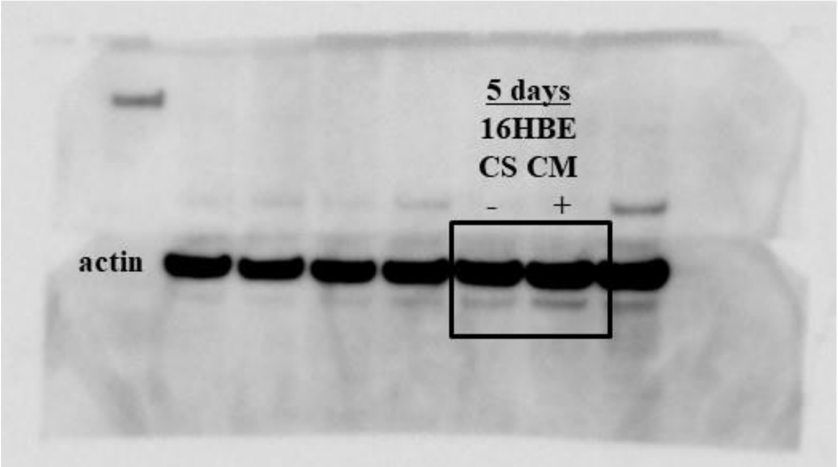

Fig 5A line 3 column 3

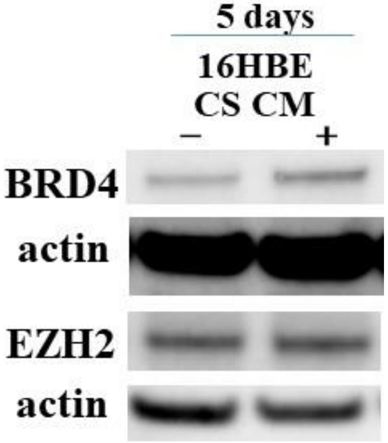

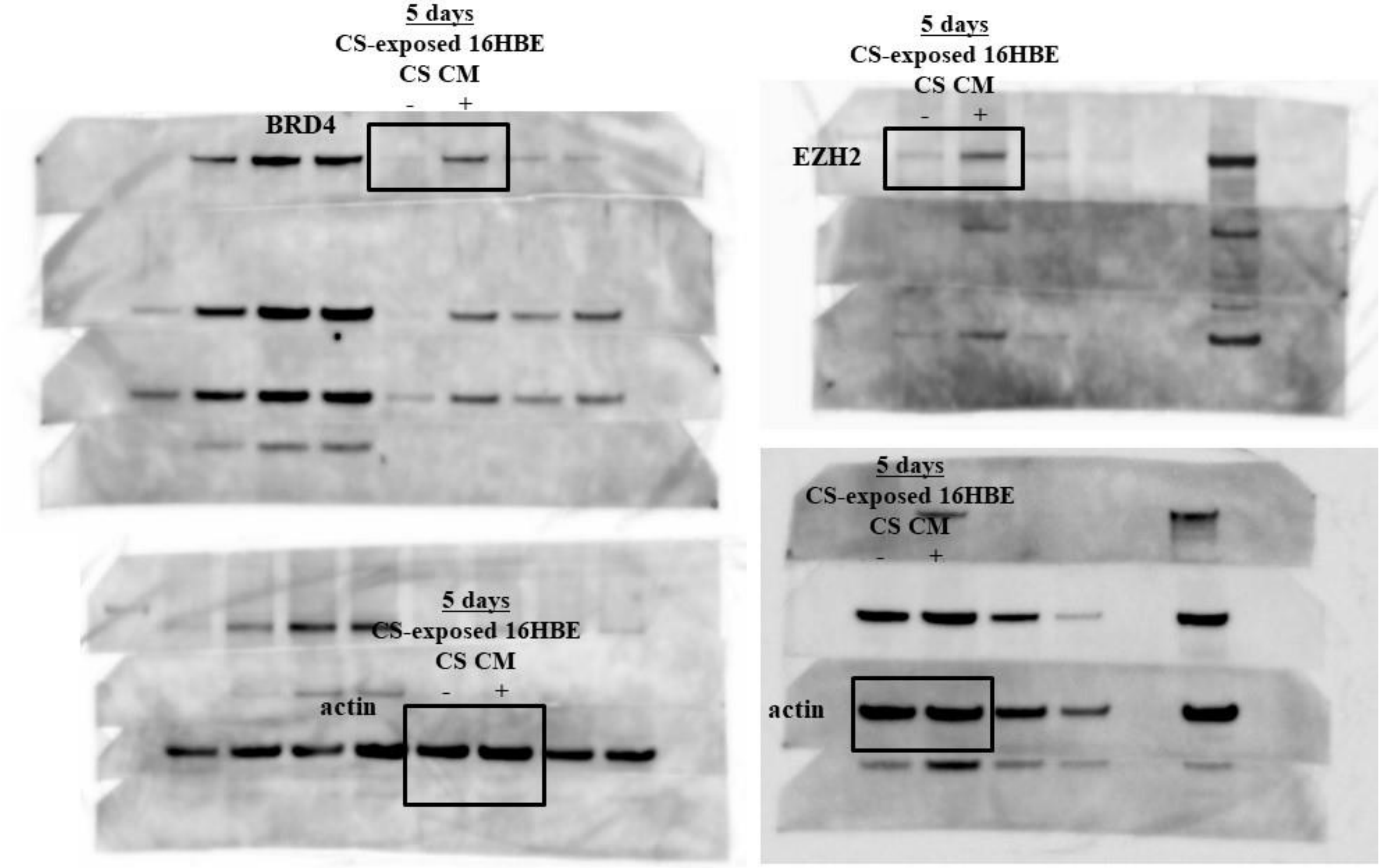

Fig 5A line 3 column 4

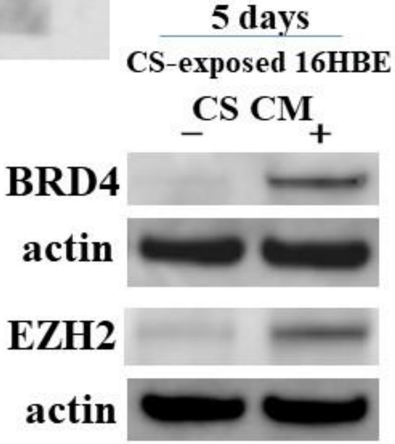

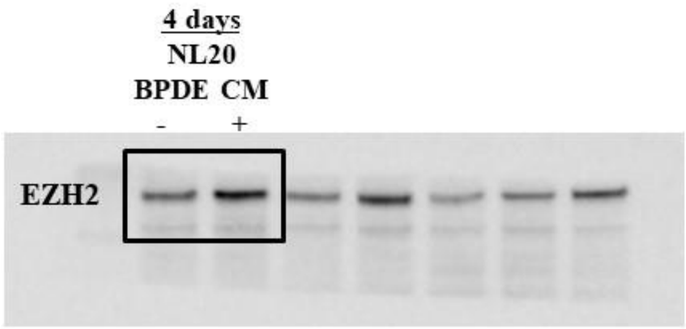

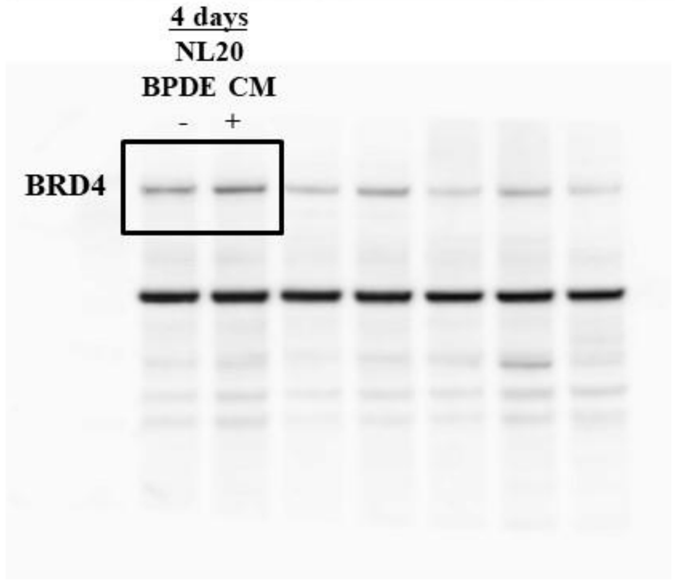

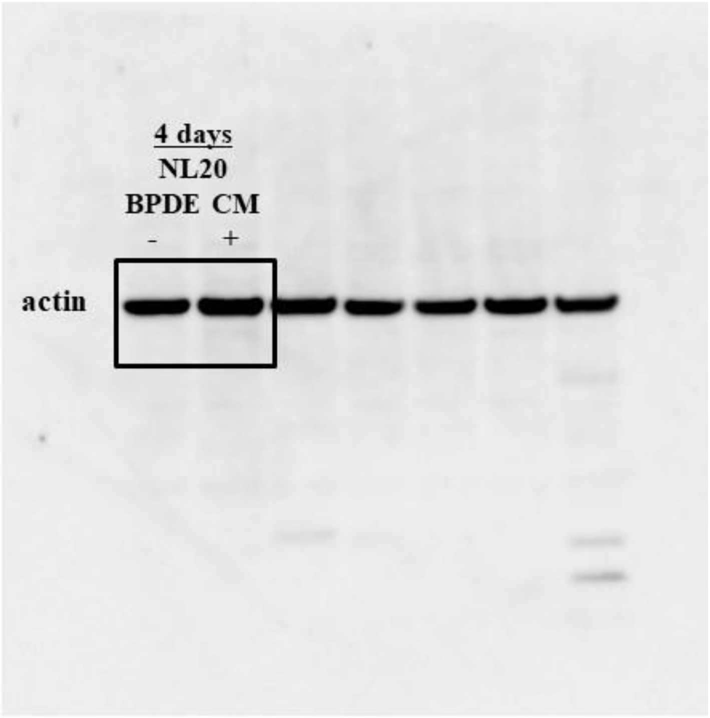

Fig 5A line 4 column 1

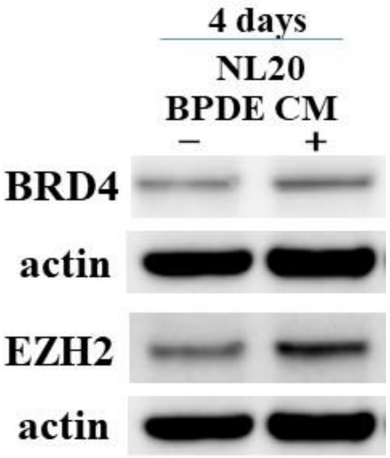

Fig 5A line 4 column 2

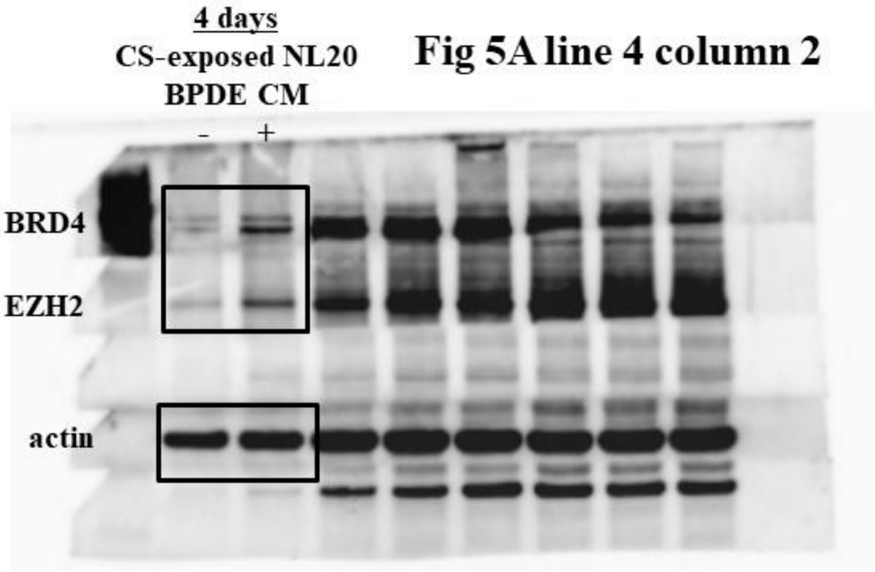

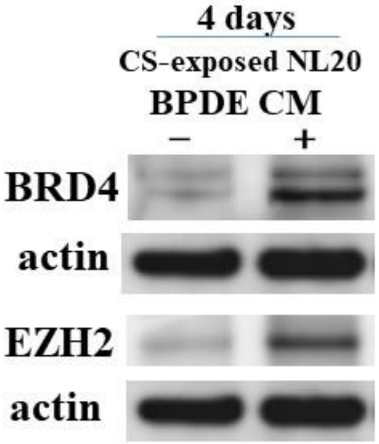

Fig 5A line 4 column 3

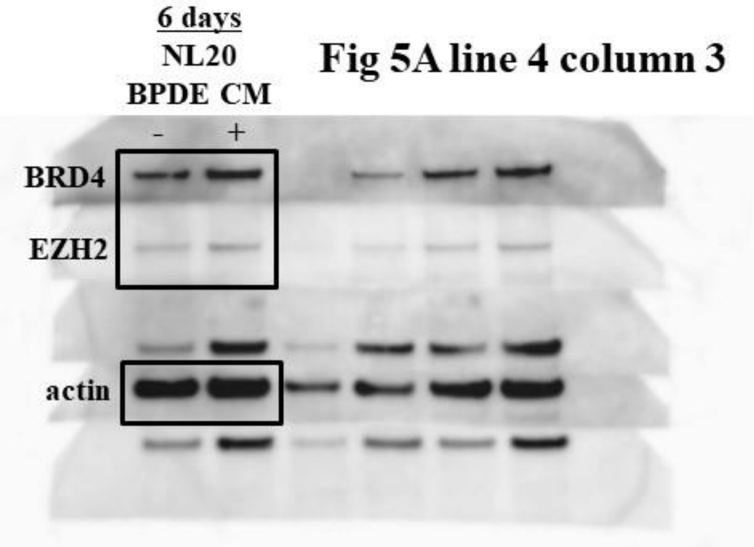

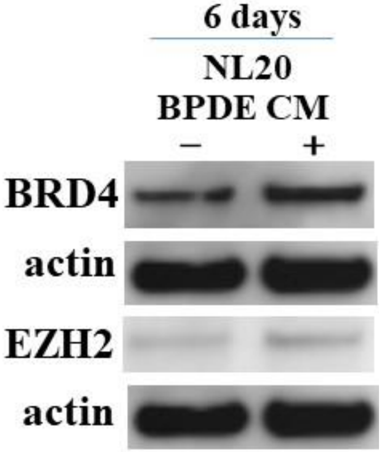

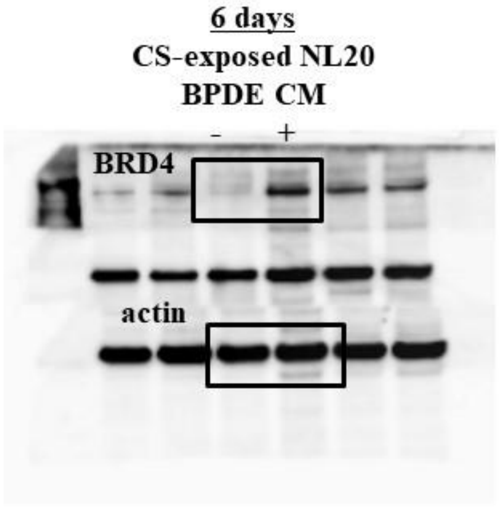

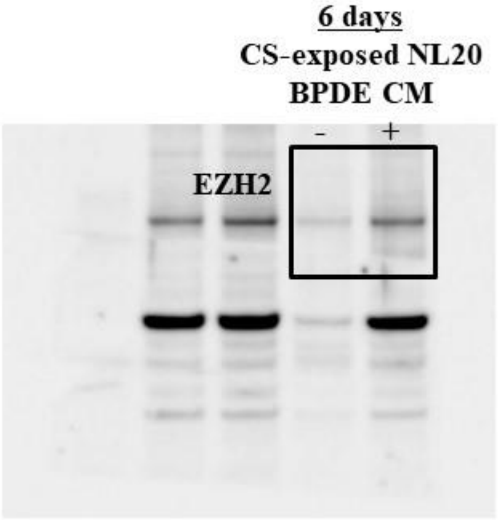

Fig 5A line 4 column 4

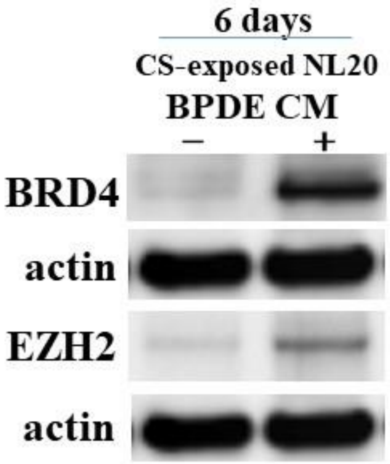

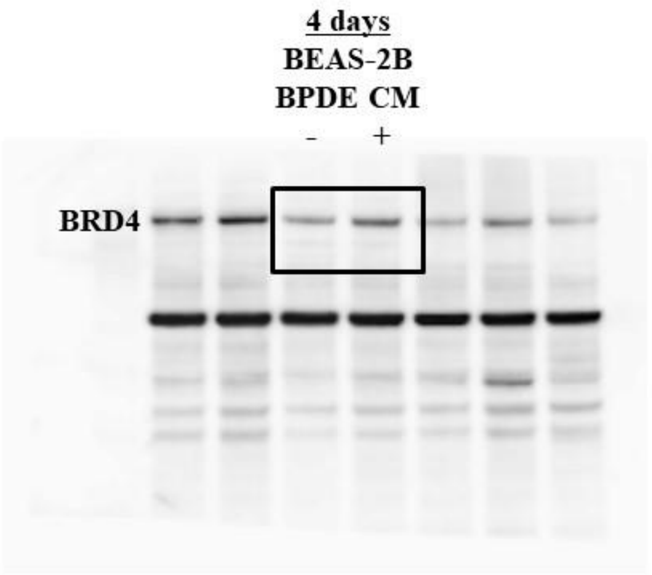

Fig 5A line 5 column 1

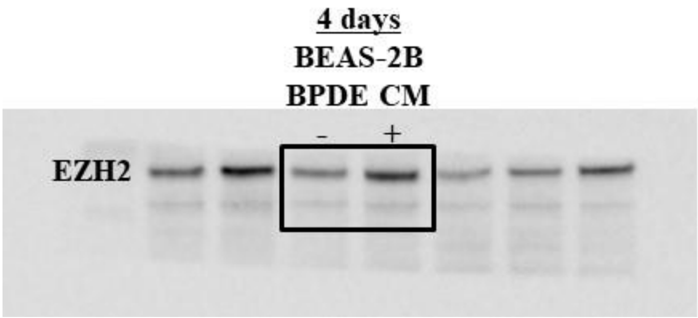

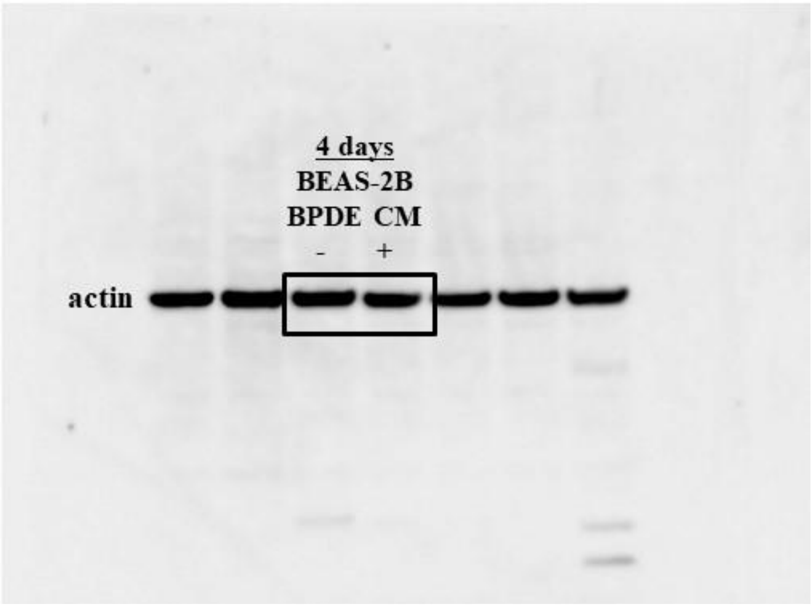

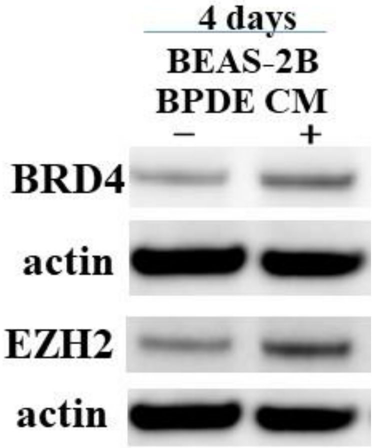

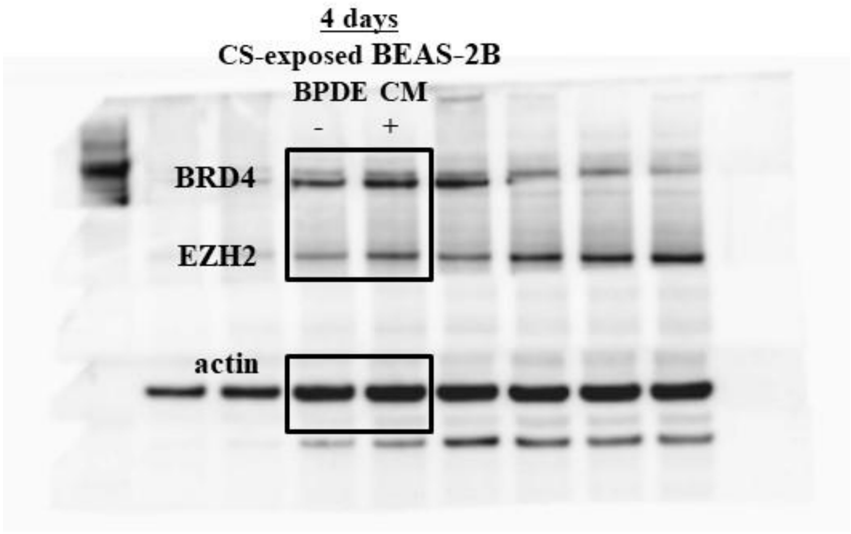

Fig 5A line 5 column 2

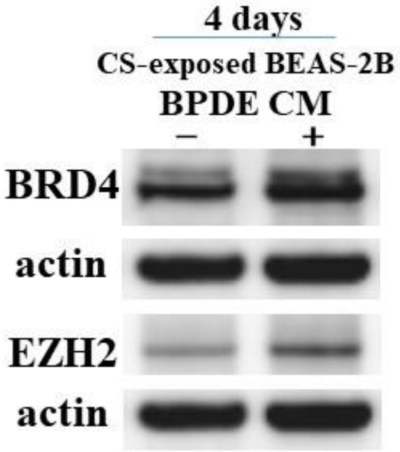

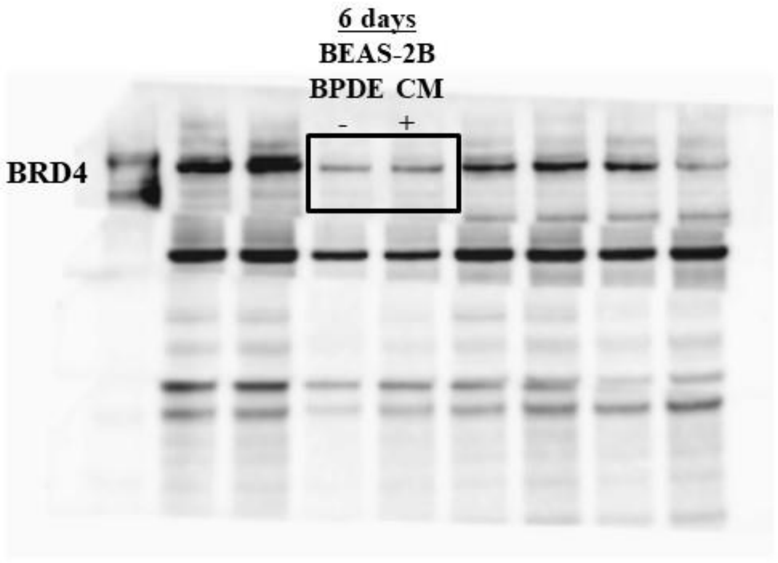

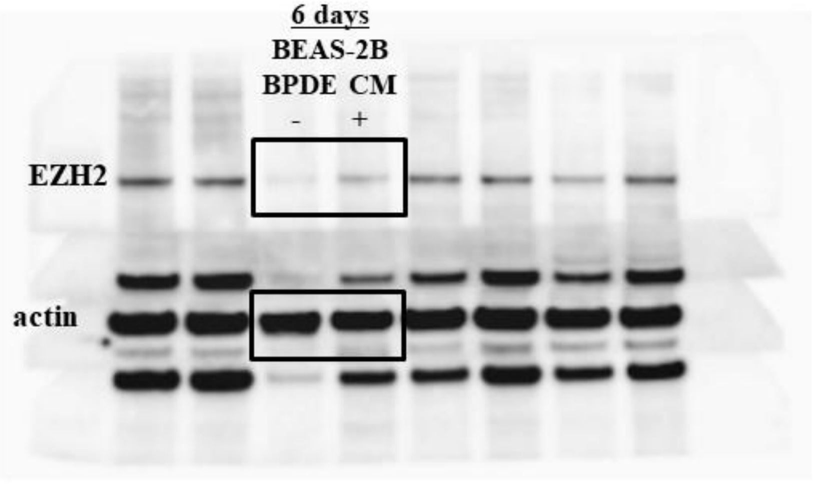

Fig 5A line 5 column 3

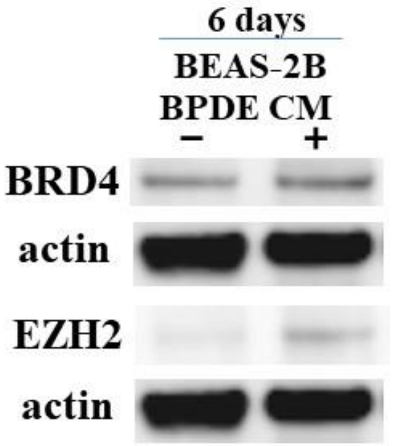

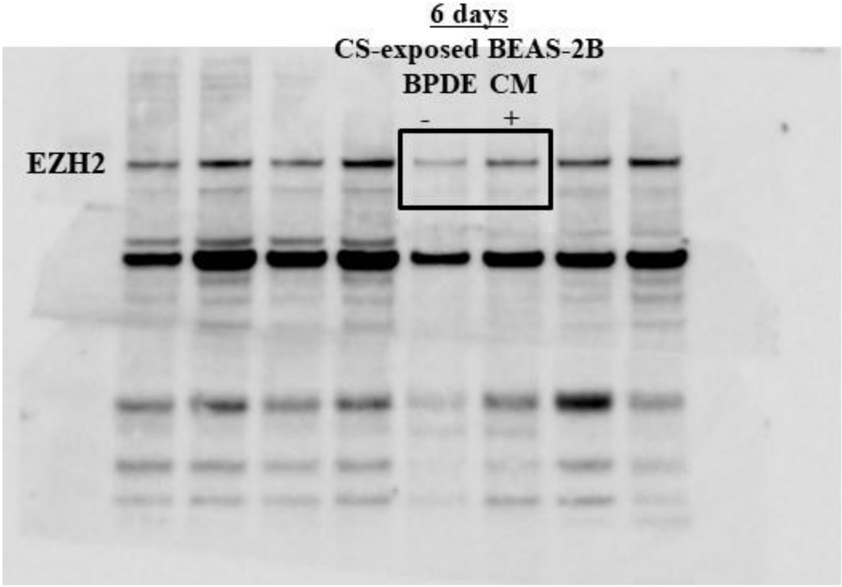

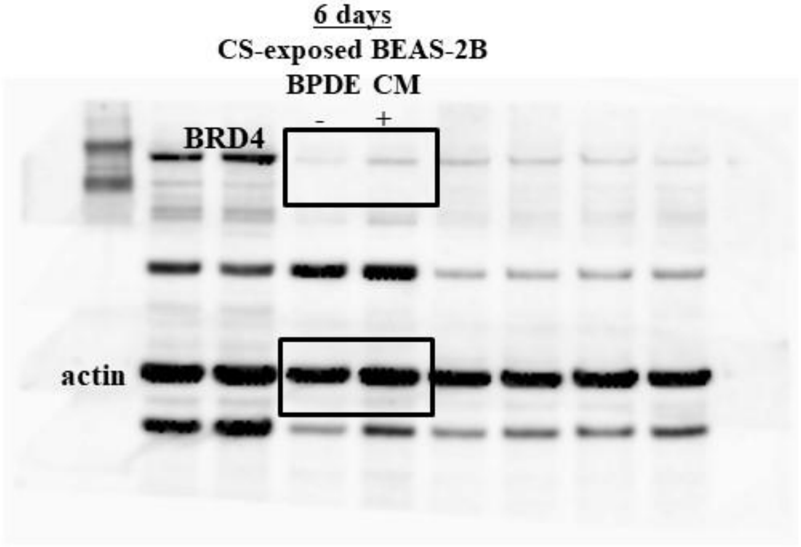

Fig 5A line 5 column 4

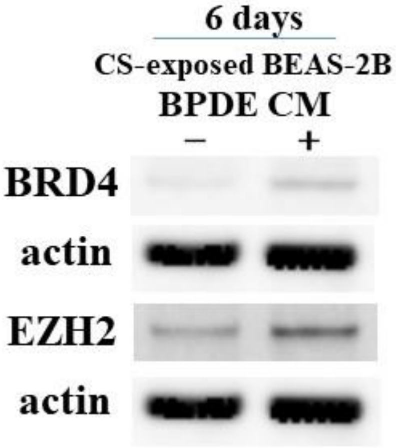

Fig 5A line 6 column 1

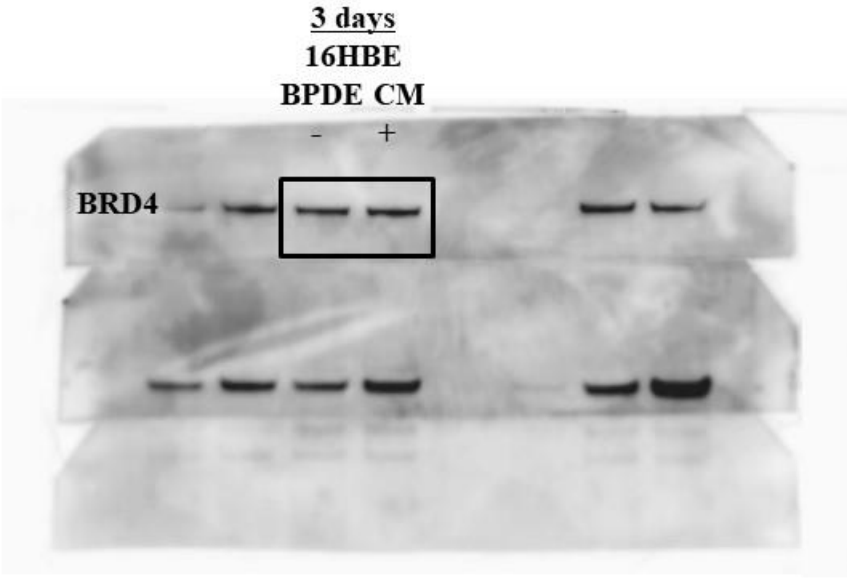

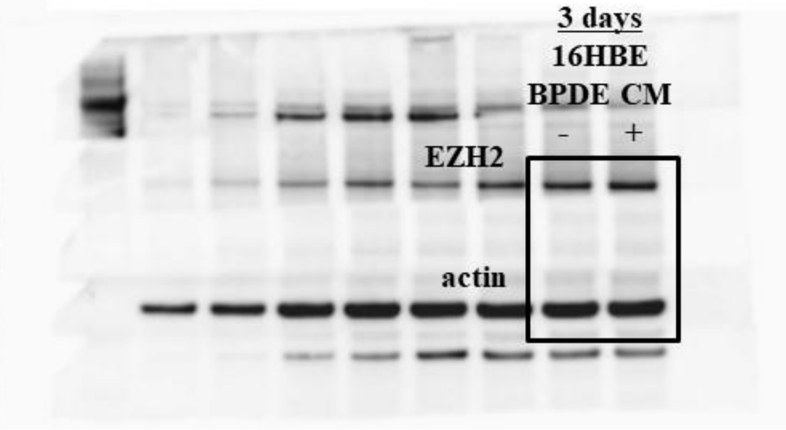

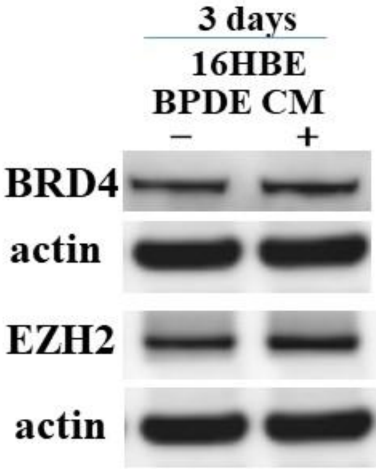

Fig 5A line 6 column 2

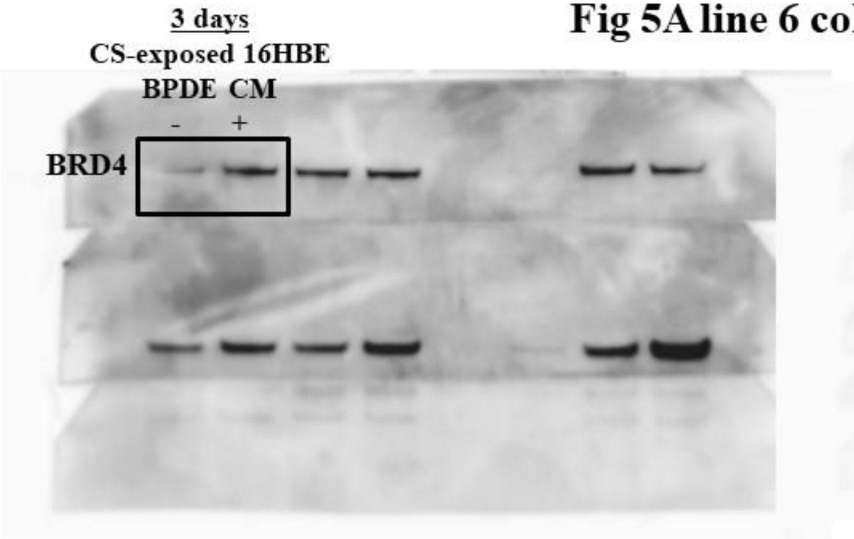

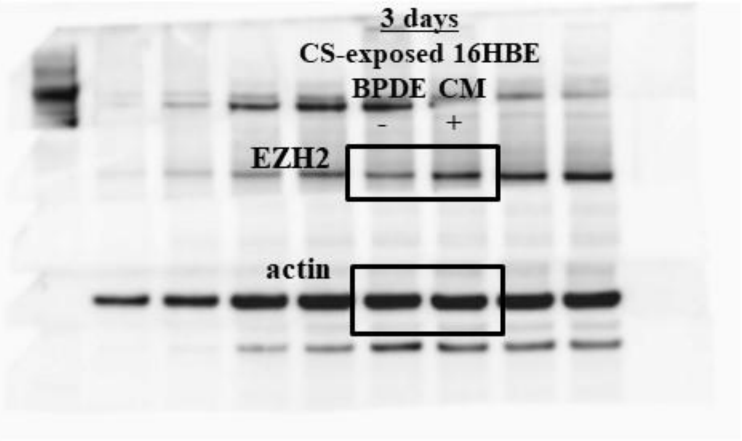

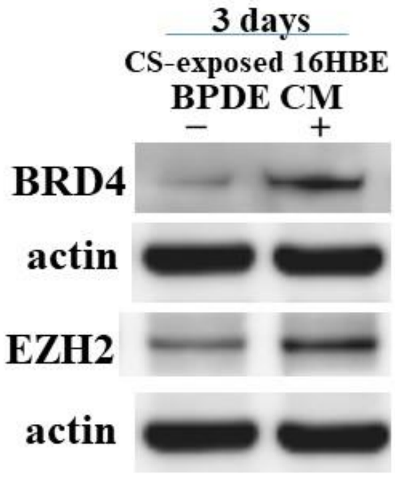

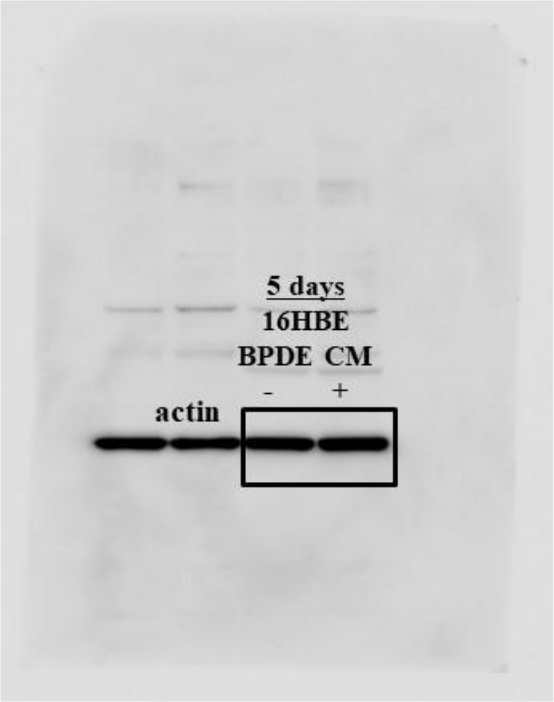

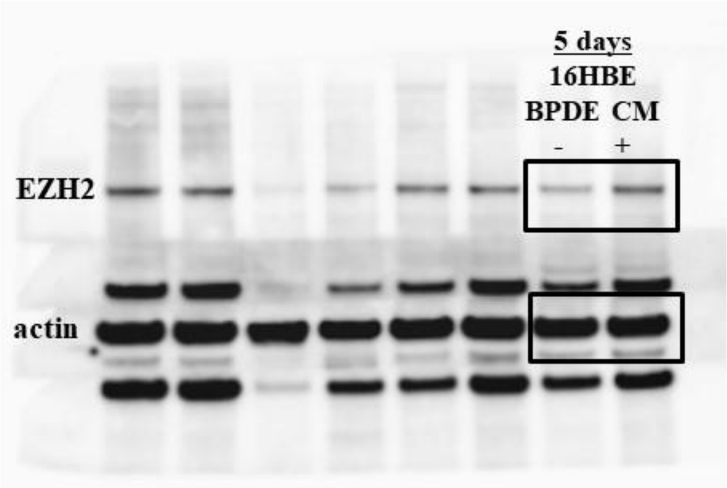

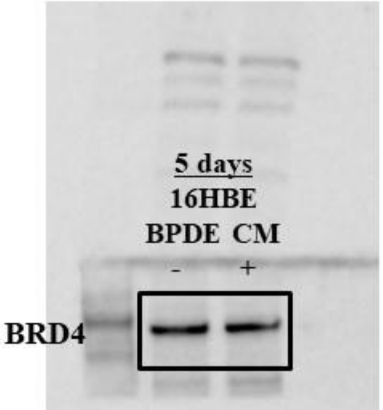

Fig 5A line 6 column 3

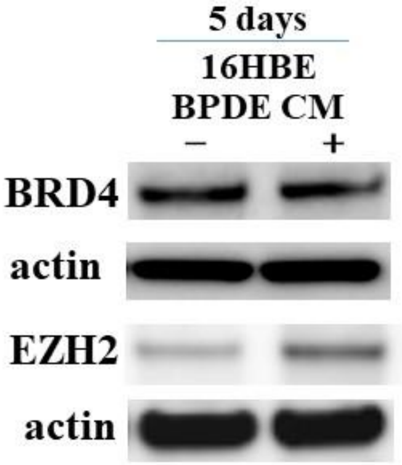

Fig 5A line 6 column 4

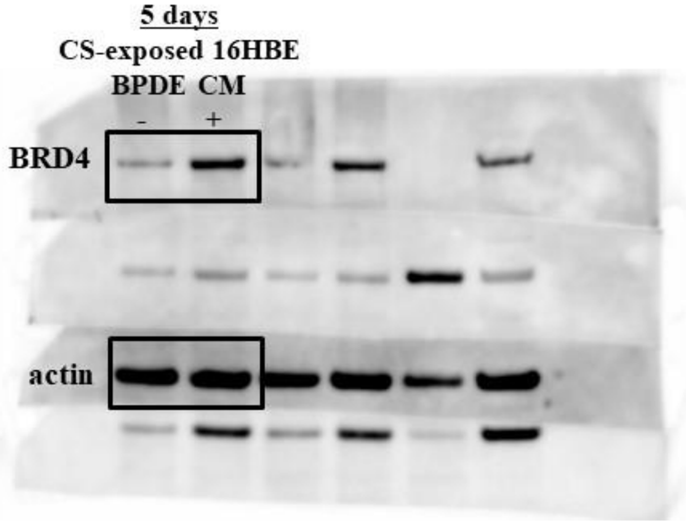

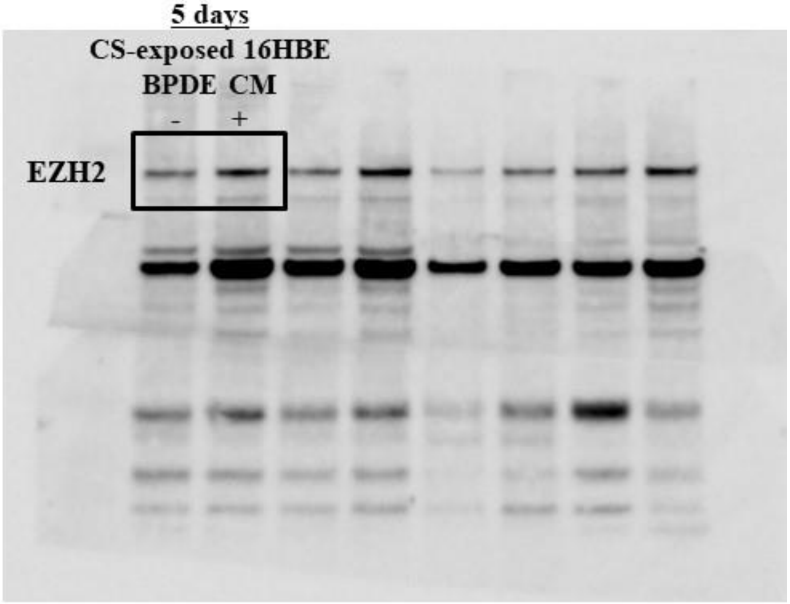

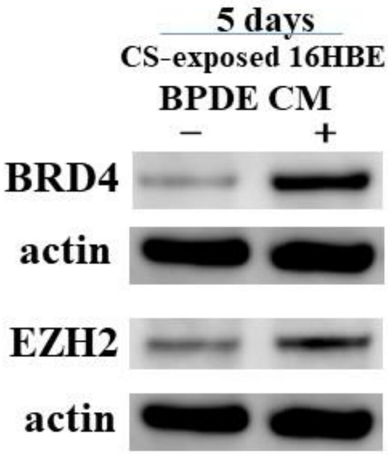

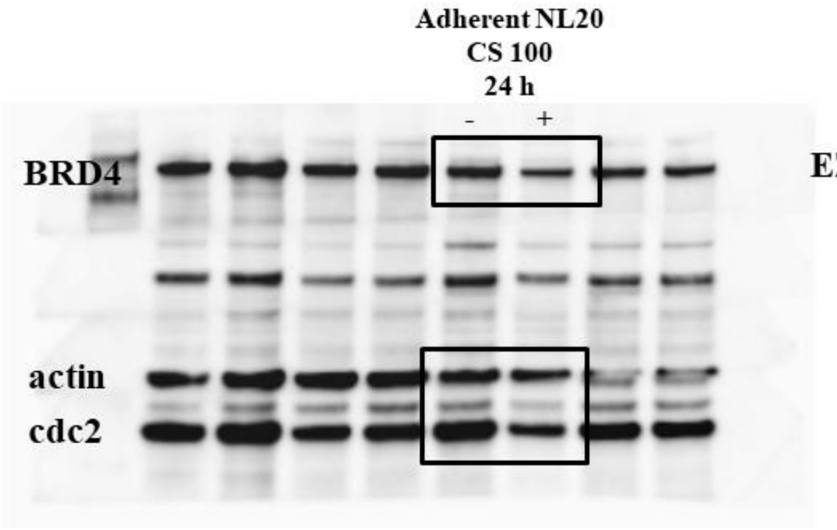

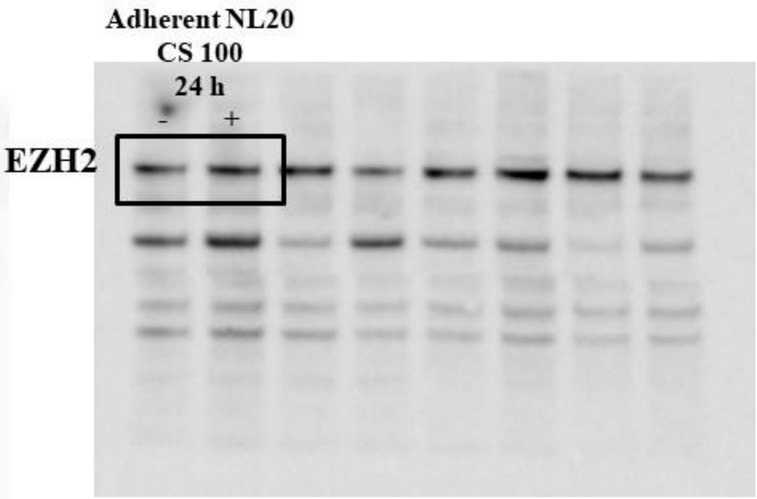

Fig 5B line 1 column 1

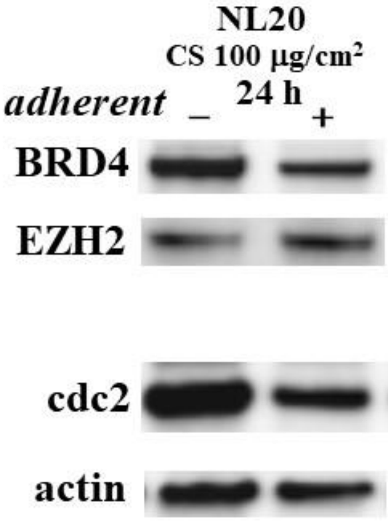

Fig 5B line 1 column 2

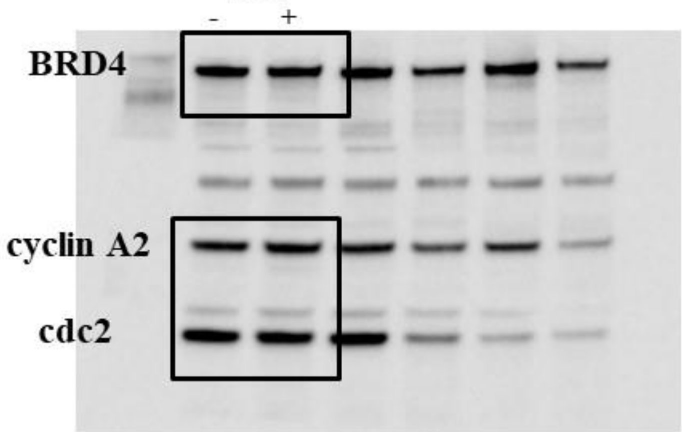

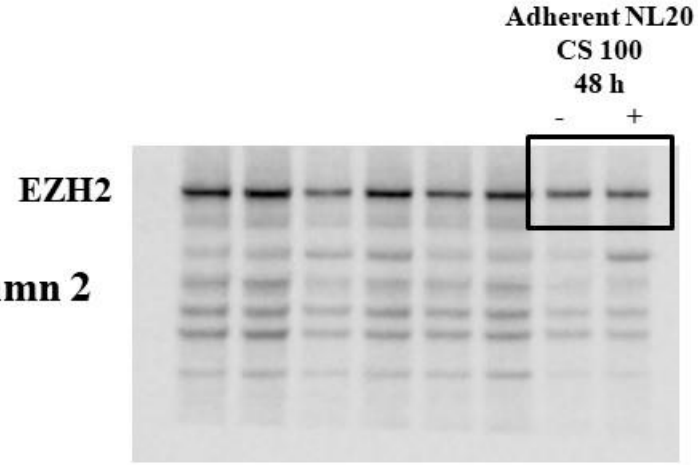

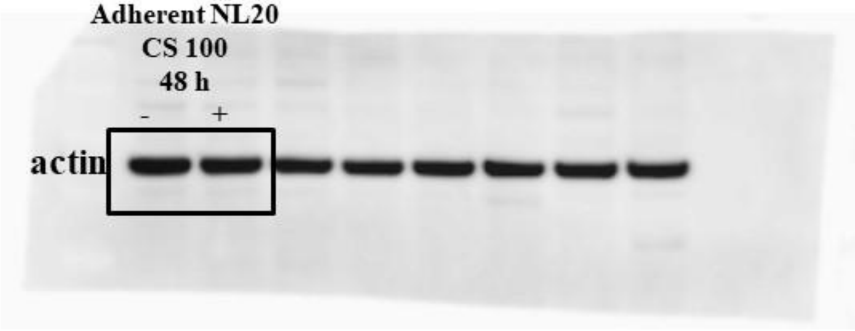

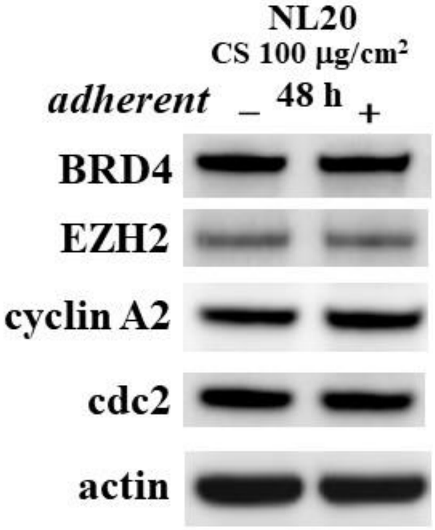

Fig 5B line 1 column 3

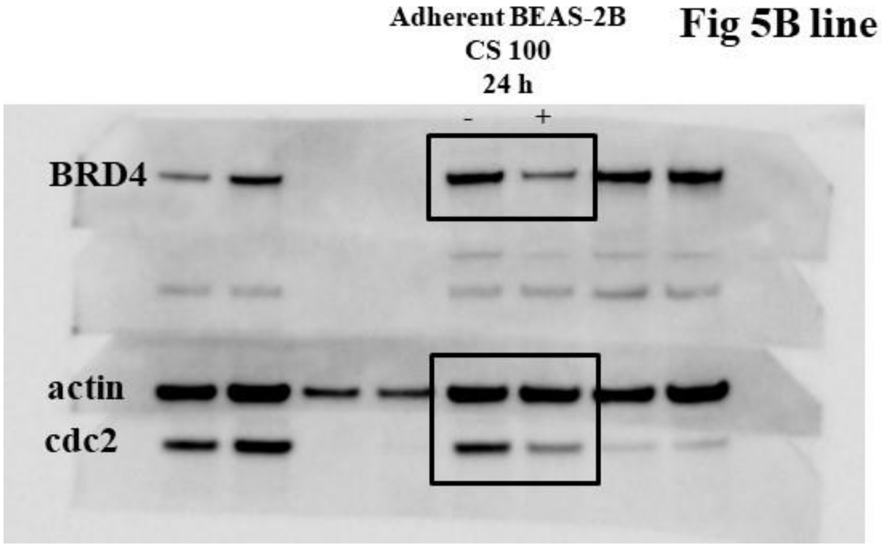

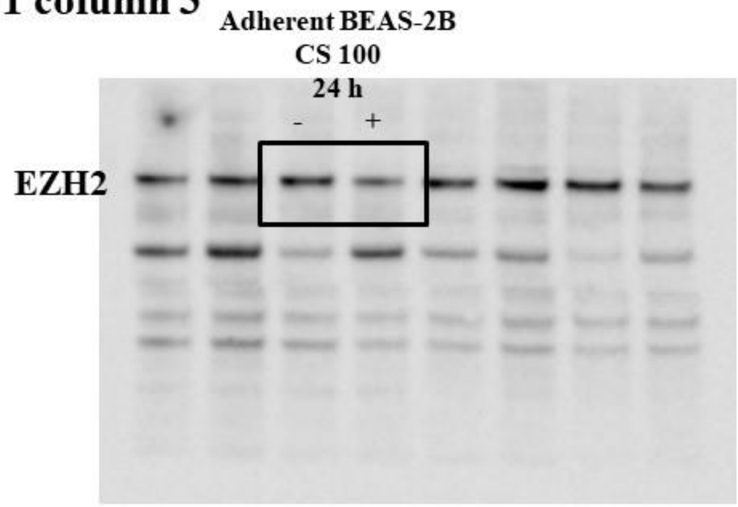

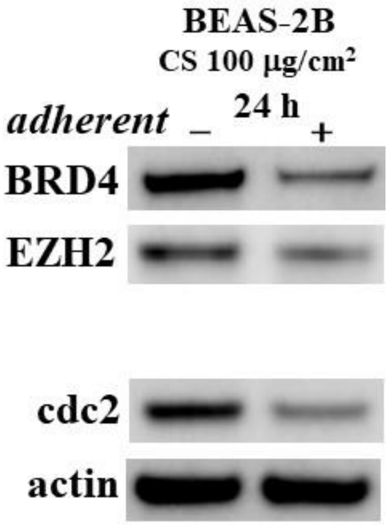

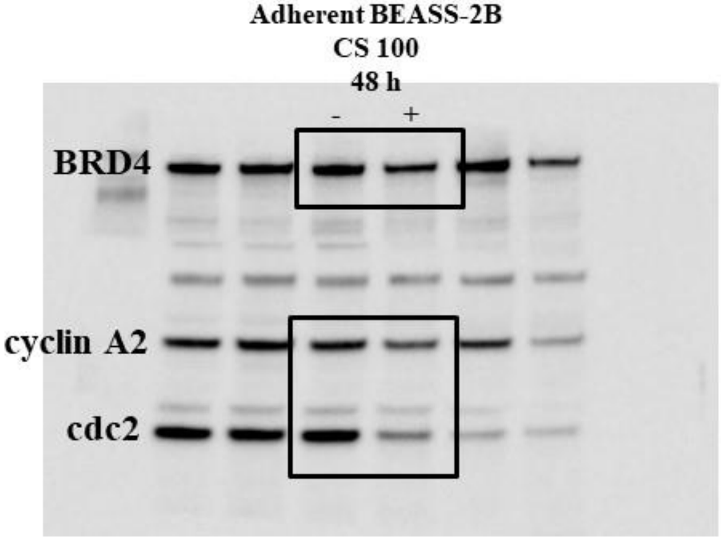

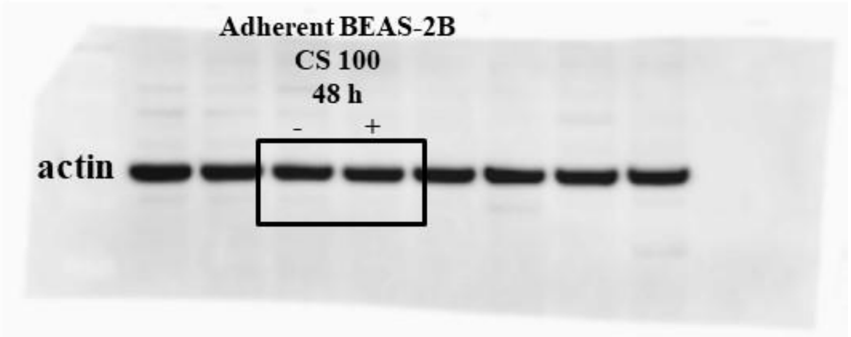

Fig 5B line 1 column 4

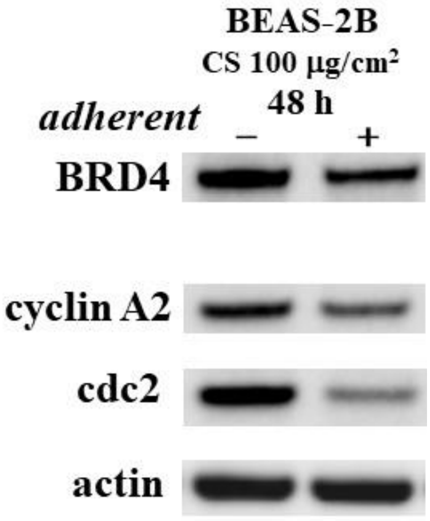

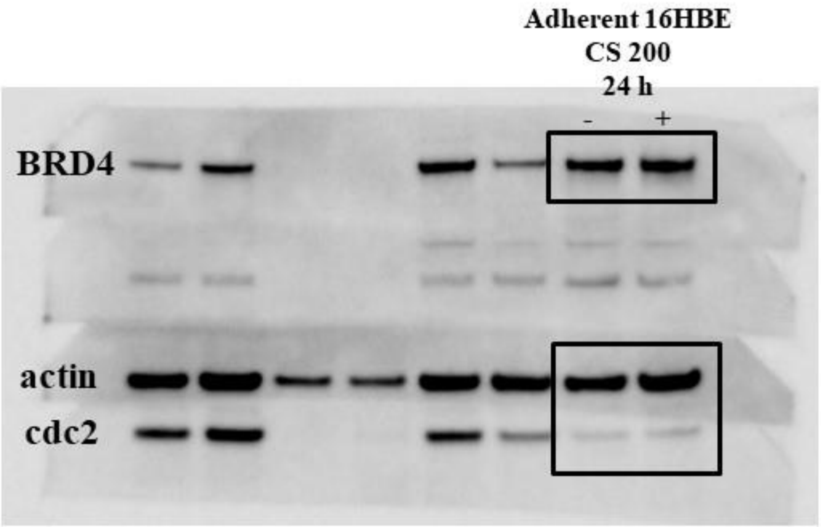

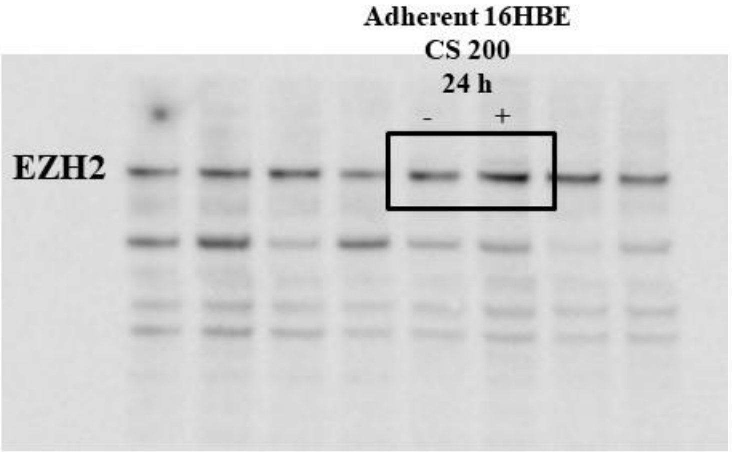

Fig 5B line 1 column 5

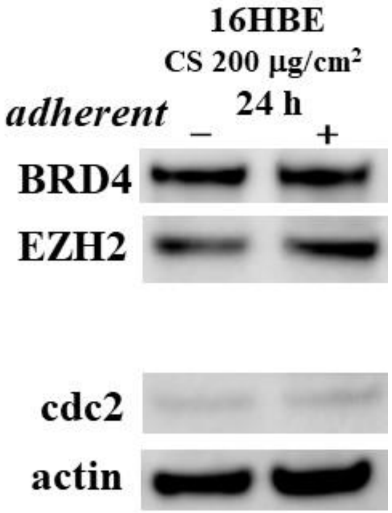

Fig 5B line 1 column 6

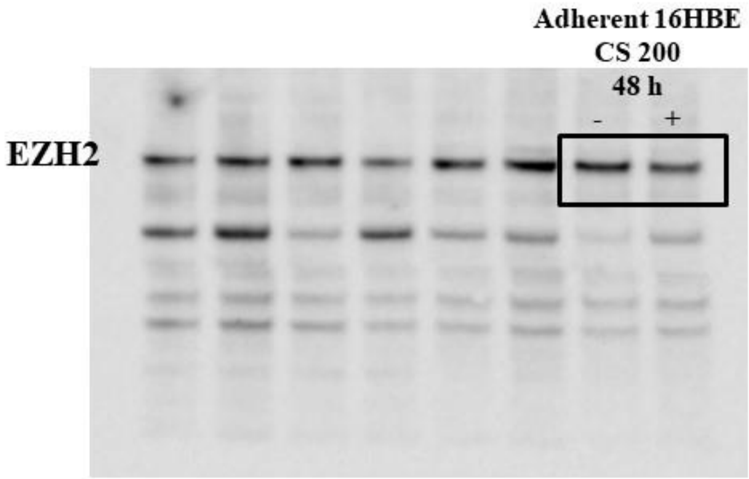

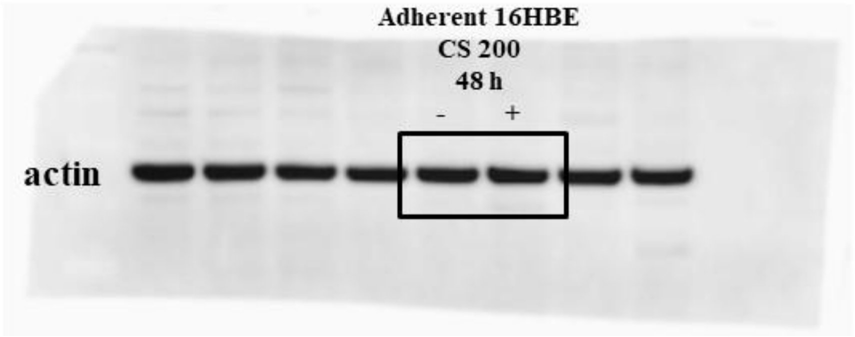

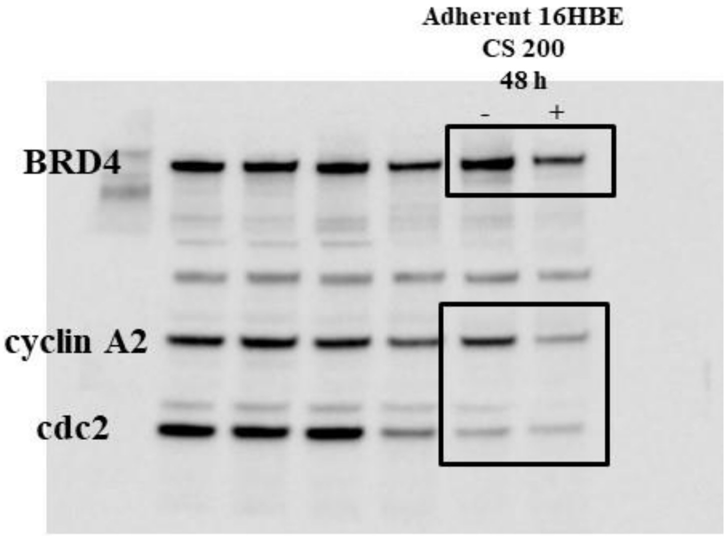

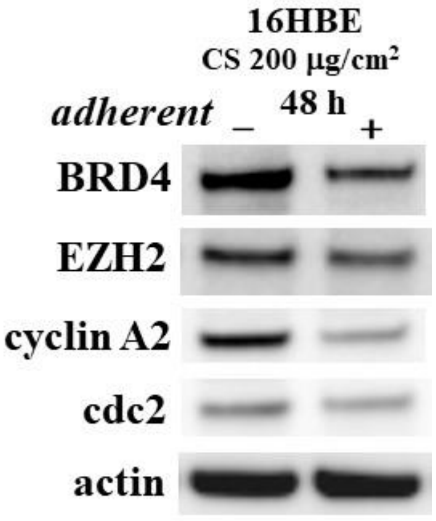

Fig 5B line 2 column 1

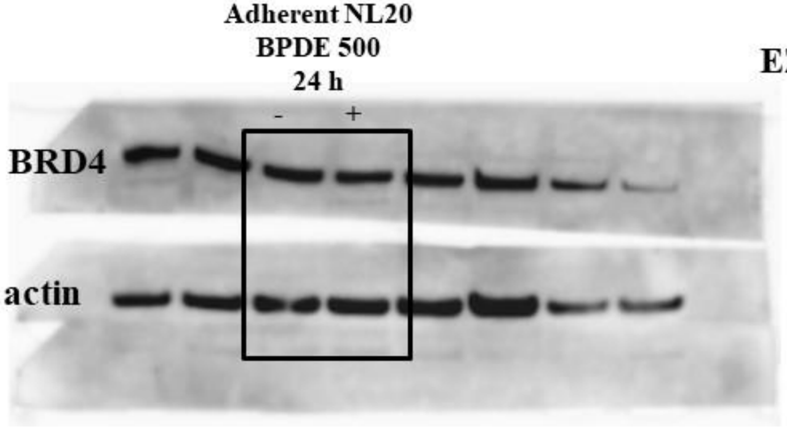

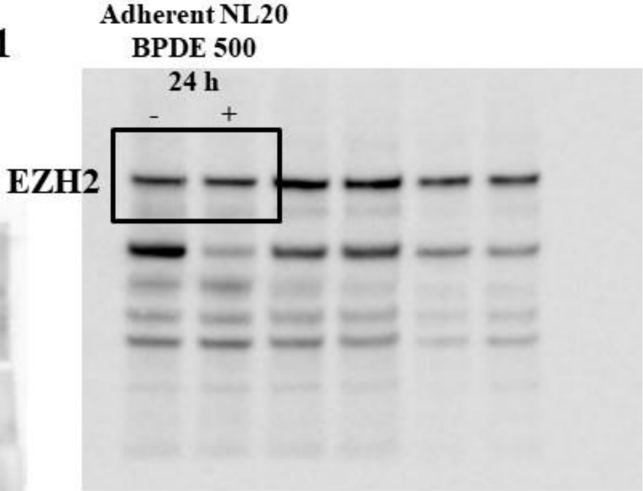

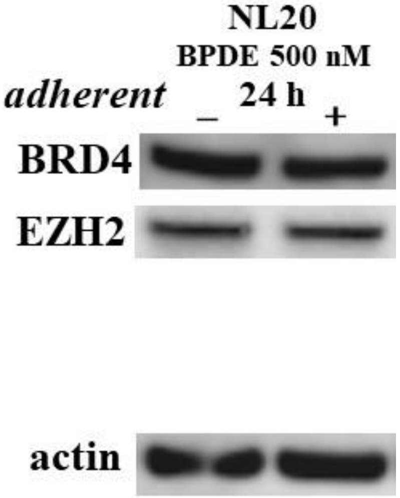

Fig 5B line 2 column 2

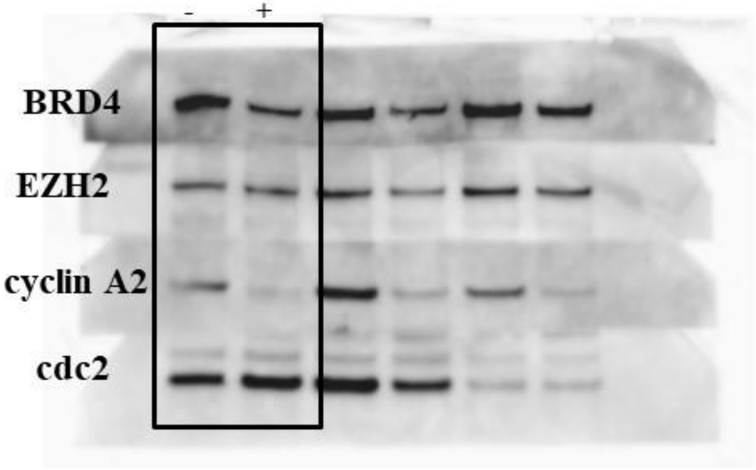

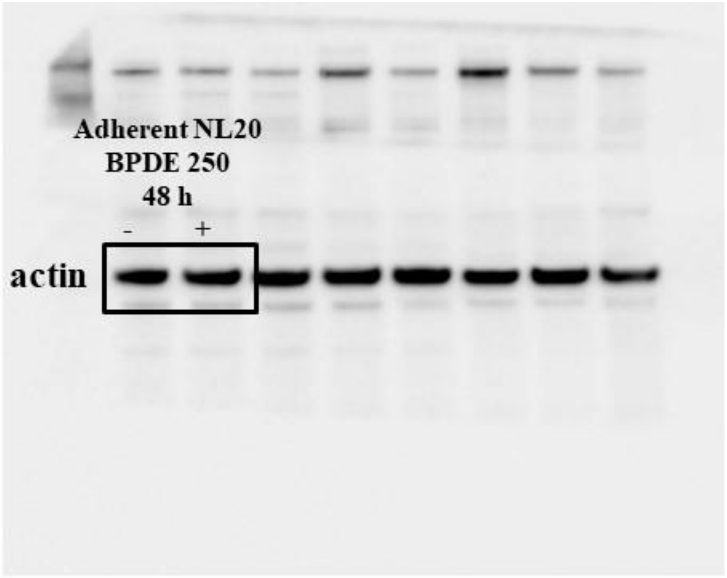

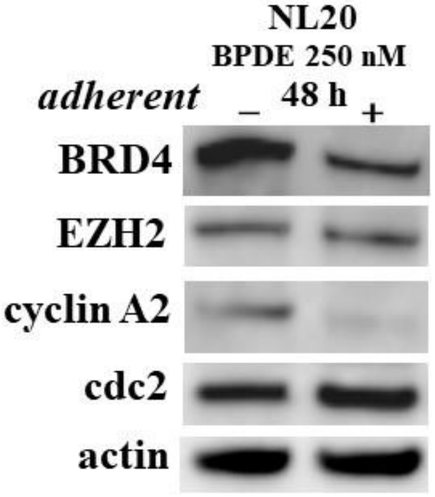

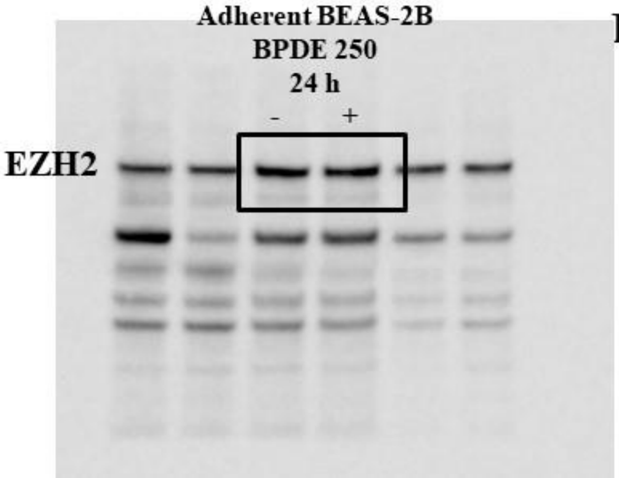

Fig 5B line 2 column 3

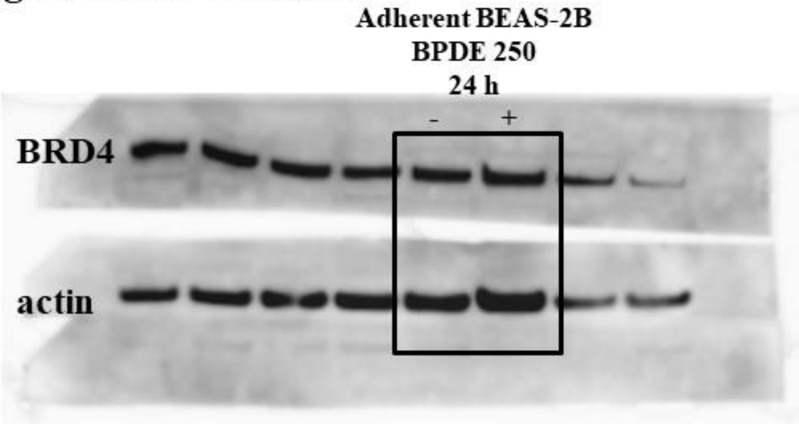

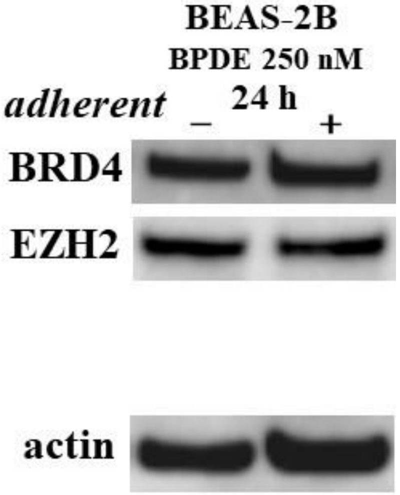

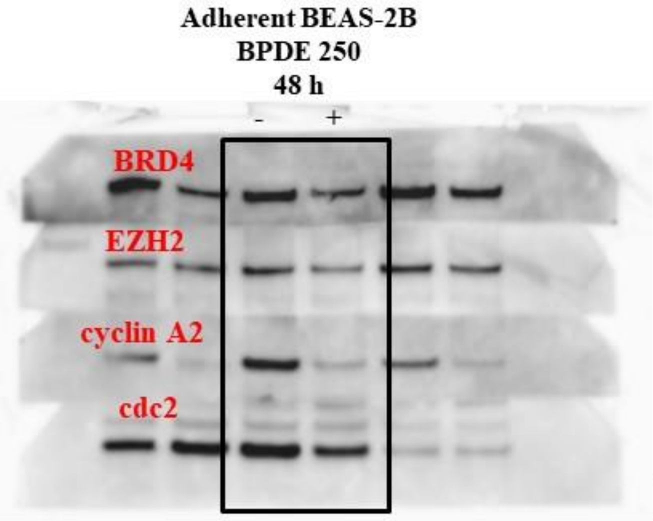

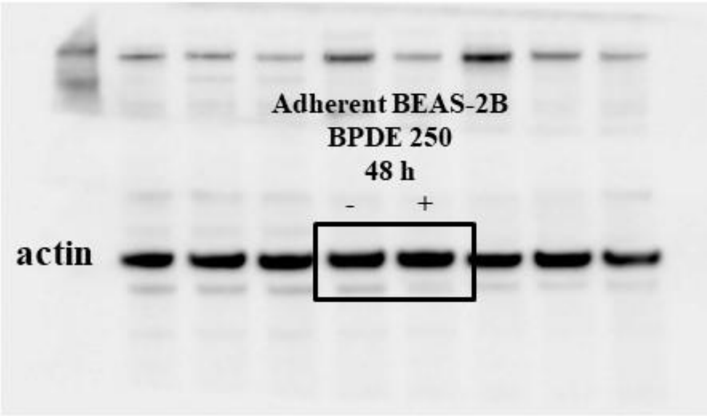

Fig 5B line 2 column 4

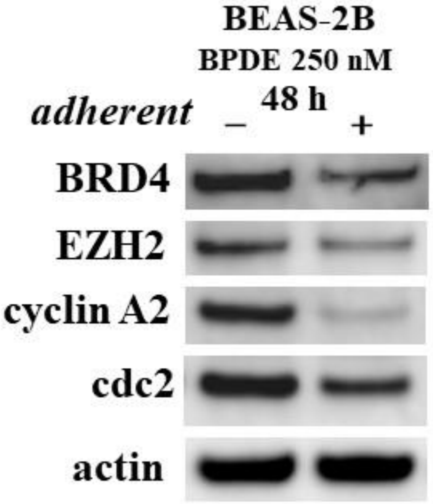

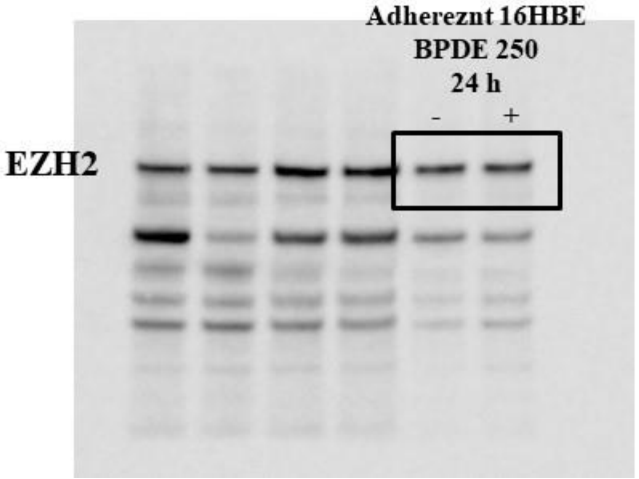

Fig 5B line 2 column 5

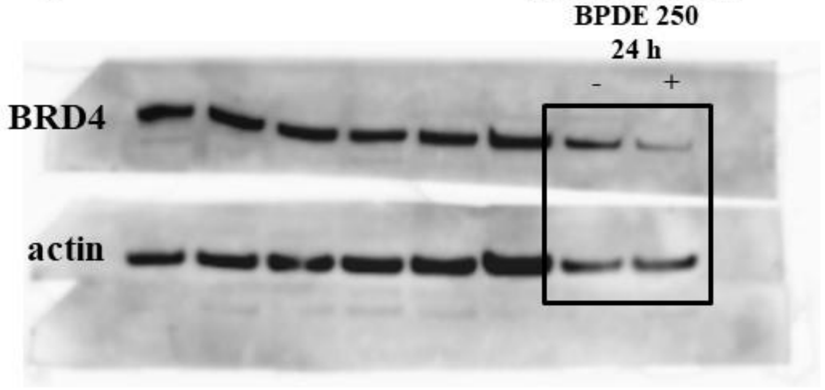

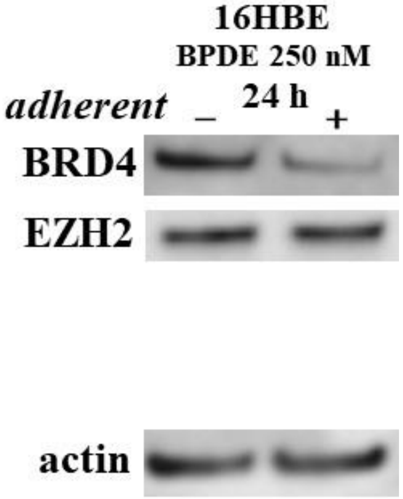

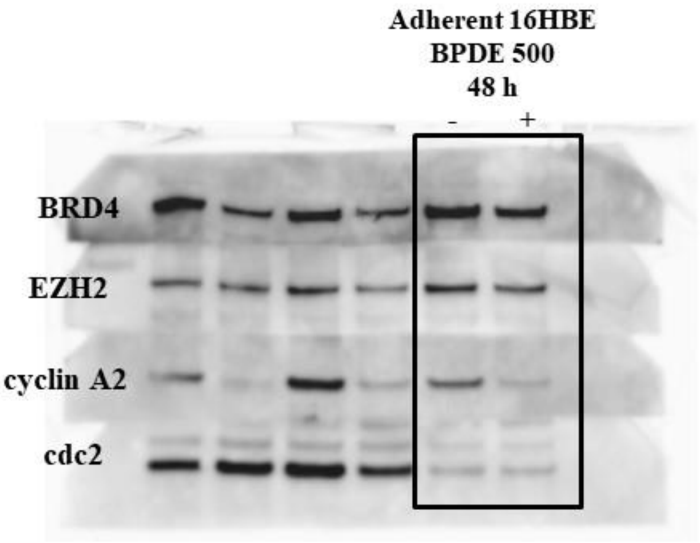

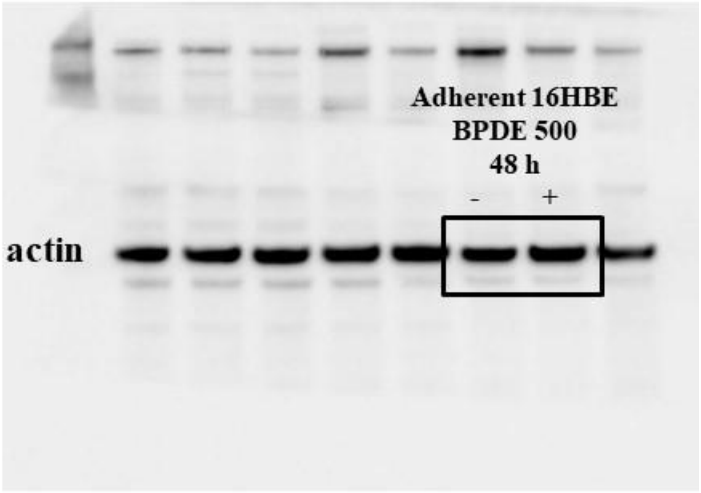

Fig 5B line 2 column 6

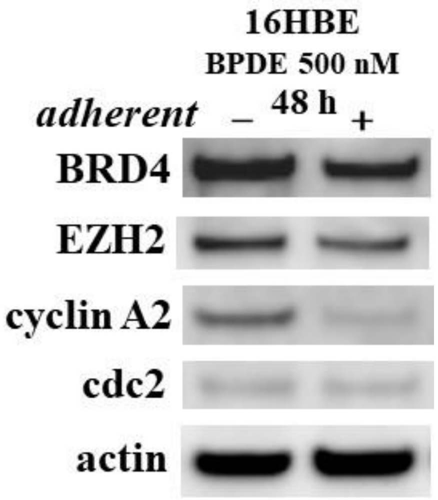

Fig 5C line 1 column 1

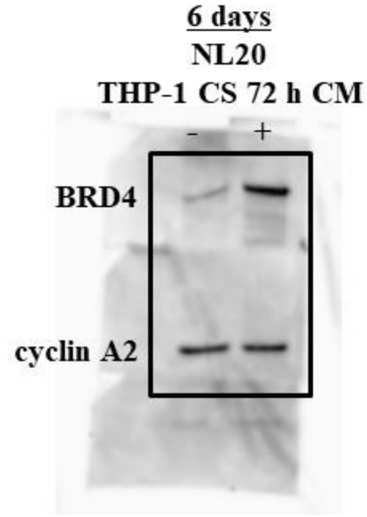

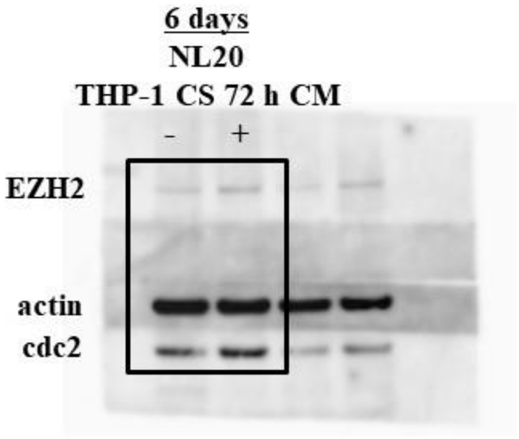

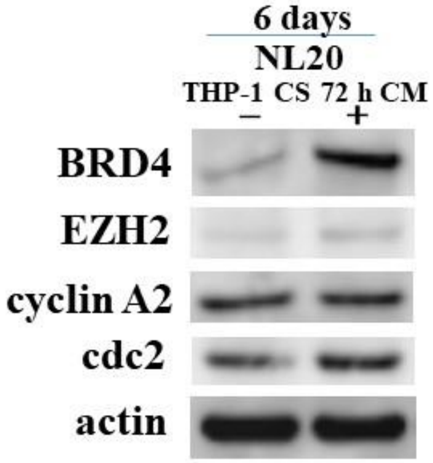

Fig 5C line 1 column 2

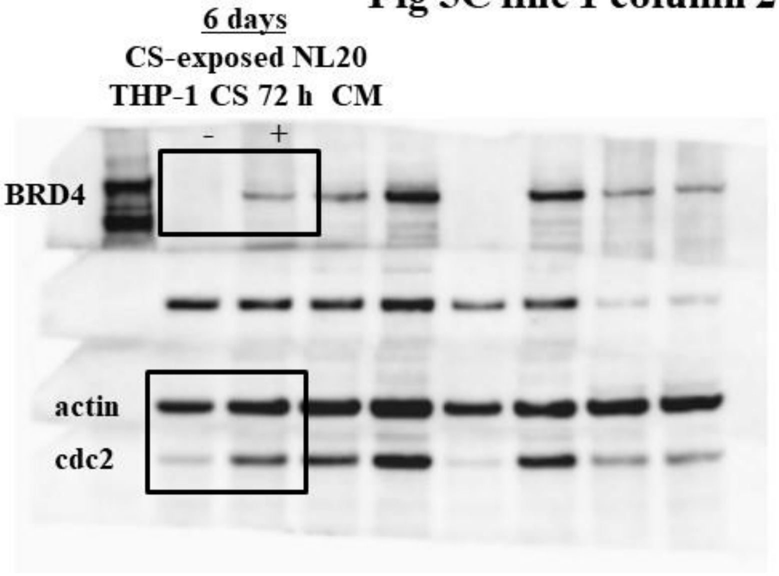

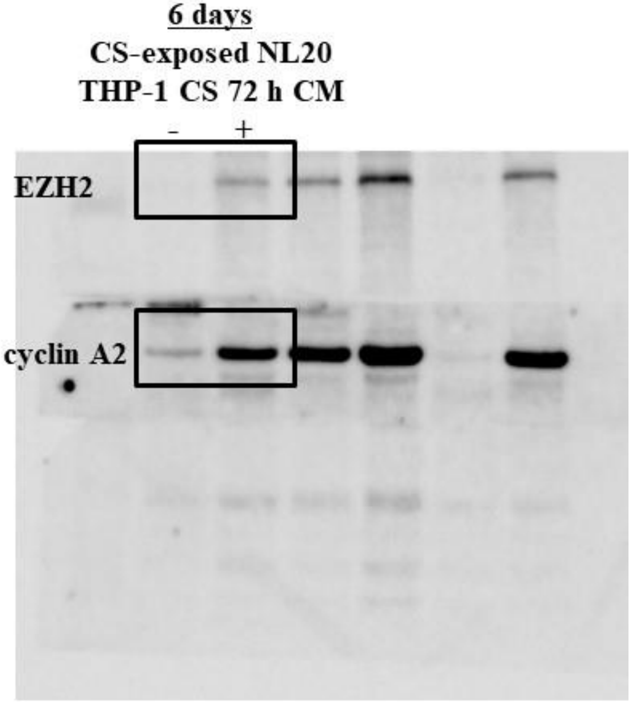

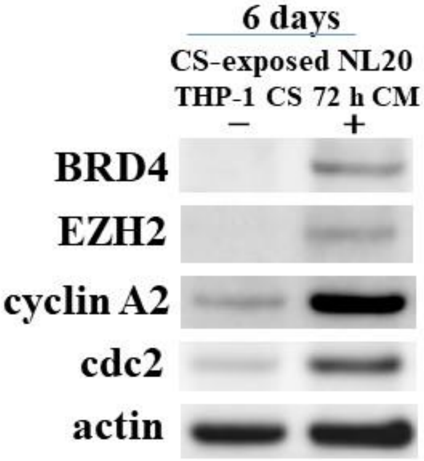

Fig 5C line 1 column 3

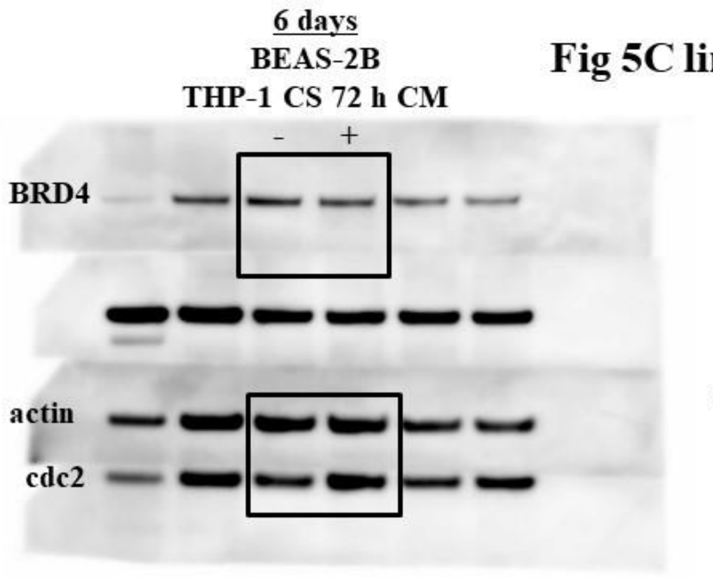

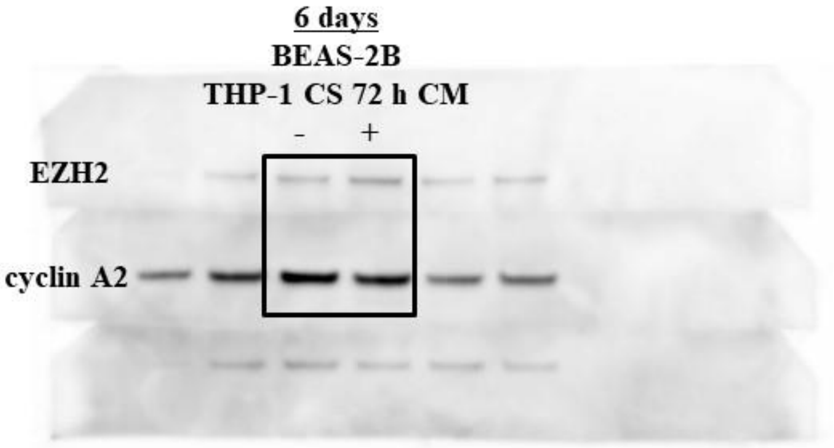

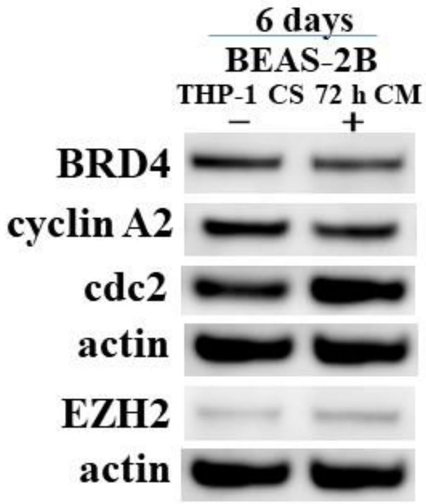

Fig 5C line 1 column 4

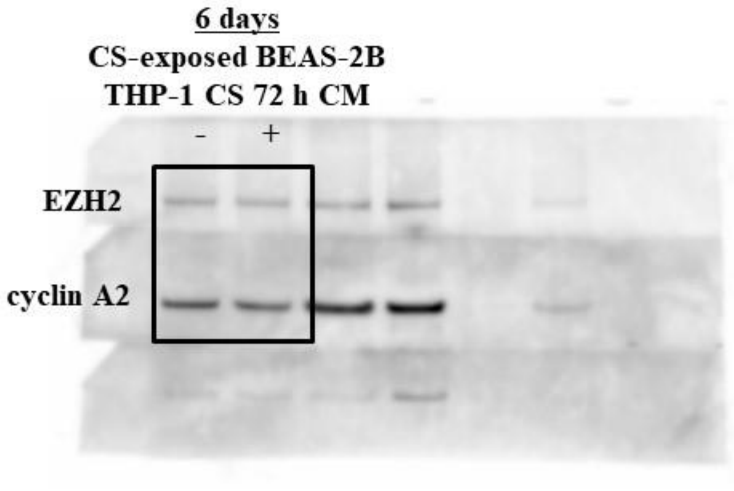

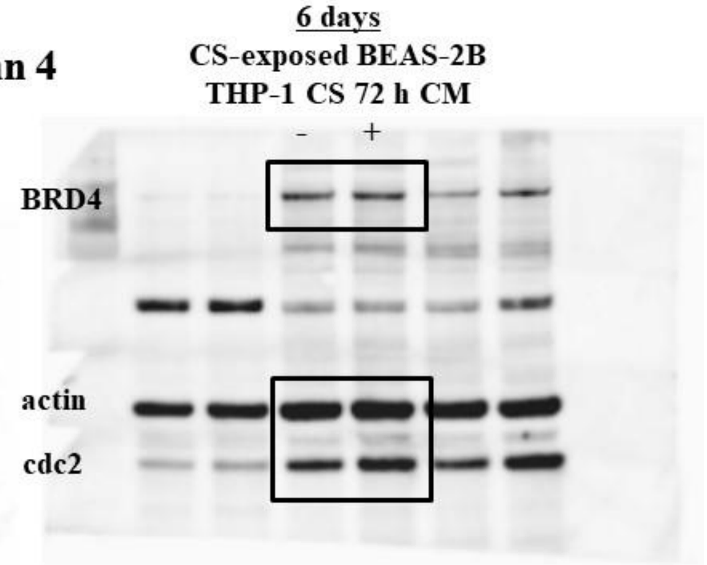

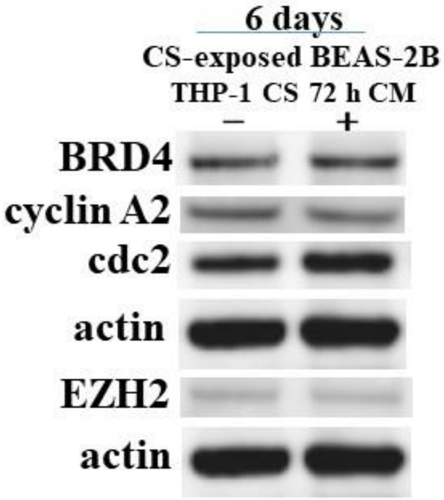

Fig 5C line 1 column 5

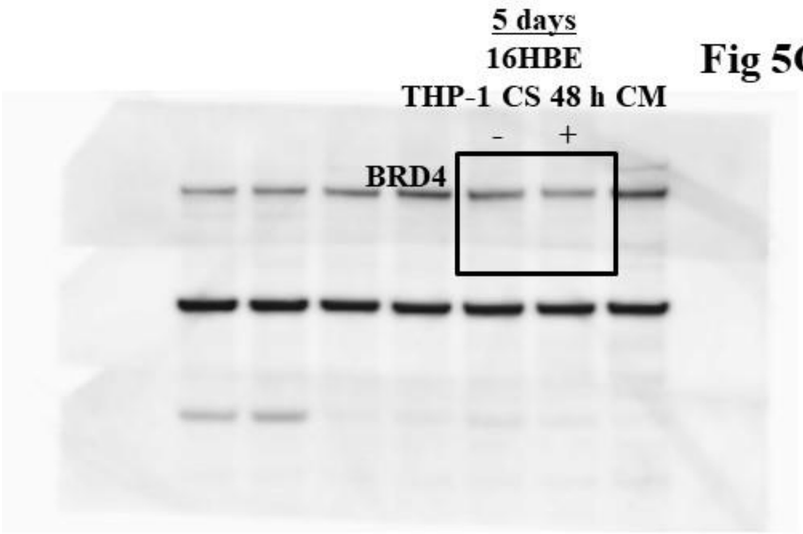

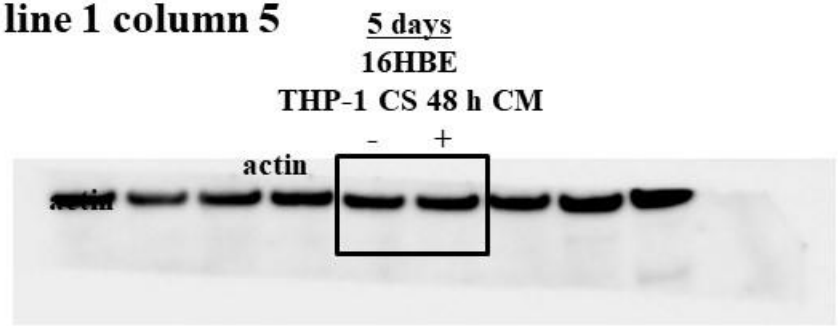

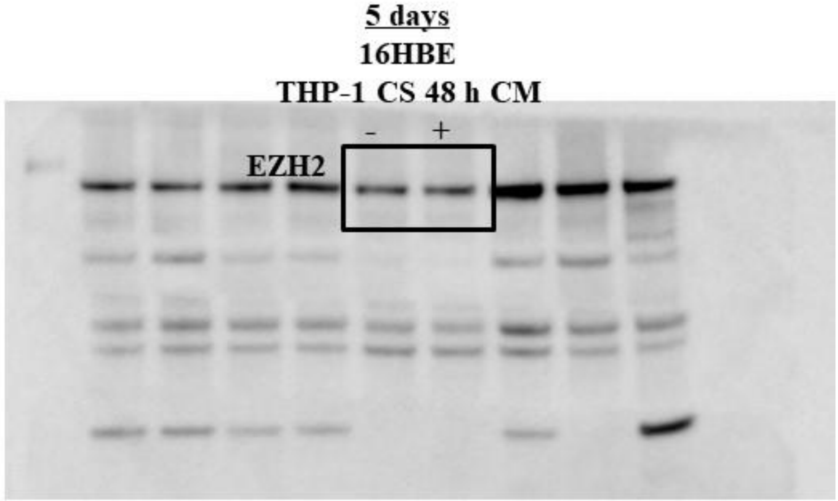

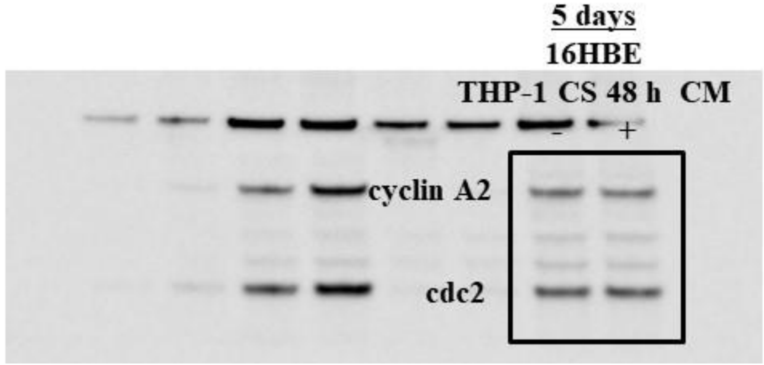

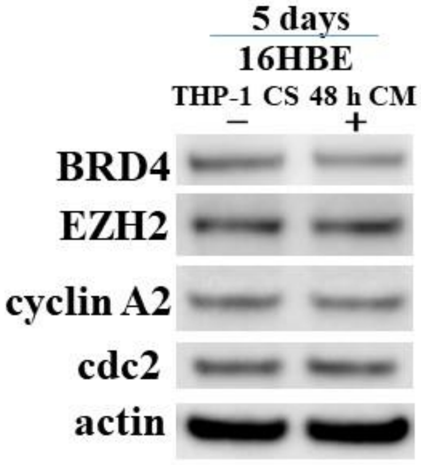

Fig 5C line 1 column 6

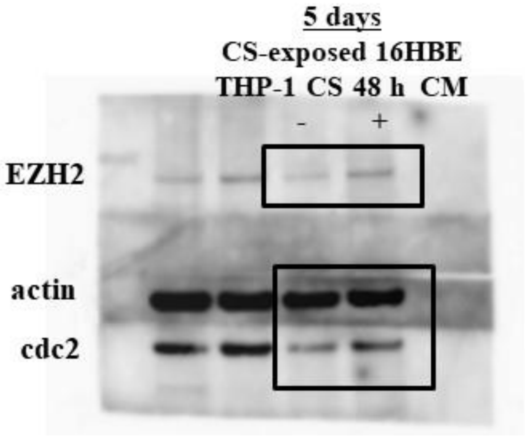

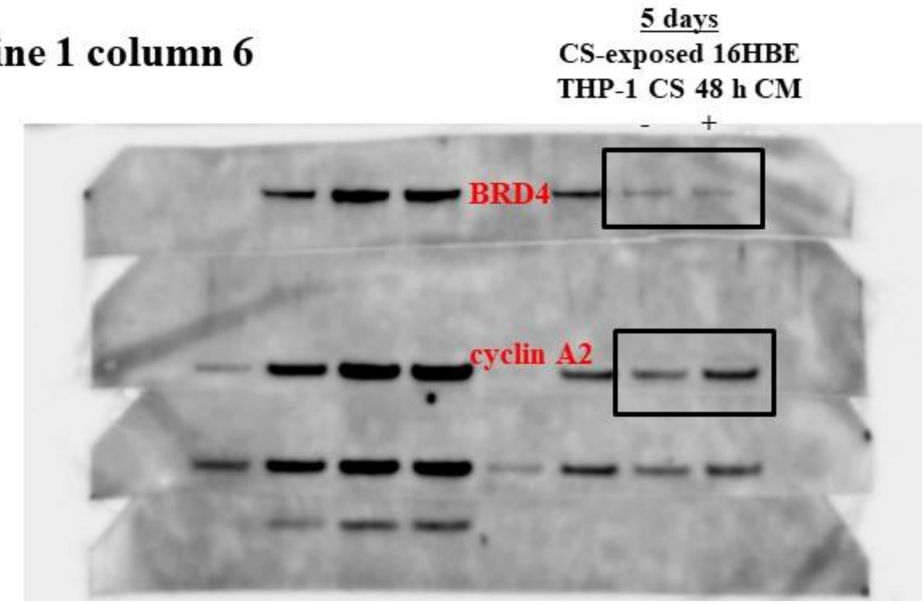

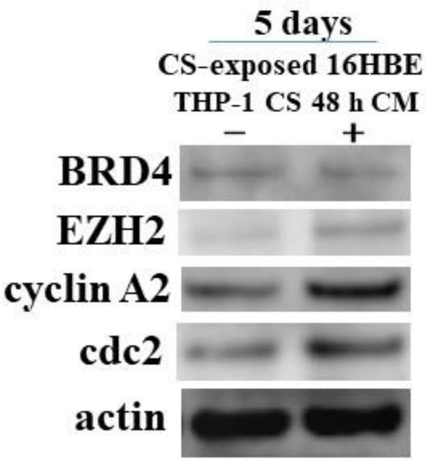

Fig 5C line 2 column 1

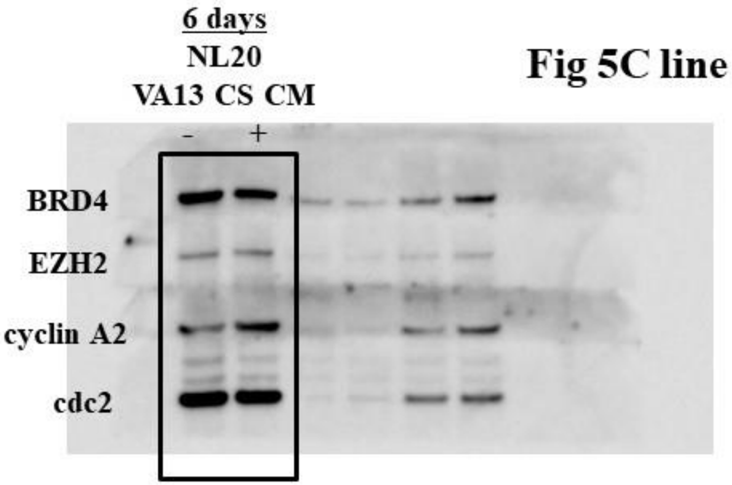

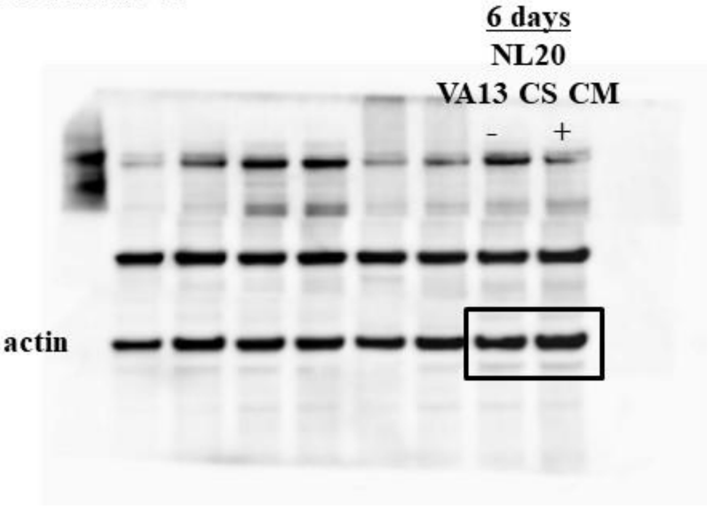

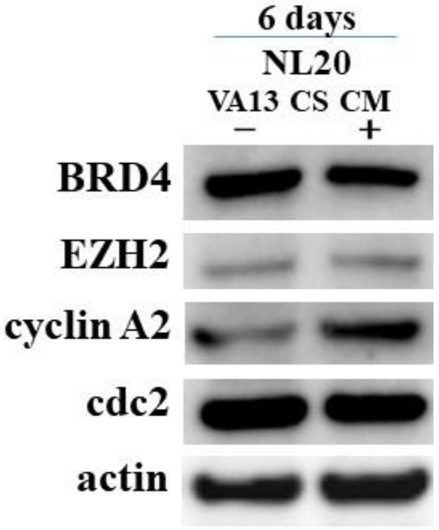

Fig 5C line 2 column 2

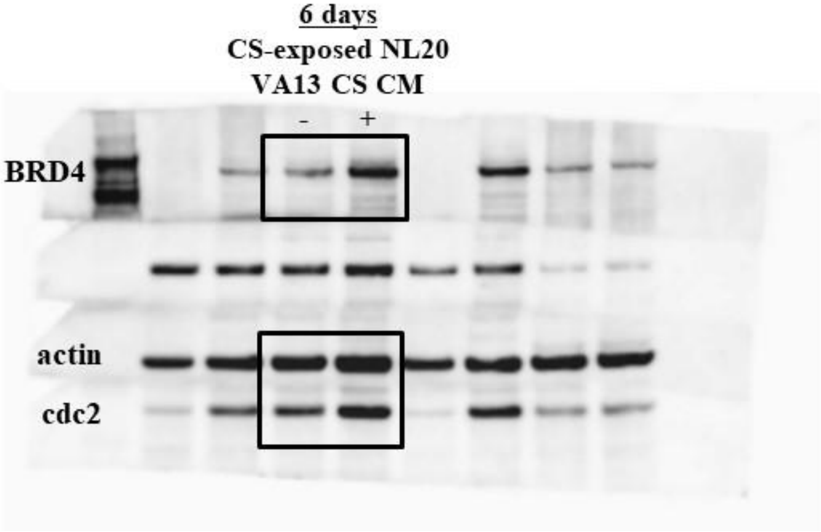

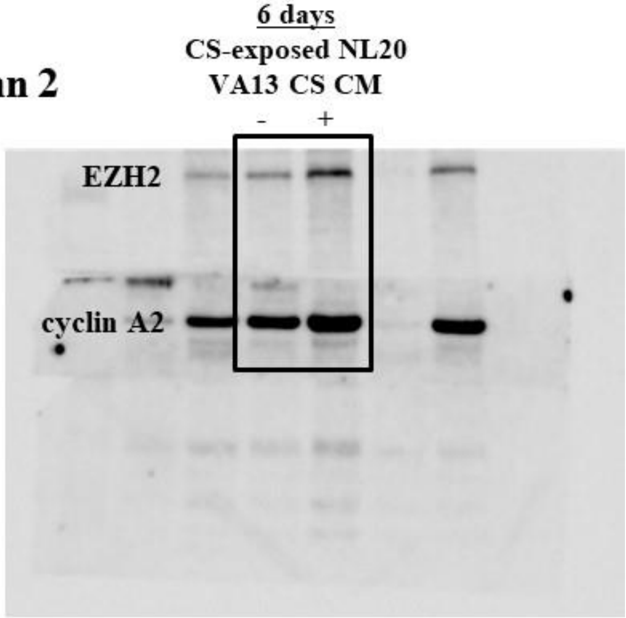

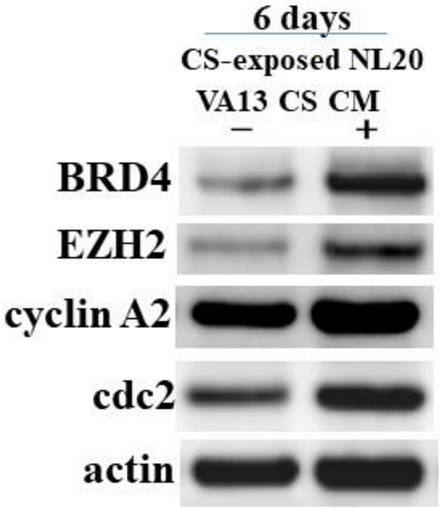

Fig 5C line 2 column 3

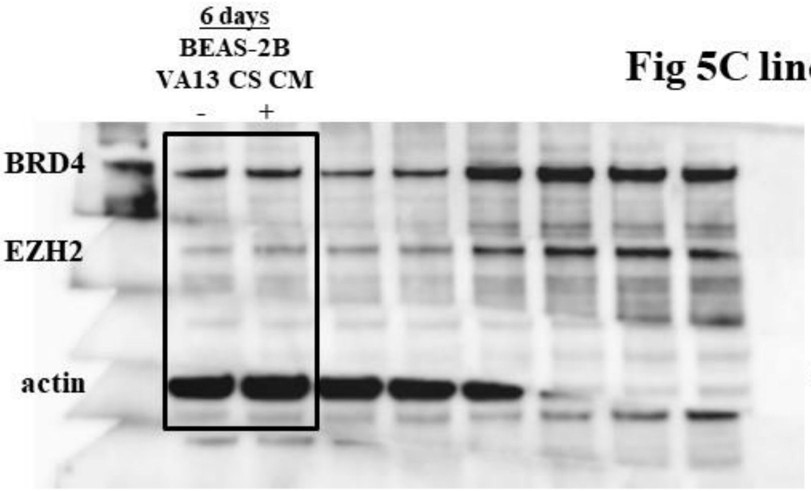

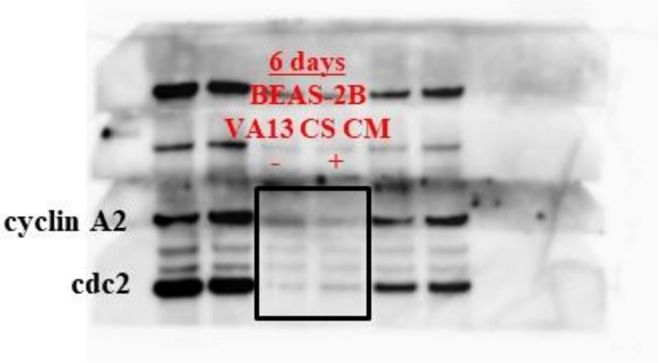

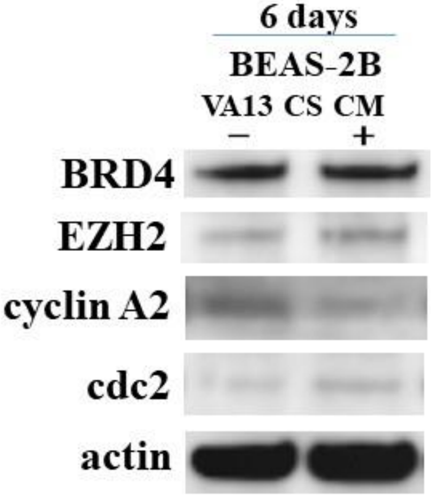

Fig 5C line 2 column 4

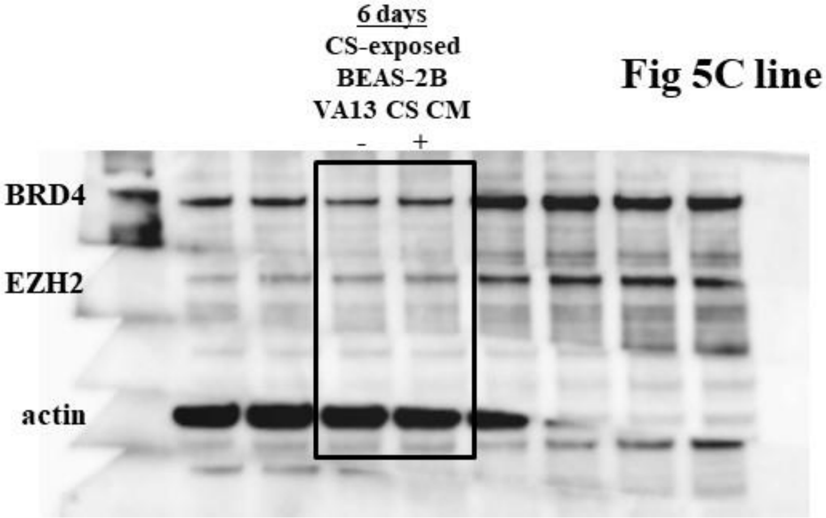

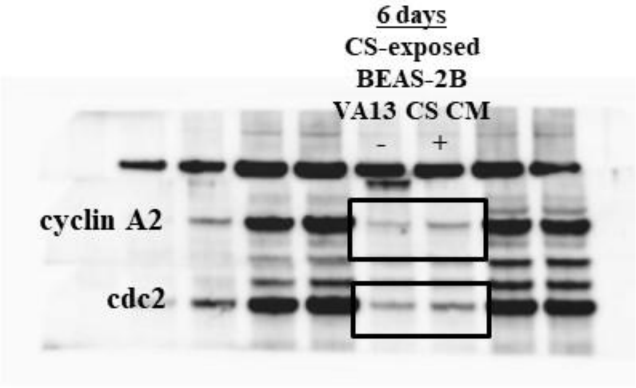

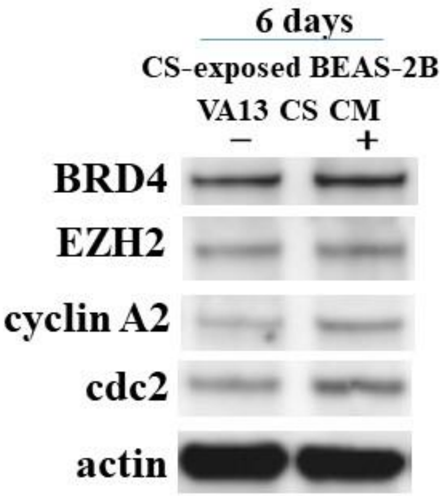

Fig 5C line 2 column 5

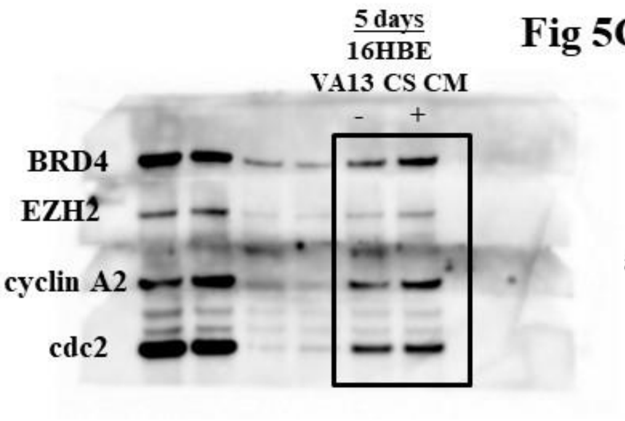

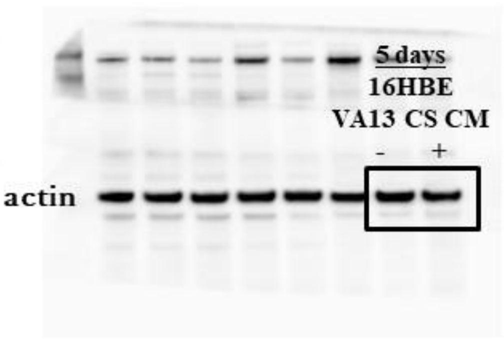

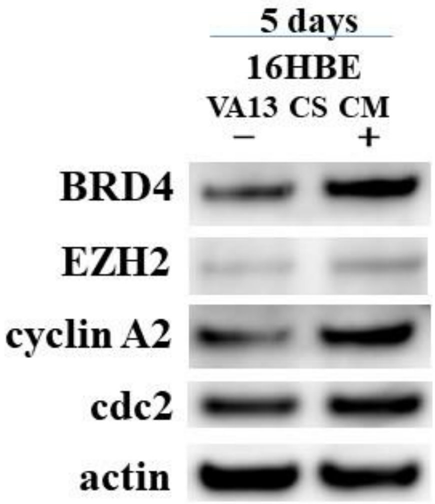

Fig 5C line 2 column 6

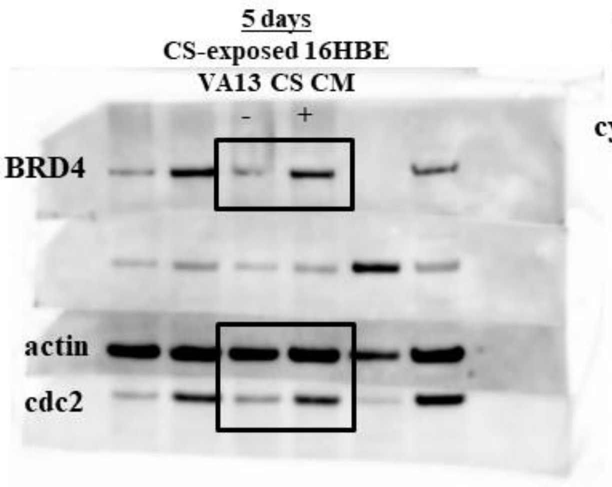

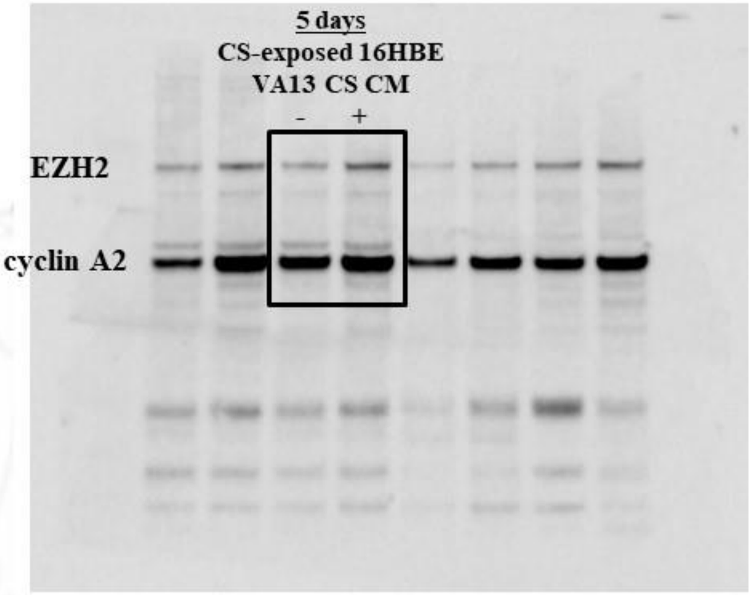

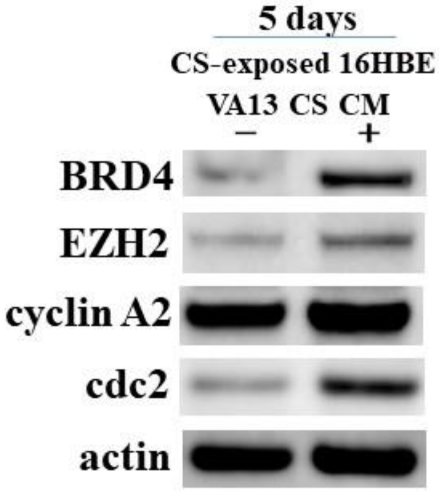

Fig 5C line 3 column 1

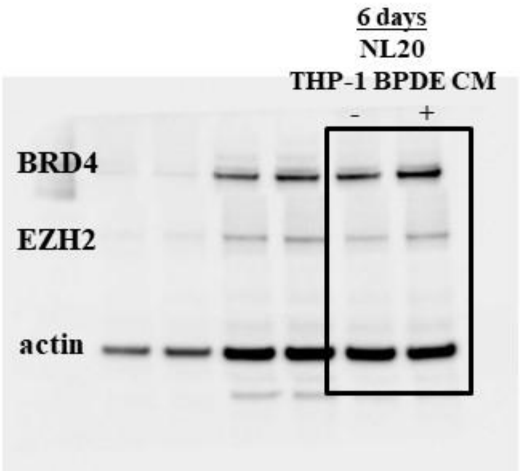

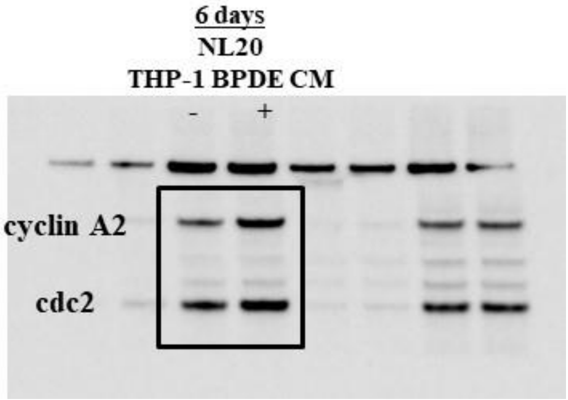

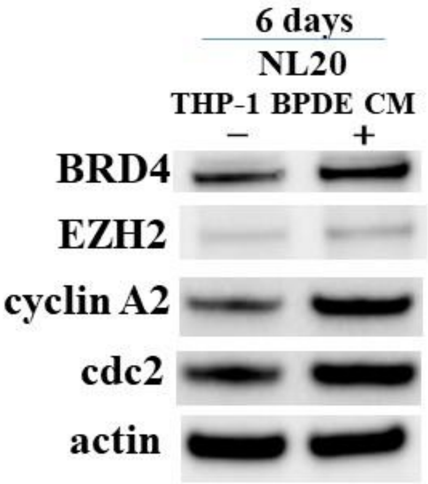

Fig 5C line 3 column 2

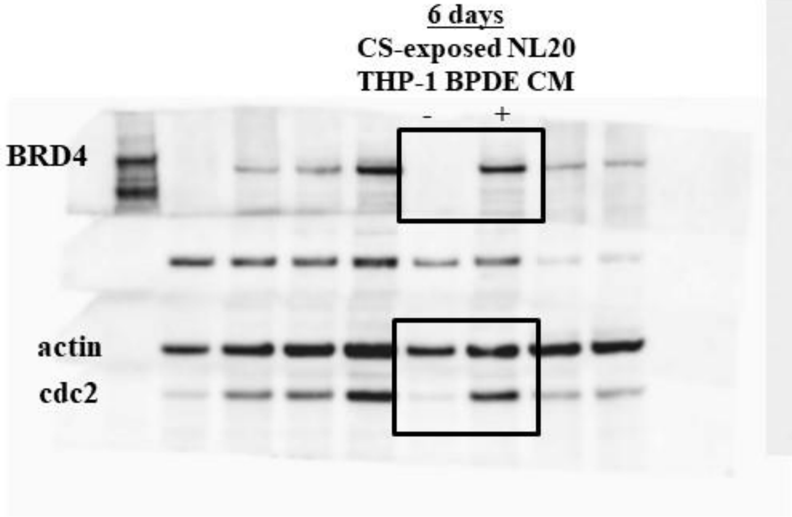

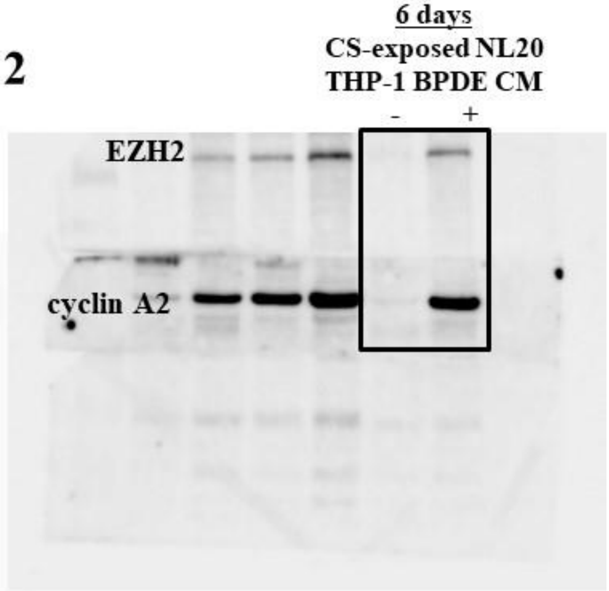

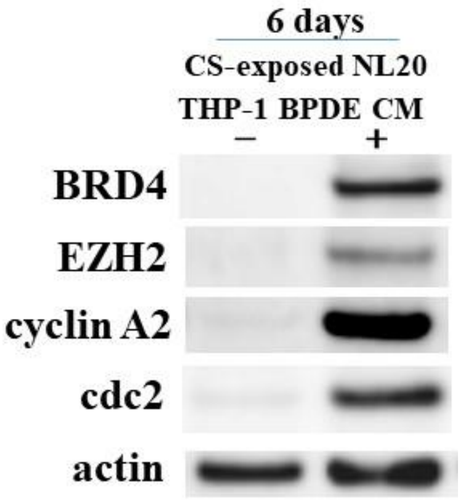

Fig 5C line 3 column 3

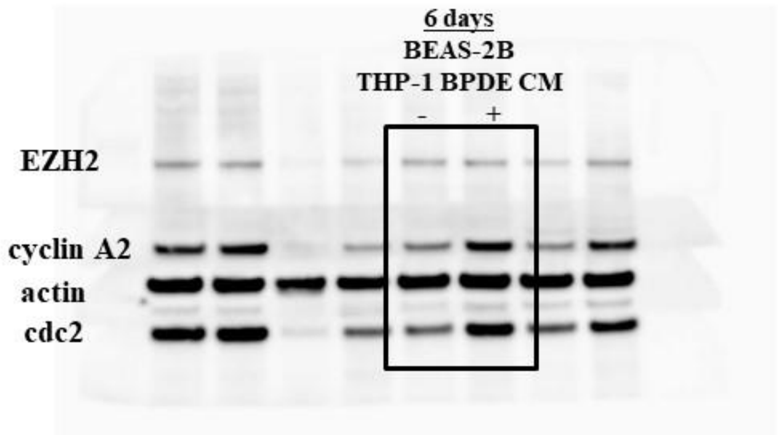

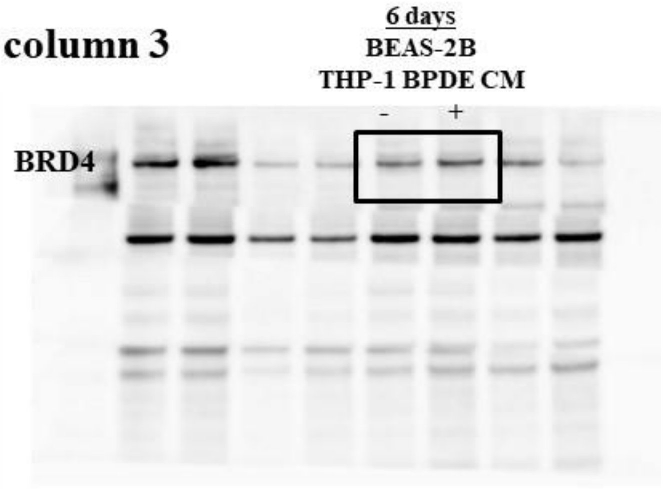

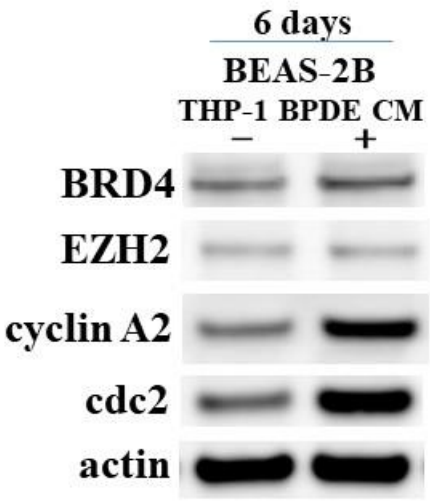

Fig 5C line 3 column 4

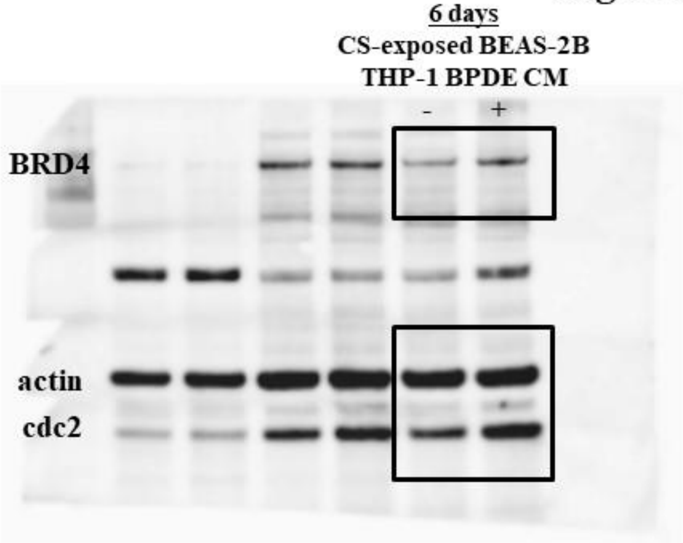

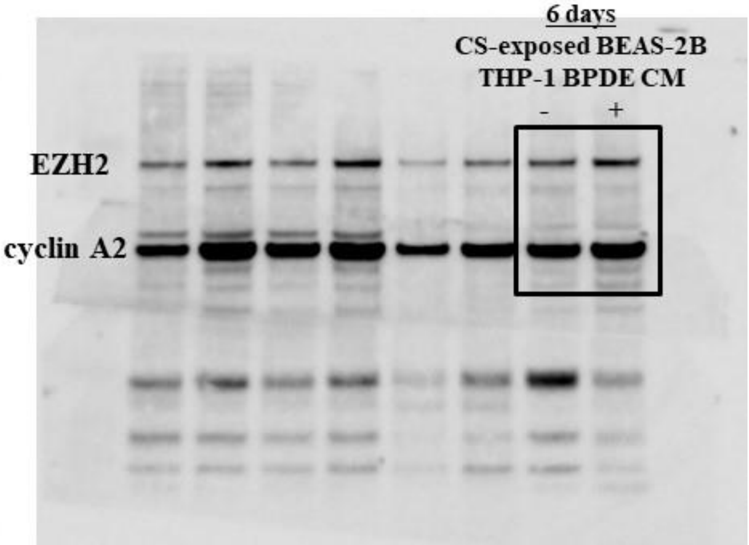

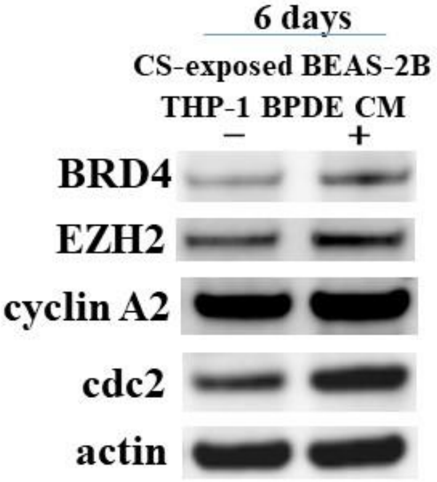

Fig 5C line 3 column 5

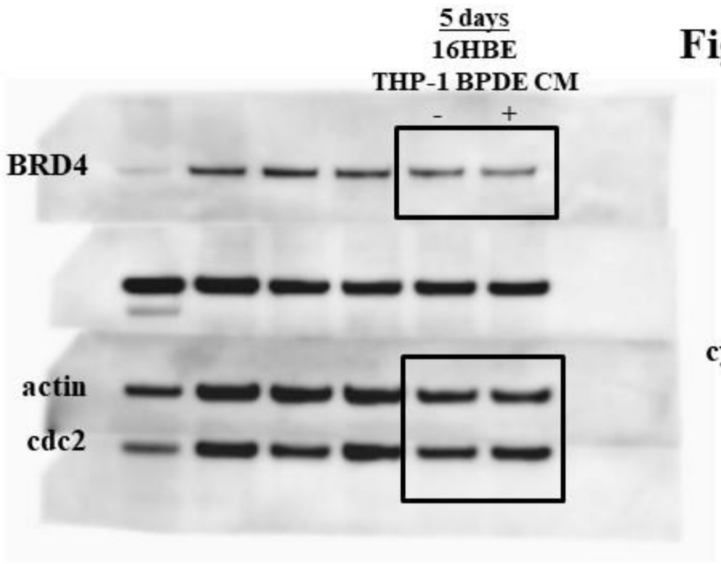

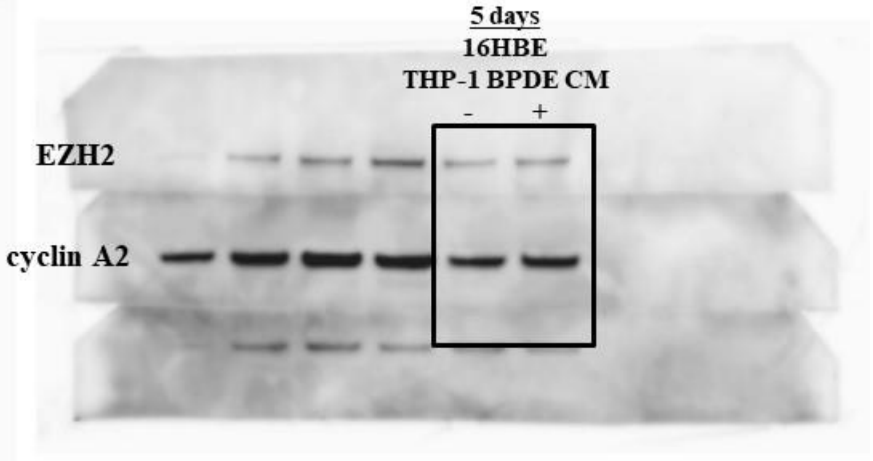

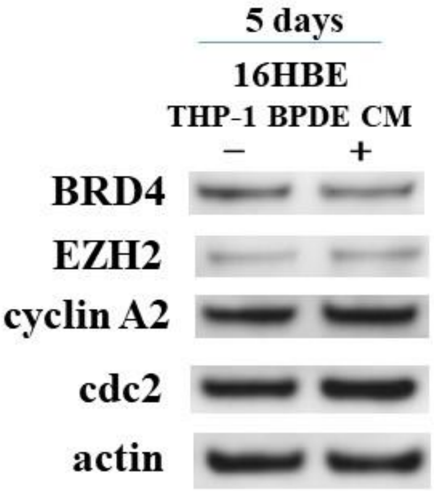

Fig 5C line 3 column 6

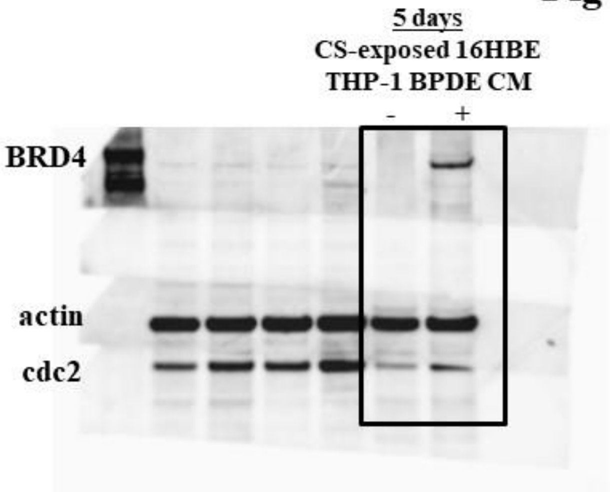

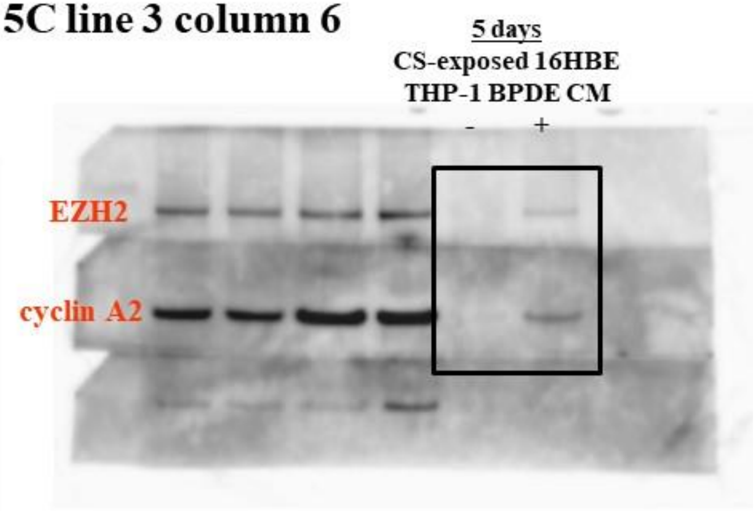

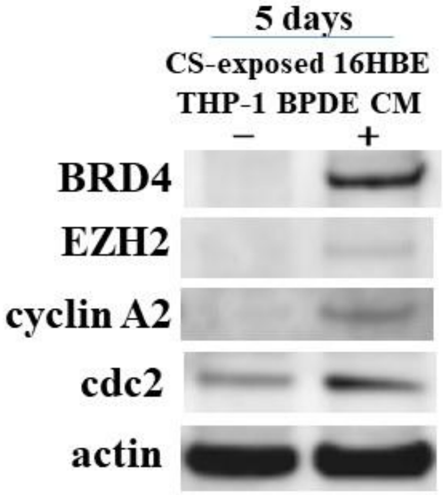

Fig 6 line 1 column 1 & line 2 column 1

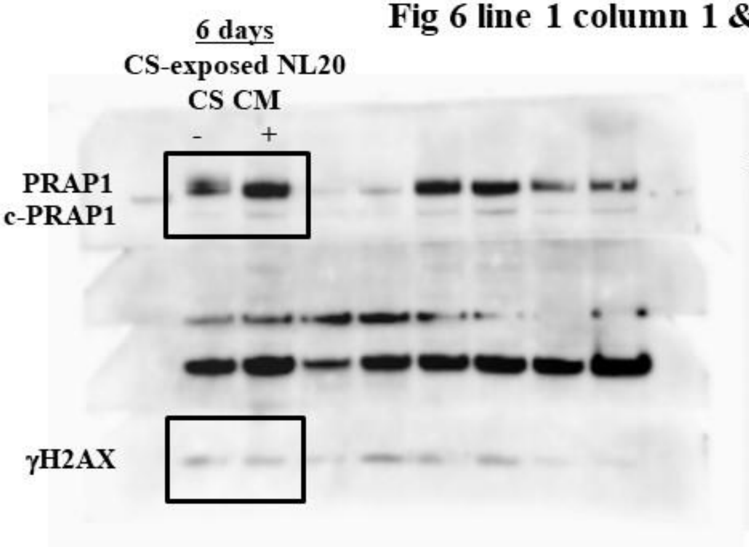

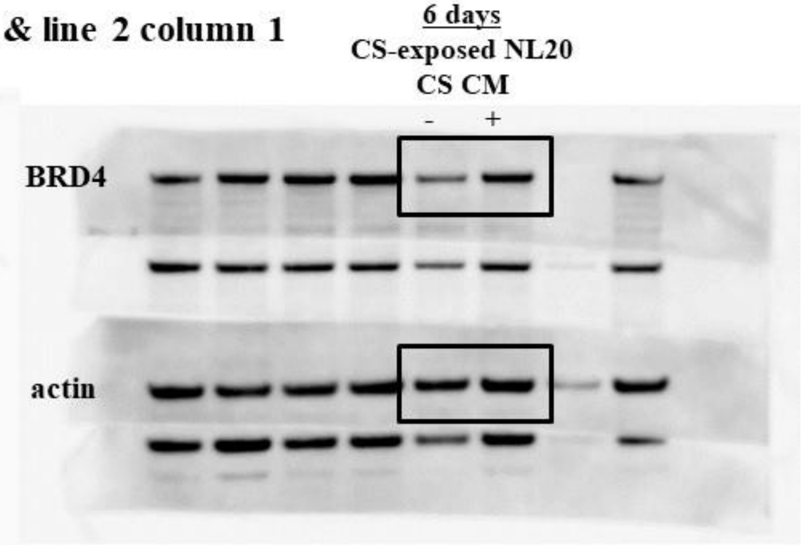

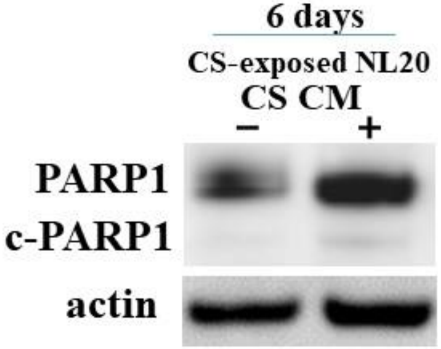

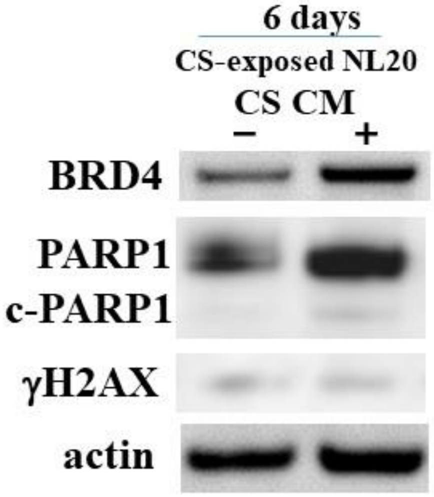

Fig 6 line 1 column 2 & line 2 column 2

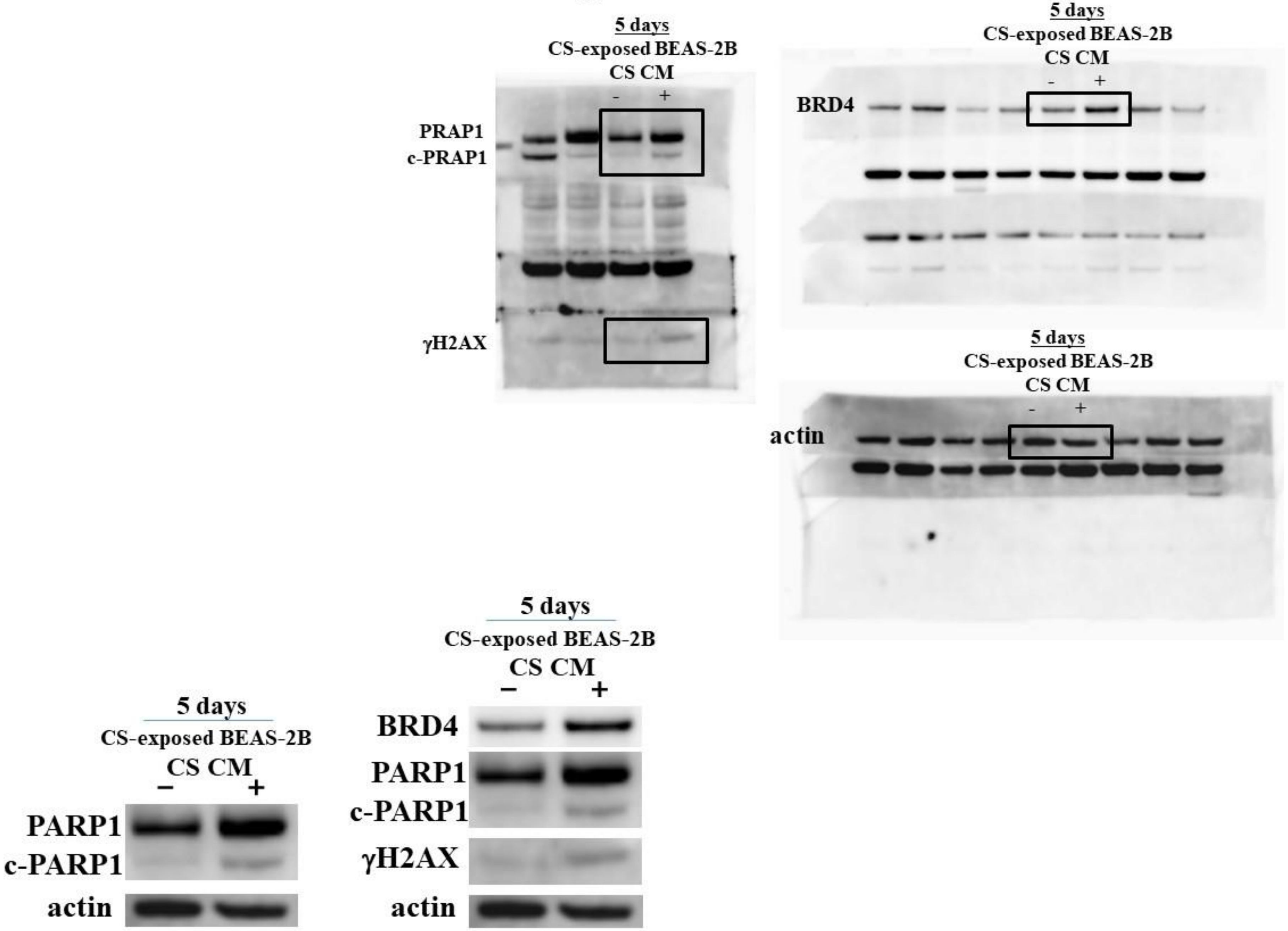

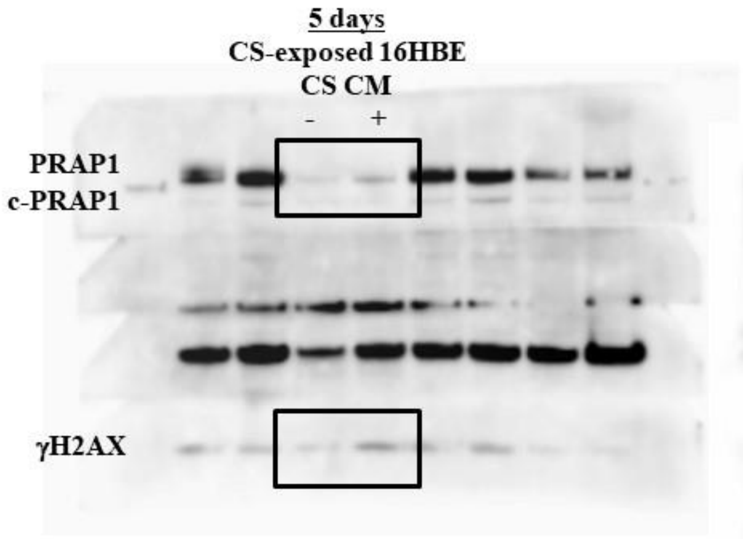

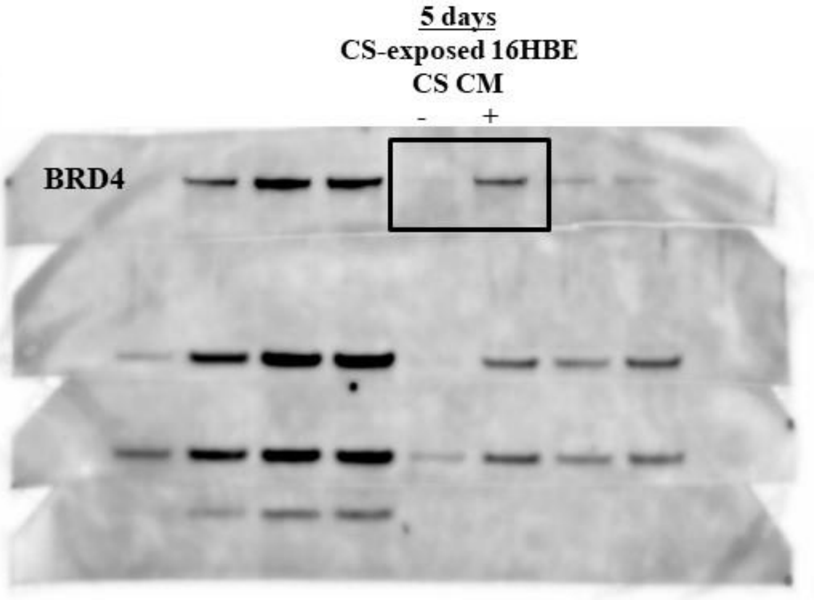

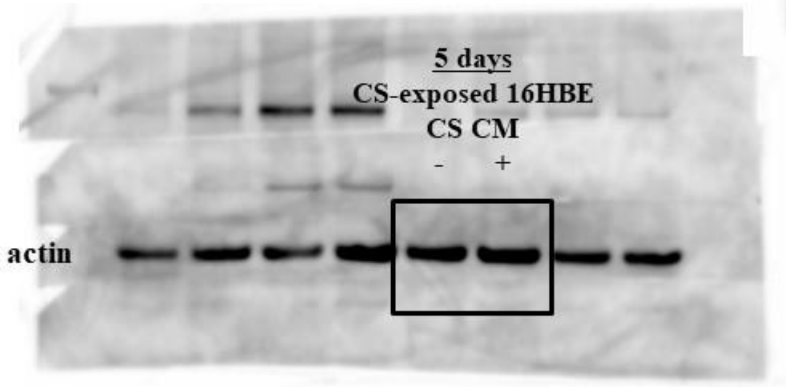

Fig 6 line1 column 3 & line 2 column 3

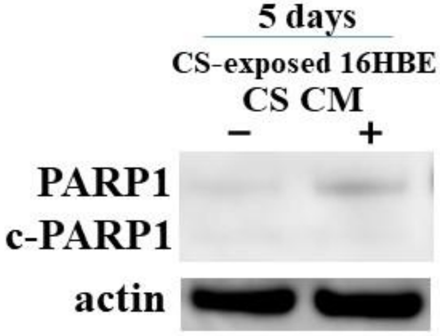

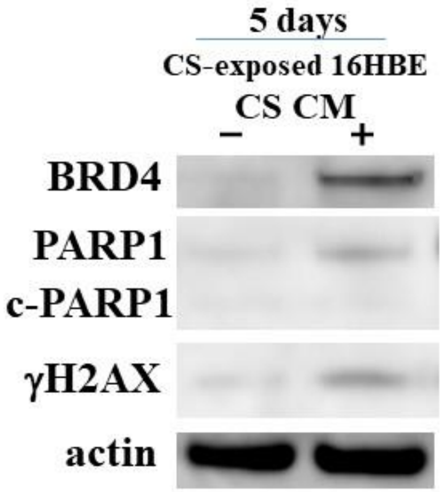

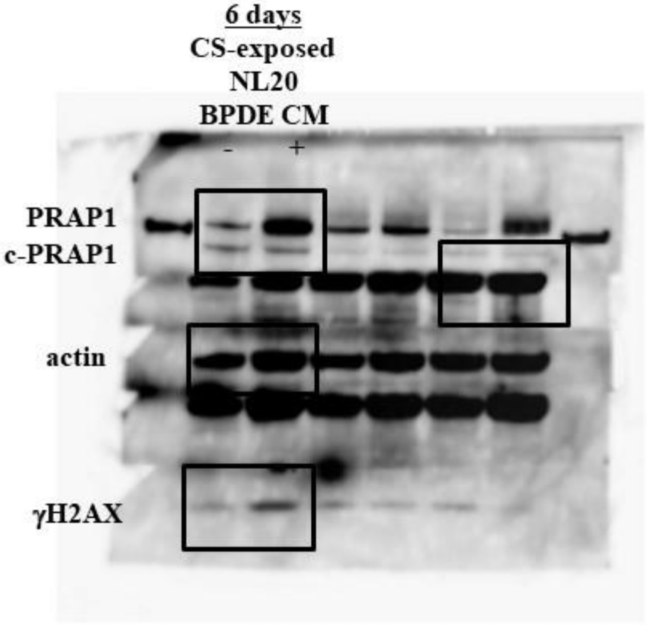

Fig 6 line 1 column 4 & line 2 column 4

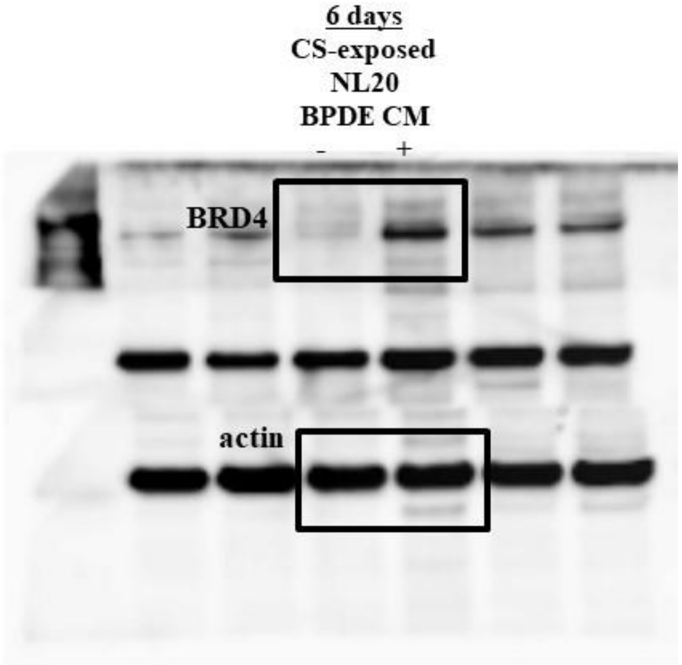

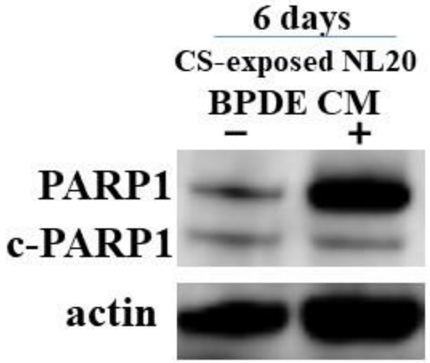

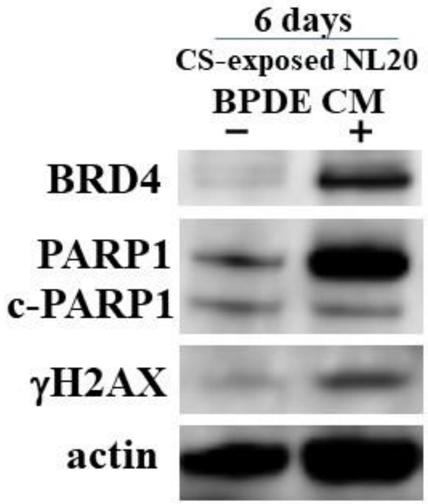

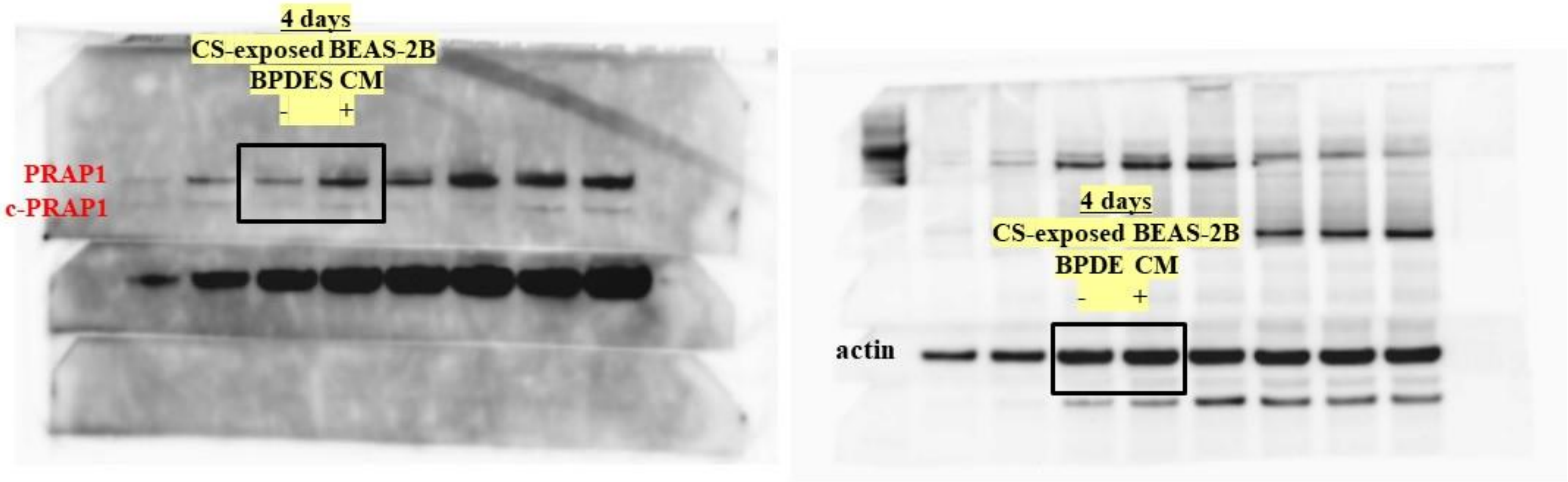

Fig 6 line 1 column 5

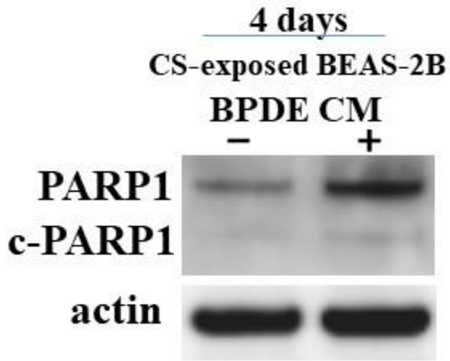

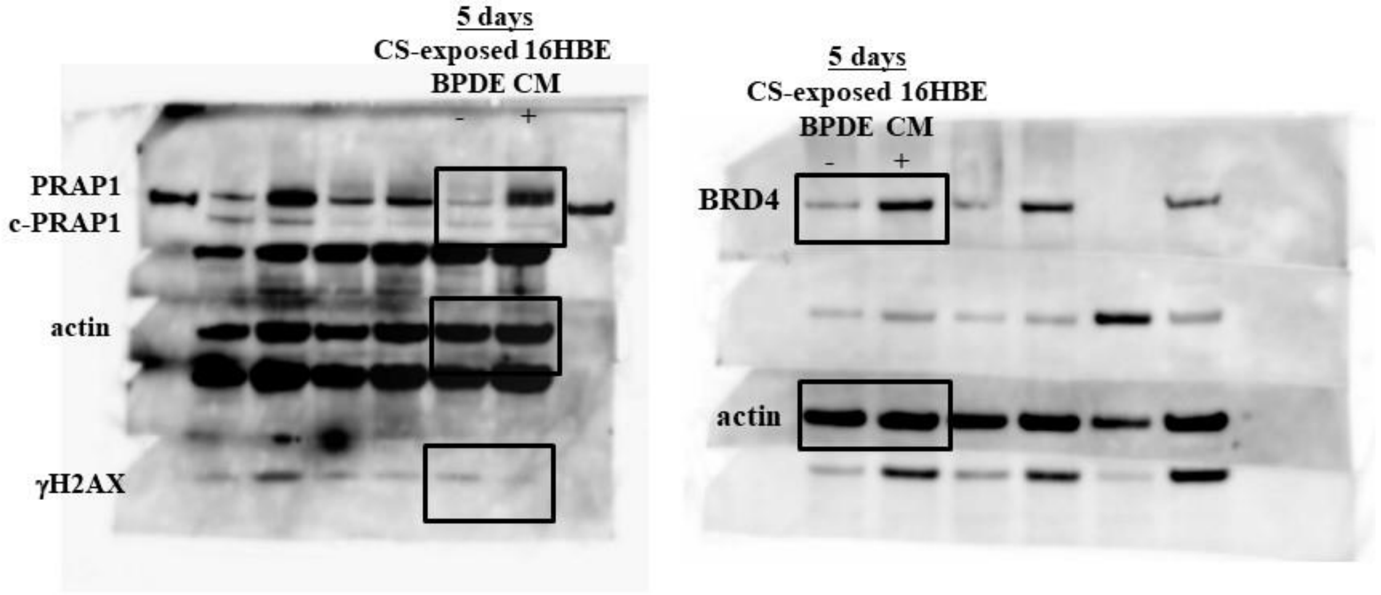

Fig 6 line 1 column 6 & line 2 column 5

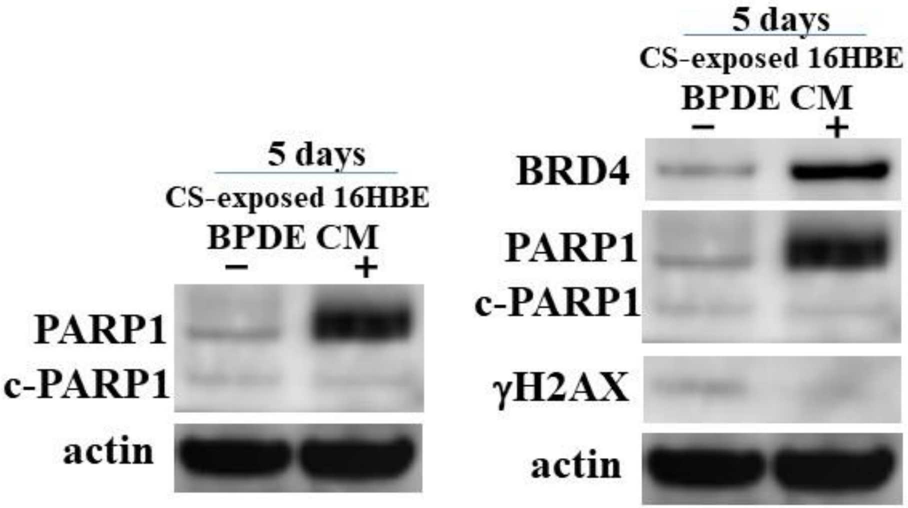

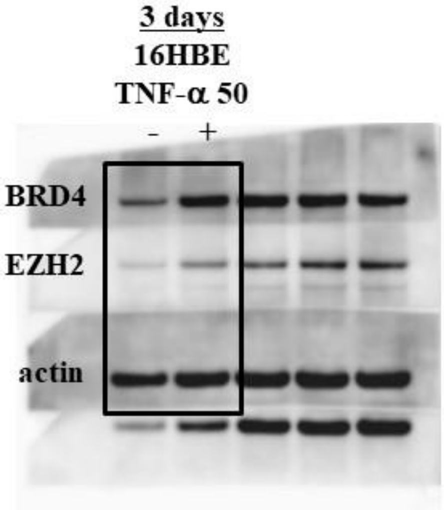

Fig 7C line 1 column 1

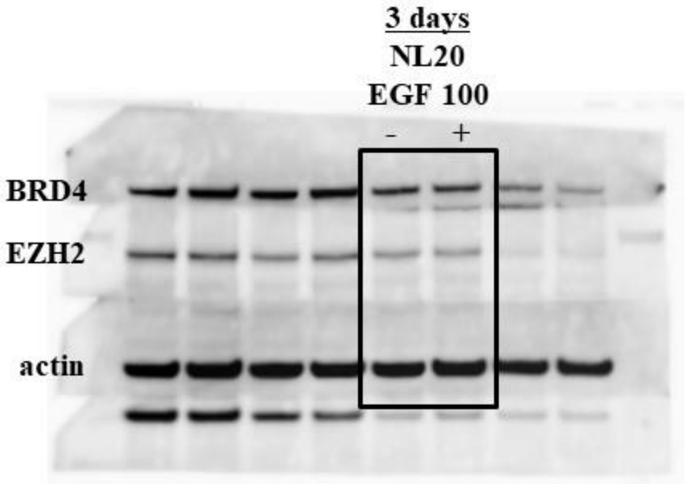

Fig 7C line 1 column 2

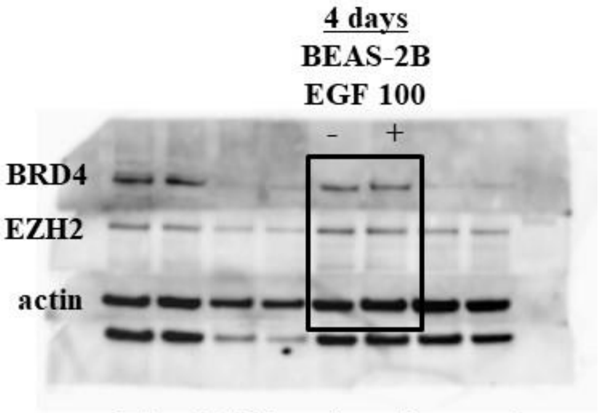

Fig 7C line 1 column 3

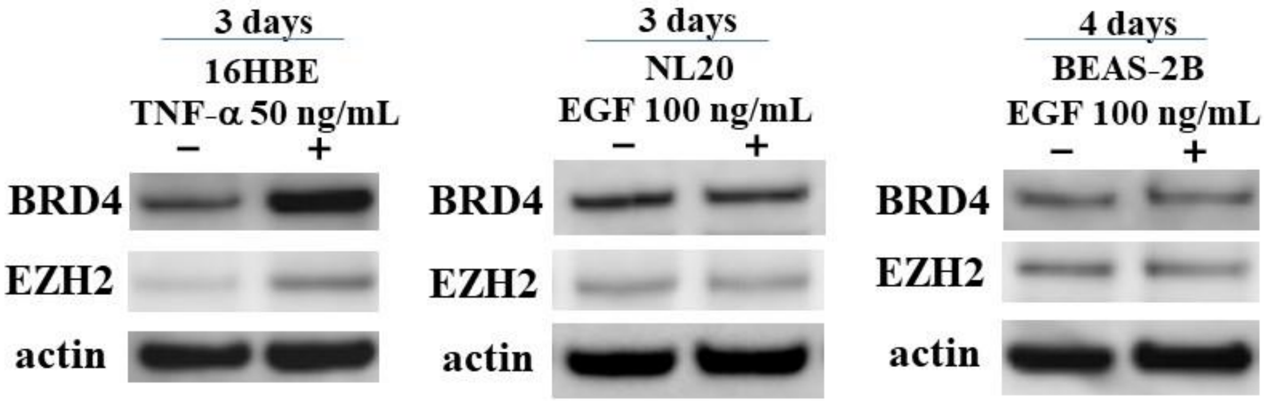

Supplement: S1 Raw images — (PDF) [file pone.0285354.s001.pdf]
