# Supplementary material for: Crystalline silica-exposed human lung epithelial cells presented enhanced anchorage-independent growth with upregulated expression of BRD4 and EZH2 in autocrine and paracrine manners
Source: PLoS One. 2023 May 5;18(5):e0285354. doi: 10.1371/journal.pone.0285354 (PMC10162546; doi:10.1371/journal.pone.0285354)
Supplement: Attachment — Submitted filename: Response to reviewers.docx [file pone.0285354.s002.docx]

**Responses to the comments made by Academic Editor**

Dear Dr. Rabilloud:

We wish to express our sincere appreciation for your valuable comments on our manuscript. We acknowledge that the comments have helped us significantly improve the manuscript for consideration in your esteemed journal. Our responses to your comments are given below.

**Comment 1:** Please ensure that your manuscript meets PLOS ONE's style requirements, including those for file naming. The PLOS ONE style templates can be found at

<https://journals.plos.org/plosone/s/file?id=wjVg/PLOSOne_formatting_sample_main_body.pdf> and

<https://journals.plos.org/plosone/s/file?id=ba62/PLOSOne_formatting_sample_title_authors_affiliations.pdf>

***Response:*** *As suggested, we have ensured that the manuscript conforms to the PLOS ONE style guidelines.*

**Comment 2:** PLOS ONE now requires that authors provide the original uncropped and unadjusted images underlying all blot or gel results reported in a submission’s figures or Supporting Information files. This policy and the journal’s other requirements for blot/gel reporting and figure preparation are described in detail at https://journals.plos.org/plosone/s/figures#loc-blot-and-gel-reporting-requirements and [https://journals.plos.org/plosone/s/figur:s#loc-preparing-figures-from-image-files](https://journals.plos.org/plosone/s/figures#loc-preparing-figures-from-image-files). When you submit your revised manuscript, please ensure that your figures adhere fully to these guidelines and provide the original underlying images for all blot or gel data reported in your submission. See the following link for instructions on providing the original image data: <https://journals.plos.org/plosone/s/figures#loc-original-images-for-blots-and-gels>.

In your cover letter, please note whether your blot/gel image data are in Supporting Information or posted at a public data repository, provide the repository URL if relevant, and provide specific details as to which raw blot/gel images, if any, are not available. Email us at plosone@plos.org if you have any questions.

***Response:*** *In accordance with the PLOS ONE guidelines, all blots are being submitted as uncropped and unadjusted figures. These figures are presented as supplemental figures within the supporting information.*

**Comment 3:** PLOS requires an ORCID iD for the corresponding author in Editorial Manager on papers submitted after December 6th, 2016. Please ensure that you have an ORCID iD and that it is validated in Editorial Manager. To do this, go to ‘Update my Information’ (in the upper left-hand corner of the main menu), and click on the Fetch/Validate link next to the ORCID field. This will take you to the ORCID site and allow you to create a new iD or authenticate a pre-existing iD in Editorial Manager. Please see the following video for instructions on linking an ORCID iD to your Editorial Manager account: https://www.youtube.com/watch?v=_xcclfuvtxQ

***Response:*** *I have reconfirmed my ORCID ID and validated it in* *Editorial Manager, in accordance with the recommended procedure.*

**Comment 4:** We note that you have included the phrase “data not shown” in your manuscript. Unfortunately, this does not meet our data sharing requirements. PLOS does not permit references to inaccessible data. We require that authors provide all relevant data within the paper, Supporting Information files, or in an acceptable, public repository. Please add a citation to support this phrase or upload the data that corresponds with these findings to a stable repository (such as Figshare or Dryad) and provide and URLs, DOIs, or accession numbers that may be used to access these data. Or, if the data are not a core part of the research being presented in your study, we ask that you remove the phrase that refers to these data.

***Response:*** *As recommended by you, we have included an ELISA of TGFβ−1 for the three cell lines (Fig 7A, line 8). Because we observed that bFGF was not induced in BEAS-2B and 16HBE after incubation with CS CM, we did not perform a bFGF ELISA for NL20. Accordingly, we have omitted the text and reference concerning bFGF in the revised manuscript.*

**Comment 5:** Please review your reference list to ensure that it is complete and correct. If you have cited papers that have been retracted, please include the rationale for doing so in the manuscript text, or remove these references and replace them with relevant current references. Any changes to the reference list should be mentioned in the rebuttal letter that accompanies your revised manuscript. If you need to cite a retracted article, indicate the article’s retracted status in the References list and also include a citation and full reference for the retraction notice.

***Response:*** *As advised, we have carefully reviewed the citations and reference list. None of the papers cited in the revised manuscript has been retracted.*

Thank you very much for giving us the opportunity to improve our manuscript. We have endeavored to incorporate your feedback and hope that the revisions made by us persuade you to accept our submission.

Sincerely,

Motoo Katabami

**Responses to the comments made by Reviewer #1:**

Interesting study, with a lot of data. The reading is dense, and the results highlight some interesting track of understanding of the crystalline silica effects.

Here are some points to be explained or modified for a better/easier comprehension:

***Response:*** *We wish to express our sincere appreciation to Reviewer #1 for insightful comments and valuable suggestions, which have helped us significantly improve the manuscript.*

**Comment 1:** Avoid abbreviation in the abstract (i.e. CM, BPDE, NtAbs), this would facilitate the reading.

***Response:*** *Thank you for your advice. We have omitted the abbreviations from the abstract.*

**Comment 2:** Student's test is not so appropriate for a small number of replicate.

***Response:*** *Thank you for your pertinent comment. The Welch’s t-test is often more adequate than the Student’s t-test, especially for studies with duplicate measurements. In view of your comment, we performed an F-test for all statistical analyses. When we obtained unequal variance using this test, we performed the Welch’s t-test, instead of the Student’s t-test. Accordingly, we have revised the relevant text in the “Statistical analysis” section. When we judged an unequal variance using the F-test, we used the Welch’s t-test, instead of the Student’s t-test, for the next statistical evaluation. Consequently, the p-value <0.01 has been corrected to <0.05 for the following data:*

*“IL-6 ELISA for NL20 CS CM five days” in Fig 7A line 5 column 1.*

*Only one data set of MTT for “BEAS-2B CS CM 5 days” changed from significant to non-significant. Therefore, we have presented another independent data set showing significance using a sequential F-test to t-test analysis (revised Fig 1 line 2 column 2).*

**Comment 3:** Materials and methods section:

references of the reagents and the cell line are missing,

Some information is not clear about the reagent (storage, concentration, sterilization...) the crystalline silica is sterilized under its solid form?

How the applied doses (CS, BPDE) have been chosen? Is it physiologically relevant compared with human smokers or workers/miners?

***Response:*** *Thank you for your important remarks.*

*1) After a careful perusal, we have cited papers that adequately describe each reagent Min-U-Sil5, BPDE, and three bronchial cell lines (Reference numbers 15–19).*

*2) Doses of CS as high as 300 μg/cm^2^ chosen by us are within the range described in previous in vitro studies (Geys J, Nemery B, Hoet PH. Assay conditions can influence the outcome of cytotoxicity tests of nanomaterials: better assay characterization is needed to compare studies. Toxicol In Vitro. 2010 Mar;24(2):620-9). As you pointed out, these doses appear unphysiological and higher than the exposure limit of 0.05 mg/m^3^ CS recommended by NIOSH to prevent silicosis and lung cancer. However, to the best of our knowledge, the formula for conversion between μg/cm^2^ and mg/cm^3^ is not yet established. Therefore, we believe that it remains undetermined whether the doses of CS that we chose for in vitro study may be physiologically relevant or not with regard to in vitro and in vivo extrapolation. Similarly, the doses of BPDE used by us have previously been used for in vitro studies (Li D, Firozi PF, Chang P, Wang LE, Xiong P, Sturgis EM, et al. In vitro BPDE-induced DNA adducts in peripheral lymphocytes as a risk factor for squamous cell carcinoma of the head and neck. Int J Cancer. 2001;93:436-40). However, experiments using lower doses of CS and BPDE are awaited.*

**Comment 4:** Results:

For the quantification of soluble factors, explain why the incubation time was 4-6 days.

The protocol of cell exposure for all the experiments needs to be clarified (5 days for anchorage-independent growth, 4-6 days for soluble factor quantification), all the incubation times make the comprehension difficult.

***Response:*** *Thank you for your important questions. We have added data showing significant promotion of growth after 4–6 days of incubation with autocrine CS CM in both intact and CS-exposed cell lines in revised Fig 1 and Fig 2B. These data sets show that the timings for the quantification of soluble factors after 4 to 6 days of administration are concordant with the period when cell lines with CS CM grow more rapidly than those with control CM. Therefore, it may be reasonable to measure the concentration of soluble factors and growth of cell lines within 4–6 days after incubation with CS CM.*

**Comment 5:** 1) Is there a negative control for particle uptake? For example with Cyclin and cdc2 or c-myc, the expression is modified with the CS CM, is it due to CS or to the presence of particles inside the CM?

2) The gene expression was investigated, what about metabolism? Did the cells proliferate?

***Response:*** *1) As described in the section “Preparation of conditioned medium” under “Materials and Methods,” CS CM becomes free from CS particles upon filtration prior to use. Therefore, we suppose that the protein expression may not be affected by the mere presence of particles inside the CM.*

*2) After incubation with CS CM or BPDE CM, proteins were extracted from nonadherent bronchial cell lines under a growth-promoting condition. Conversely, after direct exposure to CS or BPDE, proteins were extracted from adherent bronchial cell lines under a growth-suppressive condition.*

**Comment 6:** Discussion:

A scheme of cascade response should be added to facilitate the comprehension of the different actors (cMyc, cdc2, PARP1, gH2AX, BRD4, EZH2, EGF, TNFa, IL-6) Maybe the authors can add a summary table with the up/down-regulation for all of the target studied.

***Response:*** *Thank you for your constructive advice. We have presented a scheme of the cascade response in Fig 8 as “a hypothetical pathway of CS-induced carcinogenesis.” Also, Table 1 shows a summary list of protein expression patterns. Although we could not include PARP1, γH2AX, and the condition of paracrine CM in Table 1, we have included all in Fig 8.*

**Comment 7:** Figures:

Be sure that the size and the quality of the figures allow an easy reading

***Response:*** *We have adjusted the figures carefully for clarity.*

**Comment 8:** Fig 2: A) explain the 12h or 24h of exposure,

A and B) when it is possible, use similar absorbance scales (for comparison between cell types)

***Response:*** *(1) Growth of adherent NL20 and BEAS-2B cells was significantly suppressed after 12 h of exposure to CS. On the contrary, a 24 h CS exposure was required to significantly suppress the growth of adherent 16HBE. We chose the above exposure times based on these observations. However, we only observed it under a microscope and did perform analyses, such as an MTT assay, after a 12 h exposure of adherent 16HBE cells to CS.*

*(2) Thank you for your advice. We agree with the point made by you. Adjusting the absorbance scale makes it easier to compare the degree of growth not only among cell lines at the same period but also throughout the time course for the same cell line. However, we performed the MTT assay at a single time point after incubation with CM in a manner independent of each other. In addition, the absorbance scale tends to fluctuate even with the same starting number of cells and with the same amount of time after incubation. We present below an example of this fluctuation. We would be obliged if you can take this into account.*

**Comment 9:** Fig 3

Same as previously, explain the 4-5-6 days of incubation

***Response:*** *As indicated in revised Fig 1 and Fig 2B, we analyzed protein expression, 4**–6 days after incubation with CM, which corresponds to the timing of continued growth of cell lines. Therefore, the estimation of protein expression, 4–6 days after incubation with CM, may be within the period when the bronchial cell lines showed an increase in growth. Therefore, we present another independent data showing significance as determined using F-test and t-test analysis sequentially.*

**Comment 10:** On the third line: modify legend - 4 days CS-exposed NL20 BPDE CM

Fig 5A and B: add the figure letters on the images.

Fig 6

Why is Fig6A duplicated in 6B? This is confusing - one figure (6B) will facilitate the reading

Just combined Fig6A and Fig6B to Fig6.

Fig 7B and 7C: add the figure letters

***Response:*** *Thank you for your helpful advice. We have revised the figures, as suggested.*

**Comment 11:** Fig 7C: TNF for 16HBE cells, EGF for NL20 and BEAS-2B cells, explain these choices

***Response:*** *Thank you for your query. As indicated in revised Fig 7A, TNF-α was not upregulated in culture supernatants of nonadherent NL20 and BEAS-2B after incubation with CS CM. Likewise, we also show in revised Fig 7A that EGF was not increased in culture supernatants of nonadherent 16HBE after incubation with CS CM. Therefore, we investigated the expression of BRD4 and EZH2 after administration of recombinant TNF-α only for 16HBE and after administration of recombinant EGF only for NL20 and BEAS-2B.*

**Response to Reviewer #2:**

The authors presented clearly the subject of their research with various hypothesis which have been validated by relevant experiments.

**Comment 1:** Some minor corrections are provided below:

- There is no indication on the passages used for the different cell lines for the experiments performed and presented in this publication. This addition could provide additional insights in the understanding of the molecular mechanisms.

***Response:*** *NL20 and BEAS-2B were passaged twice a week, whereas 16HBE was passaged weekly. We have mentioned the passage numbers in Materials and Methods section as follows:*

“NL20 cells were maintained in F-12K medium (GIBCO, Grand Island, NY) with 2.7 g/L glucose, 0.1 mM nonessential amino acids, 5 μg/mL insulin, 10 ng/mL EGF, 1 μg/mL transferrin, 500 ng/mL hydrocortisone, and 4% fetal bovine serum (FBS), and were passaged twice a week [18]. BEAS-2B cells, with a doubling time of approximately 26 h, were maintained in a serum-free LHC-9 medium containing 50 ng/mL EGF and were passaged twice a week [19]. The 16HBE14o- (16HBE) cells were cultured in Eagle’s minimum essential medium (EMEM) (Invitrogen Life Technologies Inc., Carlsbad, CA, USA) supplemented with 10% FBS and were passaged weekly.” (page 5, lines 110–117)

**Comment 2:** -The exposure is described as 5 days with some data at day 4 and day 6 but there is no indication of the typical cell doubling times of the different cell lines, and the potential impact of the treatment. This addition could be useful to reinforce the conclusion presented in the article.

***Response:*** *Thank you for your important suggestion. Although doubling time of BEAS-2B has been reported to be 26 h (J H Schiller, G Bittner, T D Oberley, C Kao, C Harris, L F Meisner. Establishment and Characterization of an SV40 T-Antigen Immortalized Human Bronchial Epithelial Cell Line. In Vitro Cellular & Developmental Biology. 1992;28A;461-464.), we could not find reports on the doubling times of NL20 and 16HBE. Thus, we have added the information only about the doubling time of BEAS-2B under Materials and Methods as follows:* “BEAS-2B cells, with a doubling time of approximately 26 h, were maintained in a serum-free LHC-9 medium containing 50 ng/mL EGF, and were passaged twice a week [19].” (page 5, lines 113–115) *Moreover, it was shown that cells with longer doubling times are more susceptible to injury induced by exposure to nanoparticles of silica (Lujan H, Criscitiello MF, Hering AS, Sayes CM. Refining In Vitro Toxicity Models: Comparing Baseline Characteristics of Lung Cell Types. Toxicol Sci. 2019;168: 302-314). In this respect, it is interesting to investigate whether the doubling times of NL20, BEAS-2B, and 16HBE may be associated with their CS-induced cytotoxicity.*

**Comment 3:**- in the article, the authors mentioned that "When the cells reached sub-confluent to confluent density, the medium was replaced with a CS-containing medium." Is there a strong impact for a same surface of the number of cells present to reach sub confluent to confluent density, and if yes is it a variable which should be adjusted to compare the data through the different cell models and conditions?

***Response:*** *As described in “Preparation of conditioned medium,” 48 or 72 h after adherent cell lines, having sub-confluent to confluent density, were incubated with CS-containing medium. Each collected medium was filtered for use as CS CM. As these CS CM obtained from sub-confluent to confluent cells reproducibly enhanced anchorage-independent growth of bronchial cell lines, as long as the growth of cells was significantly suppressed (and they were also damaged), CS CM was obtained for use. However, as you pointed out, further investigation is required to determine the factors, including the number of attached CS particles per cell surface, that are crucial to potentiate CS CM-induced cell growth.*

**Comment 4:**- finally for gammaH2aX data, it could be useful to have some confocal images showing the potential variation in terms of foci density per nucleus ( or absence of this variation) to better assess the relevance of the presented data.

***Response:*** *We agree with your contention. Other approaches, such as confocal images of γH2aX, may make our data more relevant. Therefore, we have added the following sentence explaining that this analysis is needed in the future:* “Further investigation is warranted to demonstrate whether other NHEJ-related genes besides γH2AX are aberrantly expressed in bronchial cells undergoing both repeated exposure to CS and repeated treatment with CS and/or BPDE CM using western blot and fluorescent microscopy analyses.” (page 21, lines 473–476)
